# A draft genome assembly for the eastern fox squirrel, *Sciurus niger*

**DOI:** 10.1093/g3journal/jkab315

**Published:** 2021-09-22

**Authors:** Lin Kang, Pawel Michalak, Eric Hallerman, Nancy D Moncrief

**Affiliations:** 1 Edward Via College of Osteopathic Medicine, University of Louisiana Monroe, Monroe, LA 71203, USA; 2 Center for One Health Research, VA-MD Regional College of Veterinary Medicine, Blacksburg, VA 24060, USA; 3 Institute of Evolution, University of Haifa, Haifa 3498838, Israel; 4 Department of Fish and Wildlife Conservation, Virginia Polytechnic Institute and State University, Blacksburg, VA 24061, USA; 5 Virginia Museum of Natural History, Martinsville, VA 24112, USA

**Keywords:** whole-genome sequencing, *de novo* assembly, tree squirrels, candidate genes, heme biosynthesis, color vision, hibernation, eastern fox squirrel, *Sciurus niger*, Sciuridae

## Abstract

The eastern fox squirrel, *Sciurus niger*, exhibits marked geographic variation in size and coat color, is a model organism for studies of behavior and ecology, and a potential model for investigating physiological solutions to human porphyrias. We assembled a genome using Illumina HiSeq, PacBio SMRT, and Oxford Nanopore MinION sequencing platforms. Together, the sequencing data resulted in a draft genome of 2.99 Gb, containing 32,830 scaffolds with an average size of 90.9 Kb and N_50_ of 183.8 Kb. Genome completeness was estimated to be 93.78%. A total of 24,443 protein-encoding genes were predicted from the assembly and 23,079 (94.42%) were annotated. Repeat elements comprised an estimated 38.49% of the genome, with the majority being LINEs (13.92%), SINEs (6.04%), and LTR elements. The topology of the species tree reconstructed using maximum-likelihood phylogenetic analysis was congruent with those of previous studies. This genome assembly can prove useful for comparative studies of genome structure and function in this rapidly diversifying lineage of mammals, for studies of population genomics and adaptation, and for biomedical research. Predicted amino acid sequence alignments for genes affecting heme biosynthesis, color vision, and hibernation showed point mutations and indels that may affect protein function and ecological adaptation.

## Introduction

Squirrels are among the most familiar wild mammals in the world (Thorington *et al.* 2012), and tree squirrels (Sciuridae; Sciurini) comprise one of the most rapidly diversifying lineages of mammals (de Abreu *et al.* 2020). The eastern fox squirrel (*Sciurus niger*; hereafter fox squirrel) is widespread over most of North America east of the Rocky Mountains (Koprowski 1994). This species exhibits marked geographic variation in size (from 500 to 1500 g) and striking patterns of geographically structured variation in coat color (Weigl *et al.* 1998). In addition, fox squirrels exhibit melanism as the result of mutations at two independent loci (McRobie *et al.* 2019).

Fox squirrels are also potential models for investigating physiological solutions to human porphyrias, diseases caused by defects in the enzymes of the heme biosynthetic pathway, because fox squirrels seem to be adapted to accumulate porphyrins without injurious consequences (Levin and Flyger 1973). Fox squirrels sequester excess uroporphyrin I in their bones, which then fluoresce pink under ultraviolet light (Turner 1937; Flyger and Levin 1977). Even bones preserved in archaeological and fossil deposits fluoresce, indicating that this condition has persisted in fox squirrels for at least several thousand years (Dooley and Moncrief 2012; Moncrief and Dooley 2013). Elucidating the underlying genetic network that allows fox squirrels to avoid porphyrin pathenogenicity may complement the use of laboratory models and provide new insights into the treatment of these disorders in humans (de Oliveira Neves and Galván 2020), which may drive a broad spectrum of neurological symptoms (O’Malley *et al.* 2018).

Squirrels are highly visual rodents that may be excellent model systems for understanding mechanisms of function and disease in the human visual system (Van Hooser and Nelson 2006). They use vision for navigating in their environment, predator avoidance, and foraging for food. Unlike the more traditional mouse and rat model systems, most squirrel species are diurnal with cone-dominated retinas, similar to those of primates, and have excellent dichromatic color vision that is mediated by green and blue cones. Many basic anatomical and physiological properties in the visual system of squirrels have now been described, permitting investigations of cellular mechanisms. While the genes responsible for color vision deficiencies in humans are reasonably well known (El Moussawi *et al.* 2021), their variation within and among squirrel species remain uncharacterized.

Hibernation in squirrels is a dynamic phenotype, with timing set by an annual clock. Noting that captive 13-lined ground squirrels (*Ictidomys tridecemlineatus*) exhibited variation in the seasonal onset of hibernation, Grabek *et al.* (2019) hypothesized that genetic factors drive variation in hibernation timing. They applied genotyping-by-sequencing to characterize genetic variation in 153 individuals and estimated high heritability (>61%) for hibernation onset. Applying a genome-wide scan with 46,996 SNP variants, they identified two loci significantly and 12 loci suggestively associated with hibernation onset. At the most significant locus, whole-genome resequencing revealed a putative causal variant in the promoter of *FAM204A*. Expression quantitative trait loci analyses revealed gene associations for 8 of the 14 loci.

To date, genomes have been published for five ground squirrels (Sciuridae; Xerini, Marmotini), and two tree squirrels in the genus *Sciurus*: the Eurasian red squirrel (*Sciurus vulgaris*) and the eastern gray squirrel (*Sciurus carolinensis*, Table 1). The fox squirrel genome assembly that we report will serve as a reference genome for this species, and it will allow further genomic, proteomic, and phylogenetic comparisons among tree squirrels and other sciurids, as well as other rodents and mammals.

**Table 1:** Publicly available genomes used for phylogenetic analysis of *Sciurus niger* (eastern fox squirrel)

Species	Common name	NCBI accession	Reference
*Ictidomys tridecemlineatus*	Thirteen-lined ground squirrel	GCF_000236235.1	Lindblad-Toh *et al.* (2011) and Broad Institute (2021a)
*Urocitellus parryii*	Arctic ground squirrel	GCF_003426925.1	Goropashnaya *et al.* (2020)
*Spermophilus dauricus*	Daurian ground squirrel	GCA_002406435.1	Koepfli *et al.* (2015) and Genome 10K Community of Scientists (2021)
*Marmota monax*	Woodchuck or groundhog	GCA_901343595.1	Alioto *et al.* (2019)
*Xerus inauris*	South African ground squirrel	GCA_004024805.1	Broad Institute (2021b)
*Sciurus carolinensis*	Eastern gray squirrel	GCA_902686445.2	Mead *et al.* (2020b)
*Sciurus vulgaris*	Eurasian red squirrel	GCA_902686455.2	Mead *et al.* (2020a)
*Aplodontia rufa*	Mountain beaver	GCA_004027875.1	Broad Institute (2021b)

## Materials and methods

### Sample and DNA extraction

A male fox squirrel was obtained from the wild in Allegan County, Michigan (coordinates 42.641749°N, 85.886986°W) on December 21, 2018 and archived as a voucher specimen at the Virginia Museum of Natural History (NDM4471 and VMNH3098). The tissue sample from skeletal muscle was stored in RNAlater solution (ThermoFisher Scientific) and frozen at -80°C until DNA extraction. The genomic DNA was isolated using Puregene Cell & Tissue Kit (Qiagen) following the manufacturer’s protocol for purification of total DNA from animal tissues.

### Genome assembly and sequencing

DNA sequence reads were generated using three sequencing platforms. A TruSeq DNA library was prepared and sequenced on the HiSeq platform following Illumina’s protocols, and two lanes of 2 × 150-bp paired-end reads were generated (242.8 Gb). A PacBio SMRT (single molecule real time) library with Sequel chemistry was prepared and sequenced on three SMRT cells (19.0 Gb). Finally, genomic DNA was sequenced using the Oxford Nanopore MinION system. Libraries were made using the VolTRAX Sequencing Kit (VSK-VSK002) and were sequenced on two FLO-MIN 107 R9 flow cells (18.3 Gb). The raw sequence reads from Illumina, PacBio, and Oxford Nanopore were used as the input to generate a *de novo* assembly using the MaSuRCA assembler v3.3.2 (Zimin *et al.* 2013).

### Gene prediction and annotation

Repeat families were identified by using the *de novo* modeling package RepeatModeler v1.0.8 (http://www.repeatmasker.org/RepeatModeler). Then, the *de novo* identified repeat sequences were combined with manually selected mammalian repeats from RepBase v22.12 (https://www.girinst.org/repbase) and a customized repeat library was formed. Before the gene prediction, the draft assembly was first masked using RepeatMasker v4.0.3 (http://www.repeatmasker.org/) with parameters set to “-s -a -nolow” and using the customized repeat library. Protein-encoding genes were predicted using MAKER2 (Holt and Yandell 2011), which integrates prediction methods including BLASTX, SNAP (Korf 2004), and Augustus (Stanke and Waack 2003). The Augustus model file was generated by training the core genes of Mammalia from the genome completeness assessment tool BUSCO (Benchmark Universal Single-Copy Orthologs; Simão *et al.* 2015). Predicted genes were subsequently used as query sequences in a BLASTX database search of the NR database (the nonredundant database, http://www.ncbi.nlm.nih.gov) with *e*-value cutoff of 1*e*^−20^ and percent identity cutoff of 50. The top hit of BLASTX alignments with the lowest *e*-value was used to annotate the query genes.

### Genome completeness

For genome completeness estimation, BUSCO (Simão *et al.* 2015) was used to assess 4,104 universal single-copy orthologs of Mammalia in the assembly.

### Phylogenetic analyses

Protein sequences of 4,104 Mammalia core genes were extracted from BUSCO (Simão *et al.* 2015) for analysis of *S. niger* and eight other publicly available genomes (Table 1). Sequences of these core genes were concatenated and then aligned using MAFFT v. 7.475 (Rozewicki *et al.* 2019). The phylogenetic tree was reconstructed using Bayesian inference in BEAST 2.51 (Bouckaert *et al.* 2014). In the BEAST analysis, a strict clock was selected, and the Yule process of speciation was selected as tree prior. The BEAST Markov chain Monte Carlo run was conducted for 5 million generations, and the first half-million generations were set as burn-in. The consensus tree was inferred by TreeAnnotator 2.51 (Bouckaert *et al.* 2014). The divergence time of 52.30 MYA (CI 48.94–55.67 MYA, Menéndez *et al.* 2021) between *Aplodontia rufa* and Sciuridae was used for the calibration.

### Sequence alignments

Alignments of candidate genes for heme biosynthesis, color vision, and control of hibernation were inspected for variation among *Sciurus* sp. and other sciurids. Details of gene function were obtained from GeneCards (2021), a database of information on all annotated and predicted human genes.

## Results and discussion

### Assembly and annotation of the fox squirrel genome

A total of 242.8 Gb Illumina, 18.0 Gb PacBio, and 18.3 Gb Oxford Nanopore sequencing data were generated. Together, the sequencing data resulted in a draft genome of 2.99 Gb, which contains 32,830 scaffolds with an average size of 90.9 Kb and N_50_ of 183.8 Kb (Table 2). The genome completeness estimated by BUSCO (Simão *et al.* 2015) was 83.74% (C) + 10.04% (F) [Complete = 83.74% (Single = 81.94%, Duplicated = 1.80%), Fragmented = 10.04%, Missed = 6.22%, Gene = 4,104; Table 2]. A total of 24,443 protein-encoding genes were predicted from the assembly (Table 3), and 23,079 (94.42%) were annotated (Table 2).

**Table 2:** Summary of the composite genome assembly of *Sciurus niger* (eastern fox squirrel)

Assembly attributes	
Total size	2,985,236,946 bp
No. of scaffolds	32,830
No. of scaffolds > 10 Kb	30,561
Scaffold N_50_	183,784 bp
Longest scaffold	1,891,617 bp
GC content	44.00%
Gene annotation	
No. of predicted genes	24,443
No. (percentage) of annotated genes	23,079 (94.42%)
Completeness (BUSCO)	
Complete	83.74%
Fragmented	10.04%
Missed	6.22%

**Table 3:** Summary statistics for 24,443 predicted protein-coding genes in *Sciurus niger* (eastern fox squirrel)

Statistic	Number
Average gene length (bp)	17,072
Average CDS length (bp)	1,336
Average exons per gene	6.9
Average exon length (bp)	195
Average intron length (bp)	2,677

### Repeated genomic elements

**Figure 1:**
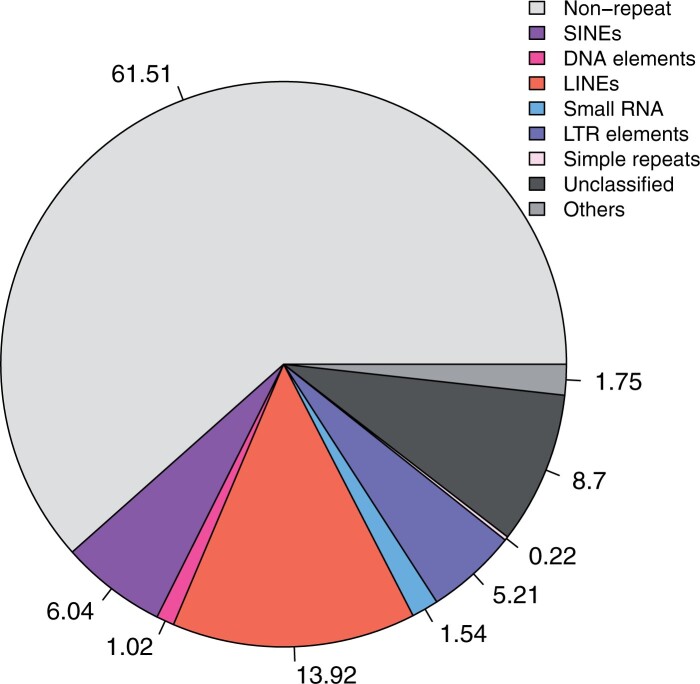
Repeat content of the *Sciurus niger* (eastern fox squirrel) genome.

The estimated percentage of repeat elements in the genome is 38.49% (Figure 1 and Supplementary File S1) with the majority being LINEs (13.92%), SINEs (6.04%), and LTR elements (5.21%).

### Phylogenomic relationships

**Figure 2:**
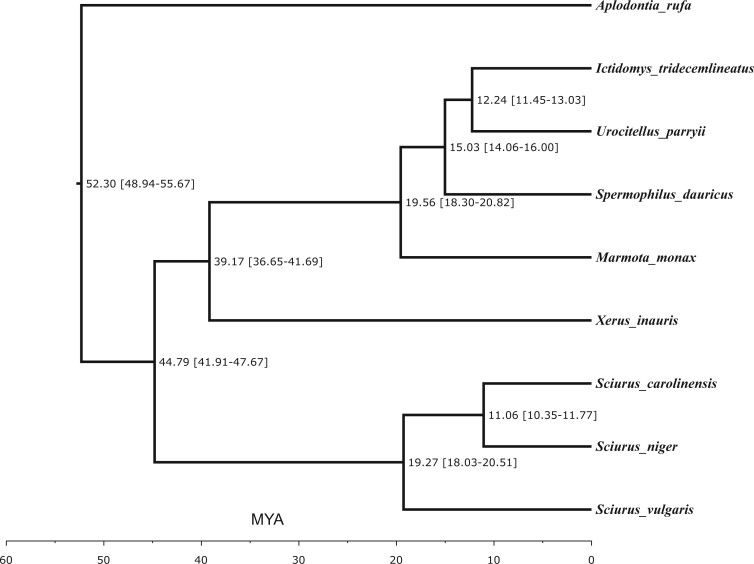
Molecular phylogeny of eight squirrel species, including *Sciurus niger* (eastern fox squirrel), rooted using *Aplodontia rufa*, mountain beaver. Branch lengths in millions of years ago (± 95% CI) were estimated on the basis of numbers of amino acid substitutions per site.

Maximum-likelihood phylogenetic analysis of relationships of *S. niger* and seven other sciurids, using mountain beaver (*A. rufa*) as an outgroup, generated a species tree (Figure 2). *Sciurus niger* clustered most closely with *S. carolinensis* and *S. vulgaris*, with an estimated divergence time of 19.27 MYA. The congeneric tree squirrels clustered separately from the ground squirrels, with an estimated divergence time of 44.79 MYA. The topology of this species tree is congruent with those of previous studies (Zelditch *et al.* 2015; Menéndez *et al.* 2021).

### Sequence alignments for selected genes

Predicted amino acid sequence alignments for 17 genes (9 involved with heme biosynthesis, 5 with color vision, and 3 with regulation of hibernation) are presented in Supplementary Files S2–S4, respectively. Point differences among sequences were observed in all sequence alignments among *Sciurus* species, as well as among *Sciurus* sp. and the other rodents. Amino acid sequences were highly conserved among members of the genus *Sciurus* at *ALAS1*, *ALAS2*, *FECH*, *UROS*, and among all species studied at *CPOX*, *GNAT2*, *EXOC4*, and large segments of *CNGA3* and *OPN1MW*. We observed insertions or deletions of multiple contiguous amino acids among *Sciurus* sp. at *ALAD*, *HMBS*, *PPOX*, *UROD*, *UROS*, *ATF6*, *PDE6C*, *CHCHD3*, and *FAM204A.* We observed insertions or deletions of multiple contiguous amino acids among *Sciurus* sp. and the other rodents at *ALAD*, *ALAS2*, *FECH*, *HMBS*, *PPOX*, *UROD*, *ATF6*, *CNGA3*, *PDE6C*, *CHCHD3*, and *FAM204A.* Future work might address the functional significance of these differences in terms of protein function and ecological adaptation.

## Data availability

This genome assembly has been deposited at DDBJ/ENA/GenBank under the accession JAHUXG000000000. The version described in this paper is version JAHUXG010000000. All sequencing data were deposited at NCBI SRA (accession number: PRJNA744496).

Supplementary material is available at *G3* online.

## Supplementary Material

jkab315_Supplementary_File_S1

jkab315_Supplementary_File_S2

jkab315_Supplementary_File_S3

jkab315_Supplementary_File_S4
