# Supplementary material for: A draft genome assembly for the eastern fox squirrel, Sciurus niger
Source: G3 (Bethesda). 2021 Sep 22;11(12):jkab315. doi: 10.1093/g3journal/jkab315 (PMC8664420; doi:10.1093/g3journal/jkab315)
Supplement: jkab315_Supplementary_File_S1 [file jkab315_supplementary_file_s1.pdf]

Supplemental File S1: Repeat elements annotated via RepeatMasker.

Total bases masked: 1,149,011,319 bp (-38.49 %)

Category		Number of elements	Length occupied (bp)	Percentage of sequences
SINEs		1,197,621	180,422,092	6.04%
	ALUs	386,199	56,935,568	1.91%
	MIRs	18,848	11,685,270	0.39%
LINEs		619,802	415,506,401	13.92
	LINE1	605,782	408,535,904	13.69
	LINE2	4,642	610,358	0.02
	L3/CR1	270	57,418	0
LTR elements		283,931	155,612,809	5.21
	ERVL	42,518	17,298,594	0.58
	ERVL-MaLRs	71,989	19,236,303	0.64
	ERV_classI	93,607	56,581,945	1.9
	ERV_classII	67,538	53,571,420	1.79
DNA elements		105,824	30,448,642	1.02
	hAT-Charlie	15,446	3,707,175	0.12
	TcMar-Tigger	6,504	2,017,185	0.07
Unclassified		688,337	259,814,986	8.7
Small RNA		447,950	46,068,017	1.54
Satellites		12,635	2,834,259	0.09
Simple repeats		2,653	6,456,508	0.22
