# Supplementary material for: A draft genome assembly for the eastern fox squirrel, Sciurus niger
Source: G3 (Bethesda). 2021 Sep 22;11(12):jkab315. doi: 10.1093/g3journal/jkab315 (PMC8664420; doi:10.1093/g3journal/jkab315)
Supplement: jkab315_Supplementary_File_S2 [file jkab315_supplementary_file_s2.pdf]

Supplemental File S2 – Heme Biosynthesis

Synthesis of heme is critical to hemoglobin formation and oxygen transport. In a normal man, 6.3 g of hemoglobin is replaced daily with little wastage; porphyrias result from disruption of synthesis or feedbacks in process control, often due to deficiency of one or more enzymes in the synthesis pathway (Rimington 1989). Defects in each of the eight enzymes in the heme biosynthetic pathway have been associated with a specific porphyria (Phillips 2019). We examined variation at nine genes (O'Malley *et al*. 2018) among sciurids.

The *ALAD* gene encodes aminolevulinate dehydratase. Diseases associated with *ALAD* include porphyria, and acute hepatic porphyria. Among its related pathways are porphyrin and innate immune system response. The ALAD enzyme is composed of eight identical subunits and catalyzes the condensation of two molecules of δ-aminolevulinate to form porphobilinogen (a precursor of heme). *ALAD* catalyzes the second step in the porphyrin and heme biosynthetic pathway. ALAD enzymatic activity is inhibited by lead and a defect in the *ALAD* structural gene can cause increased sensitivity to lead poisoning and acute hepatic porphyria. Alternative splicing of this gene results in multiple transcript variants encoding different isoforms.

Our sequence alignment included 355 predicted amino acids for *S. niger*. There were differences at 16 amino acids among *Sciurus* species; additionally, *S. vulgaris* lacked 23 amino acids at positions 270-293. Three ground squirrels – *S. dauricus*, *I. tridecemlineatus*, and *U. parryii* - had a 32-amino acid sequence at positions 140-172 not seen in other species.

```
Alad            MHHQSVLHSGYFHPLLRSWQTAASTVSASNLIYPIFVTDVPDDVQPIASLPGVARYGVNQ
Aplodontia_rufa MQPQSILHSGYFHPLLRTWQTAATTLSASNLIYPIFVT-VSKN-GPDLSAQGGGRCGTGQ
Xerus_inauris@A MQPQSVLHSGYFHPLLRTWQAAATSLSASNFIYPIFVT-VPRNVQPIASLPGVARYGVNQ
Marmota_monax@A MQPQSVLHSGYFHPLLRTWQTAATTLSASNFIYPIFVT-VPDDPASPQSLPSPPRYGVNR
Spermophilus_da MQPQSVLHSGYFHPLLRTWQTAATTLSASNFIYPIFVT-VPRN-GPDLSVHGGGRYGVNQ
Ictidomys_tride MQPQSVLHSGYFHPLLRTWQTAATTLSASNFIYPIFVT-VPDDVQPPQSLPSPPRYGVNR
Urocitellus_par MQPQSVLHSGYFHPLLRTWQTAATTLSASNFIYPIFVT-VPRNVQPPQSLPSPPRYGVNR
Sciurus_vulgari MQPQSVLHSGYFHPLLRTWQTAATTLSASNFIYPIFVTDVPDDIQPIASLPVMARYRVNR
Sciurus_carolin MQPQSVLHSGYFHPLLRTWQTAATTLSASNFIYPIFVTDVPDDIQPIASLPGMSRYGVNQ
Sciurus_niger@A MHPQSVLHSGYFHPLLRTWQTAATTLSASNFIYPIFVTDVPDDIQPIANLPGMSRYGVNQ
                *: **:***********:**:**:::****:******* *. :  .  .     *  ..:

Alad            LEEMLRPLVEAGLRCVLIFGVPSRVPKDEQGSAADSEDSPTIEAVRLLRKTFPSLLVACD
Aplodontia_rufa LEEMLRPLVEAGLRCVLIFGVPSRVPKDERGSAADSEESPAIEAIRLLRKTFPNLLVACD
Xerus_inauris@A LEEMLRPLVEAGLRCVLIFGVPSRVP-DERGSAADSEESPAIEAIRLLRKTFPSLLVACD
Marmota_monax@A LEEMLKPLVEEGLRCVLIFGVPSRVPKDEQGSAADSEESPTIEAVRLLRKTFPSLLVACD
Spermophilus_da LEEMLRPLVEAGLRCVLIFGVPSRCP-DERGSAADSEESPTIEAVRLLRKTFPSLLVACD
Ictidomys_tride LEEMLKPLVEEGLRCILIFGVPSRVPKDEQGSAADSEESPTIEAVRLLRKTFPSLLVACD
Urocitellus_par LEEMLKPLVEEGLRCVLVFGVPSRVPKDEQGSAADSEESPTIEAVRLLRKTFPSLLVACD
Sciurus_vulgari LEEMLRPLVEEGLCCVLIFGVPSKVPKDERGSAADSEKSPAIEAIRLLRKTFPNLLVACD
Sciurus_carolin LEEMLRPLVEAGLRCVLIFGVPSRVPKDERGSAADSEKSPAIEAIHLLRKTFPNLLVACD
Sciurus_niger@A LEEMLRPLVEAGLRCVLIFGVPSRVPKDERGSAADSEDSPTIEAVRLLRKTFPNLLVACD
                *****:**** ** *:*:*****: * **:*******.**:***::*******.******

Alad            VCLCPYTSHGHCGLLSENGAFLAEESRQRLAEVALAYAKA--------------------
Aplodontia_rufa VCLCPYTSHGHCGLLSENGAFRAEESRQRLAEVALAYAKA--------------------
Xerus_inauris@A VCLCPYTSHGHCGLLSENGAFRAEESRQRLAEVALAYAKA--------------------
Marmota_monax@A VCLCPYTSHGHCGLLSENGAFKAEESRQRLAEVALAYAKA--------------------
```

```
Spermophilus_da VCLCPYTSHGHCGLLSENGAFKAEESRQRLAEVALAYAKAGEVPRRGGHLWVGELAEWIE
Ictidomys_tride VCLCPYTSHGHCGLLSENGAFKAEESRQRLAEVALAYAKAGEVPSRGGHLWVGELAEWIE
Urocitellus_par VCLCPYTSHGHCGLLSENGAFKAEESRQRLAEVALAYAKAGEVPSRGGHLWVGELAEWIE
Sciurus_vulgari ICLCPYTSHGHCGLLSENGAFRAEESRQRLAEVALAYAKA--------------------
Sciurus_carolin ICLCPYTSHGHCGLLSENGAFRAEESRQRLAEVALAYAKA--------------------
Sciurus_niger@A ICLCPYTSHGHCGLLSENGAFRAEESRQRLAEVALAYAKA--------------------
                :******************** ******************

Alad            ------------GCQVVAPSDMMDGRVEAIKAALLKHGLGNRVSVMSYSAKFASCFYGPF
Aplodontia_rufa ------------GAQVVAPSDMMDGRVEAIKAALLKHGLGNRVSVMSYSAKFASCFYGPF
Xerus_inauris@A ------------GCQVVAPSDMMDGRVEAIKEALMAHGLGNRVSVMSYSAKFASCFYGPF
Marmota_monax@A ------------GCQVVAPSDMMDGRVEAIKEALMAHGLGNRVSVMSYSAKFASCFYGPF
Spermophilus_da GPRVLKTTLCPSGCQVVAPSDMMDGRVEAIKDALMAHGLGNRVSVMSYSAKFASCFYGPF
Ictidomys_tride GPRVLKTSLCPSGCQVVAPSDMMDGRVEAIKDALMAHGLGNRVSVMSYSAKFASCFYGPF
Urocitellus_par GPRVLKTTLCPSGCQVVAPSDMMDGRVEAIKDALLAHGLGNRVSVMSYSAKFASCFYGPF
Sciurus_vulgari ------------GCQVVAPSDMMDGRVEAIKEALMAHGLGNRVSVMSYSAKFASCFYGPF
Sciurus_carolin ------------GCQVVAPSDMMDGRVEAIKEALMAHGLGNRVSVMSYSAKFASCFYGPF
Sciurus_niger@A ------------GCQVVAPSDMMDGRVEAIKEALMAHGLGNRVSVMSYSAKFASCFYGPF
                            *.***************** **: ************************

Alad            RDAAQSSPAFGDRRCYQLPPGARGLALRAVA-----------------------------
Aplodontia_rufa RDAAQSSPAFGDRRCYQLPPGARGLALRAVA-----------------------------
Xerus_inauris@A RDAAQSSPAFGDRRCYQLPPGARGLALRAVVNDQAA---------THTHPLPA----YPL
Marmota_monax@A RDAAQSSPAFGDRRCYQLPPGARGLALRAVA-----------------------------
Spermophilus_da RDAAQSSPAFGDRRCYQLPPGARGLALRAVVSDQAD-------THTHTHTLPA----CPF
Ictidomys_tride RDAAQSSPAFGDRRCYQLPPGARGLALRAVVSDQAADTHTHTYTHTHTHTLPA----CPF
Urocitellus_par RDAAQSSPAFGDRRCYQLPPGARGLALRAVA-----------------------------
Sciurus_vulgari QDAALSSPTFGDSCCYQLPPGARGLALQAV------------------------------
Sciurus_carolin RDAAQSSPAFGDRRCYQLPPGARGLALRAVVSDQAA---------THSPPCLSPSRINAV
Sciurus_niger@A RDAAQSSPAFGDRRCYQLPPGARGLALRAVVSDQAA---------THSPPCLSPSRINAV
                :*** ***:***  *************:**

Alad            ---------RDIQEGADMLMVKPGLPYLDMV-REVKDKHPELPLAVYQVSGEFAMLWHGA
Aplodontia_rufa ---------RDIQEGADMLMVKPGLPYLDMV-REVKDQHPELPLAVYHVSGEYAMLWHGA
Xerus_inauris@A PRHCTLIPDRDVREGADMLMVKPGMPYLDIV-REVKDKHPELPLAVYHVSGEFAMLWHGA
Marmota_monax@A ---------RDIQEGADMLMVKPGLPYLDMV-REVKDQHPQLPLAVYHVSGEFAMLWHGA
Spermophilus_da PRHFTLFPDRDVREGADMLMVKPGLPYLDMV-REVKDKHPQLPLAVYHVSGEFAMLWHGA
Ictidomys_tride PRHFTLFPDRDVREGADMLMVKPGMPYLDIV-REVKDKHPQLPLAVYHVSGEFAMLWHGA
Urocitellus_par ---------RDIQEGADMLMVKPGLPHLNPVPMAASPQHPQLPLAVYHVSGEFAMLWHGA
Sciurus_vulgari --------DRDVQEGADMLMVKPGMPYLDIV-WEVKDKHPELPLAVYHVSGEFAMLWHGA
Sciurus_carolin VRHFTFIPDRDVQEGADMLMVKPGMPYLDIV-REVKDKHPELPLAVYHVSGEFAMLWHGA
Sciurus_niger@A VRHFTLIPDRDVQEGADMLMVKPGMPYLDIV-REVKDKHPELPLAVYHVSGEFAMLWHGA
                         **::***********:*:*: *   .. :**:******:****:*******

Alad            QAGAFDLRTAVLETMTAFRRAGADIIITYFAPQLLKWLKEEZ
Aplodontia_rufa RAGAFDLKAAVLEAMTAFRRAG--------------------
Xerus_inauris@A RAGAFDLKAAVLEAMTAFRRAG--------------------
Marmota_monax@A RAGAFDLKAAVLEAMTAFRRAG--------------------
Spermophilus_da RAGAFDLKAAVLEAMTAFRRAG--------------------
Ictidomys_tride RAGAFDLKAAVLEAMTAFRRAG--------------------
Urocitellus_par RAGAFDLKAAVLEAMTAFRRAG--------------------
Sciurus_vulgari QAGAFDLKAAVLEAMTAFRRAGADVIITYHTPQLLQWLKE--
Sciurus_carolin QAGAFDLKAAVLEAMTAFRRAGADIIITYYTPQLLQWLKE--
Sciurus_niger@A QAGAFDLKAAVLEAMTAFRRAGADIIITYYTPQLLRWLKE--
                :******::****:********
```

ALAS1 encodes 5'-aminolevulinate synthase 1. Diseases associated with *ALAS1* include acute porphyria and anemia, sideroblastic, 1. Among its related pathways are porphyrin and glycine, serine, and threonine metabolism. Gene ontology (GO) annotations related to this gene include pyridoxal phosphate binding and 5-aminolevulinate synthase activity. An important paralog of this gene is ALAS2. This gene encodes the mitochondrial enzyme which is catalyzes the rate-limiting step in heme (iron-protoporphyrin) biosynthesis. The enzyme encoded by this gene is the housekeeping enzyme; a separate gene encodes a form of the enzyme that is specific for erythroid tissue. The level of the mature encoded protein is regulated by heme; high levels of heme down-regulate the mature enzyme in mitochondria, while low heme levels up-regulate it. Alternative splicing results in multiple transcript variants encoding different isoforms.

The *ALAS1* amino acid sequence was rather highly conserved among sciurids and among all species considered. Among 640 amino acids for *Sciurus* species, we observed three point differences. The human sequence had a two-amino acid insertion at sites 84-85.

```
Alas1             METVVRRCPFLSRVPQAFLQKAGKSLLFYAQNCPKMMEVGAKPAPRTLSTSAVHCQQVKE
Aplodontia_rufa   METVVRRCPFLSRVPQAFLQKAGKSLLFYAQNCPKMMEVWSKPVPRALTTSAVHCQQIKE
Xerus_inauris@A   METVVRRCPFLSRVPQAFLQKAGKSLLFYAQNCPKMMEIGSKPAPRALSTSAVHCQQIKE
Marmota_monax@A   METVVRRCPFLSRVPQAFLQKAGKSLLFYAQNCPKMMEVGSKPVPRAVSTSAVHCQQIKE
Spermophilus_da   METVVRRCPFLSRVPQAFLQKAGKSLLFYAQNCPKMMEVGSKPVPRAVSTSAVHCQQIKE
Ictidomys_tride   METVVRRCPFLSRVPQAFLQKAGKSLLFYAQNCPKMMEVGSKPVPRAVSTSAVHCQQIKE
Urocitellus_par   METVVRRCPFLSRVPQAFLQKAGKSLLFYAQNCPKMMEVGSKPVPRAVSTSAVHCQQIKE
Sciurus_vulgari   METVVRRCPFLSRVPQAFLQKAGKSLLFYAQNCPKMMEVGSKPAPRALSTSAVHCQQIKE
Sciurus_carolin   METVVRRCPFLSRVPQAFLQKAGKSLLFYAQNCPKMMEVGSKPAPRALSTSAVHCQQIKE
Sciurus_niger@A   METVVRRCPFLSRVPQAFLQKAGKSLLFYAQNCPKMMEVGSKPAPRALSTSAVHCQQIKE
                  **************************************: :**.**:::********:**

Alas1             TPPANEKEKTAKAAVQQAPDESQMAQTPDGTQLPSGHPSPATSQGSGSKCPFLAAQLSQT
Aplodontia_rufa   TPPAGEEDKTAKAKVQETPDRSQ--QTPNGTQLPSAHLSPAMSQGTSSKCPFLAAQMSQT
Xerus_inauris@A   TPPASEEDKTAKTKVQQAPDRSQ--QTPNGMQLPSGHPLPAMSQGTASKCPFLAAQMSQT
Marmota_monax@A   TPPASEEDKTAKAKVQQAPDRSQ--QTPNGTQLPSGHHSPAMSQGTASKCPFLAAQMSQT
Spermophilus_da   TPPASEEDKTAKAKVQQAPDRSQ--QTPNGTQLPSGHHSPAMSQGTASKCPFLAAQMSQT
Ictidomys_tride   TPPASEEDKTAKAKVQQAPDRSQ--QTPNGTQLPPGHHSPAMSQGTASKCPFLAAQMSQT
Urocitellus_par   TPPASEEDKTAKAKVQQAPDRSQ--QTPNGTQLPSGHHSLAMSQGTASKCPFLAAQMSQT
Sciurus_vulgari   TPPASEDKTANKSKVQQAPDRSQ--QTPNGTQLPSGHPSPAMSQGTASKCPFLAAQMSQT
Sciurus_carolin   TPPASEDKTANKSKVQEAPDRSQ--QTPNGTQLPSGHPSPAMSQGTASKCPFLAAQMSQT
Sciurus_niger@A   TPPASEDKTANKSKVQQAPDRSQ--QTPNGTQLPSGHPSPAVSQGTASKCPFLAAQMSQT
                  ****.*...: *: **::**.**  ***:* ***..*   * ***:.*********:***

Alas1             GSSVFRKASLELQEDVQEMHAVRKEAAQSPVPPSLVNVKTDGEDPSRLLKNFQDIMRKQR
Aplodontia_rufa   GSSVFCKASLELQEDVQEMHAVRKEVAQTSVKPTVINVKTNGDDPSGLLKNFQDIMRKQR
Xerus_inauris@A   GSNVFCKASLELQEDVQEMHAVRKEVAQTSVNPSVISVKTDGKDPSELLKNFQDIMQKQR
Marmota_monax@A   GSNVFCKASLELQEDVQEMHAVRKEVAQTSVNPNVINVKTDGEDPSELLKNFQDIMRKQR
Spermophilus_da   GSNVFCKASLELQEDVQEMHAVRKEVAQTSVNPNVINVKTDGEDPSELLKNFQDIMRKQR
Ictidomys_tride   GSNVFCKASLELQEDVQEMHAVRKEVAQTSVNPNVINVKTDGEDPSELLKNFQDIMRKQR
Urocitellus_par   GSNVFCKASLELQEDVQEMHAVRKEVAQTSVNPNVINVKTDGEDPSELLKNFQDIMRKQR
Sciurus_vulgari   GSSVFCKASLELQEDVQEMHAVRKEVAQTSVNPNVISVKTDGEDPNELLKNFQDIMRKQR
Sciurus_carolin   GSSVFCKASLELQEDVQEMHAVRKEVAQTSVNPSVISVKTDGEDPNELLKNFQDIMRKQR
Sciurus_niger@A   GSSVFCKASLELQEDVQEMHAVRKEVAQTSVNPNVISVKTDGEDPNELLKNFQDIMRKQR
                  **.** *******************.**:.* *.::.***:*.**. *********:***

Alas1             PERVSHLLQDNLPKSVSTFQYDHFFEKKIDEKKNDHTYRVFKTVNRRAQIFPMADDYTDS
Aplodontia_rufa   PERVSHLLQDNLPKSVSTFQYDHFFEKKIDEKKNDHTYRVFKTVNRRAQIFPMADDYSDS
Xerus_inauris@A   PERVSHLLQDNLPKSVSTFQYDHFFEKKIDEKKNDHTYRVFKTVNRRAQIFPMADDYSDS
```

```
Marmota_monax@A PERVSHLLQDNLPKSVSTFQYDHFFEKKIDEKKNDHTYRVFKTVNRRAQIFPMADDYSDS
Spermophilus_da PERVSHLFQDNLPKSVSTFQYDHFFEKKIDEKKNDHTYRVFKTVNRRAQIFPMADDYSDS
Ictidomys_tride PERVSHLLQDNLPKSVSTFQYDHFFEKKIDEKKNDHTYRVFKTVNRRAQIFPMADDYSDS
Urocitellus_par PERVSHLLQDNLPKSVSTFQYDHFFEKKIDEKKNDHTYRVFKTVNRRAQIFPMADDYSDS
Sciurus_vulgari PERVSHLLQDNLPKSVSTFQYDHFFEKKIDEKKNDHTYRVFKTVNRRAQIFPMADDYSDS
Sciurus_carolin PERVSHLLQDNLPKSVSTFQYDHFFEKKIDEKKNDHTYRVFKTVNRRAQIFPMADDYSDS
Sciurus_niger@A PERVSHLLQDNLPKSVSTFQYDHFFEKKIDEKKNDHTYRVFKTVNRRAQIFPMADDYSDS
                *******:*************************************************:**

Alas1           LITKKQVSVWCSNDYLGMSRHPRVCGAVMETVKQHGAGAGGTRNISGTSKFHVELEQALA
Aplodontia_rufa LITKKQVSVWCSNDYLGMSRHPRVCGAVMETVKQHGAGAGGTRNISGTSKFHVELEQELA
Xerus_inauris@A LITKKQVSVWCSNDYLGMSRHPRVCGAVMETVKQHGAGAGGTRNISGTSKFHVELEQELA
Marmota_monax@A LITKKQVSVWCSNDYLGMSRHPRVCGAVMETVKQHGAGAGGTRNISGTSKFHVELEQELA
Spermophilus_da LITKKQVSVWCSNDYLGMSRHPRVCGAVVETLKQHGAGAGGTRNISGTSKFHVELEQELA
Ictidomys_tride LITKKQVSVWCSNDYLGMSRHPRVCGAVMETVKQHGAGAGGTRNISGTSKFHVELEQELA
Urocitellus_par LITKKQVSVWCSNDYLGMSRHPRVCGAVMETVKQHGAGAGGTRNISGTSKFHVELEQELA
Sciurus_vulgari LITKKQVSVWCSNDYLGMSRHPRVCGAVMETVKQHGAGAGGTRNISGTSKFHVELERALA
Sciurus_carolin LITKKQVSVWCSNDYLGMSRHPRVCGAVMETVKQHGAGAGGTRNISGTSKFHVELEQELA
Sciurus_niger@A LITKKQVSVWCSNDYLGMSRHPRVCGAVMETVKQHGAGAGGTRNISGTSKFHVELERELA
                ****************************:**:************************: **

Alas1           DLHGKDAALLFSSCFVANDSTLFTLAKMMPGCEIYSDSGNHASMIQGIRNSRVPKYIFRH
Aplodontia_rufa DLHGKDAALLFSSCFVANDSTLFTLAKMMPGCEIYSDSGNHASMIQGIRNSRVPKYIFRH
Xerus_inauris@A DLHGKDAALLFSSCFVANDSTLFTLAKMMPGCEIYSDSGNHASMIQGIRNSRVPKYIFRH
Marmota_monax@A DLHGKDAALLFSSCFVANDSTLFTLAKMMPGCEIYSDSGNHASMIQGIRNSRVPKYIFRH
Spermophilus_da DLHGKDAALLFSSCFVANDSTLFTLAKMMPGCEIYSDSGNHASMIQGIRNSRVPKYVFRH
Ictidomys_tride DLHGKDAALLFSSCFVANDSTLFTLAKMMPGCEIYSDSGNHASMIQGIRNSRVPKYIFRH
Urocitellus_par DLHGKDAALLFSSCFVANDSTLFTLAKMMPGCEIYSDSGNHASMIQGIRNSRVPKYIFRH
Sciurus_vulgari DLHGKDAALLFSSCFVANDSTLFTLAKMMPGCEIYSDSGNHASMIQGIRNSRVPKYIFRH
Sciurus_carolin DLHGKDAALLFSSCFVANDSTLFTLAKMMPGCEIYSDSGNHASMIQGIRNSRVPKYIFRH
Sciurus_niger@A DLHGKDAALLFSSCFVANDSTLFTLAKMMPGCEIYSDSGNHASMIQGIRNSRVPKYIFRH
                ********************************************************:***

Alas1           NDVNHLRELLQRSDPSVPKIVAFETVHSMDGAVCPLEELCDVAHEFGAITFVDEVHAVGL
Aplodontia_rufa NDVNHLRELLQRSDPSVPKIVAFETVHSMDGAVCPLEELCDVAHEFGAITFVDEVHAVGL
Xerus_inauris@A NDVNHLRELLQRSDPSVPKIVAFETVHSMDGAVCPLEELCDVAHEFGAITFVDEVHAVGL
Marmota_monax@A NDVNHLRELLQRSDPSVPKIVAFETVHSMDGAVCPLEELCDVAHEFGAITFVDEVHAVGL
Spermophilus_da NDVNHLRELLQRSDPSVPKIVAFETVHSMDGAVCPLEELCDVAHEFGAITFVDEVHAVGL
Ictidomys_tride NDVNHLRELLQRSDPSVPKIVAFETVHSMDGAVCPLEELCDVAHEFGAITFVDEVHAVGL
Urocitellus_par NDVNHLRELLQRSDPSVPKIVAFETVHSMDGAVCPLEELCDVAHEFGAITFVDEVHAVGL
Sciurus_vulgari NDVNHLRELLQRSDPSVPKIVAFETVHSMDGAVCPLEELCDVAHEFGAITFVDEVHAVGL
Sciurus_carolin NDVNHLRELLQRSDPSVPKIVAFETVHSMDGAVCPLEELCDVAHEFGAITFVDEVHAVGL
Sciurus_niger@A NDVNHLRELLQRSDPSVPKIVAFETVHSMDGAVCPLEELCDVAHEFGAITFVDEVHAVGL
                ************************************************************

Alas1           YGARGGGIGDRDGVMPKMDIISGTLGKAFGCVGGYIASTSLLIDTVRSYAAGFIFTTSLP
Aplodontia_rufa YGARGGGIGDRDGVMPKMDIISGTLGKAFGCVGGYIASTSLLVDTVRSYAAGFIFTTSLP
Xerus_inauris@A YGAQGGGIGDRDGVMPKMDIISGTLGKAFGCVGGYIASTSLLIDTVRSYAAGFIFTTSLP
Marmota_monax@A YGARGGGIGDRDGVMPKMDIISGTLGKAFGCVGGYIASTSLLIDTVRSYAAGFIFTTSLP
Spermophilus_da YGARGGGIGDRDGVMPKMDIISGTLGKAFGCVGGYIASTSLLIDTVRSYAAGFIFTTSLP
Ictidomys_tride YGARGGGIGDRDGVMPKMDIISGTLGKAFGCVGGYIASTSLLIDTVRSYAAGFIFTTSLP
Urocitellus_par YGARGGGIGDRDGVMPKMDIISGTLGKAFGCVGGYIASTSLLIDTVRSYAAGFIFTTSLP
Sciurus_vulgari YGAQGGGIGDRDGVMPKMDIISGTLGKAFGCVGGYIASTSLLIDTVRSYAAGFIFTTSLP
Sciurus_carolin YGAQGGGIGDRDGVMPKMDIISGTLGKAFGCVGGYIASTSLLIDTVRSYAAGFIFTTSLP
Sciurus_niger@A YGAQGGGIGDRDGVMPKMDIISGTLGKAFGCVGGYIASTSLLIDTVRSYAAGFIFTTSLP
                ***:**************************************:*****************
```

```
Alas1              PMLLAGALESVRILKSSEGRALRRQHQRNVKLLRQMLMDAGLPVIHCPSHIIPVRVADAA
Aplodontia_rufa    PMLLAGALESVRILKSSEGRALRRQHQRNVKLMRQMLMDAGLPVIHCPSHIIPVRVADAA
Xerus_inauris@A    PMLLAGALESVRILKSSEGRALRRQHQRNVKLMRQMLMDAGLPVIHCPSHIIPVRVADAA
Marmota_monax@A    PMLLAGALESVRILKSSEGRALRRQHQRNVKLMRQMLMDAGLPVIHCPSHIIPVRVADAA
Spermophilus_da    PMLLAGALESVRILKSSEGRALRRQHQRNVKLMRQMLMDAGLPVIHCPSHIIPVRVADAA
Ictidomys_tride    PMLLAGALESVRILKSSEGRALRRQHQRNVKLMRQMLMDAGLPVIHCPSHIIPVRVADAA
Urocitellus_par    PMLLAGALESVRILKSSEGRALRRQHQRNVKLMRQMLMDAGLPVIHCPSHIIPVRVADAA
Sciurus_vulgari    PMLLAGALESVRILKSSEGRALRRQHQRNVKLMRQMLMDAGLPVIHCPSHIIPVRVADAA
Sciurus_carolin    PMLLAGALESVRILKSSEGRALRRQHQRNVKLMRQMLMDAGLPVIHCPSHIIPVRVADAA
Sciurus_niger@A    PMLLAGALESVRILKSSEGRALRRQHQRNVKLMRQMLMDAGLPVIHCPSHIIPVRVADAA
                   ********************************:***************************

Alas1              KNTEICDELMTRHNIYVQAINYPTVPRGEELLRIAPTPHHTPQMMNFFVEKLLVTWKRVG
Aplodontia_rufa    KNTEICDELMSRHNIYVQAINYPTVPRGEELLRIAPTPHHTPQMMNFFVEKLLVTWKRVG
Xerus_inauris@A    KNTEICDELMSRHNIYVQAINYPTVPRGEELLRIAPTPHHTPQMMNFFVEKLLVTWKRVG
Marmota_monax@A    KNTEICDELMSRHNIYVQAINYPTVPRGEELLRIAPTPHHTPQMMNFFVEKLLVTWKRVG
Spermophilus_da    KNTEICDELMSRHNIYVQAINYPTVPRGEELLRIAPTPHHTPQMMNFFVEKLLATWKRVG
Ictidomys_tride    KNTEICDELMSRHNIYVQAINYPTVPRGEELLRIAPTPHHTPQMMNFFVEKLLVTWKRVG
Urocitellus_par    KNTEICDELMSRHNIYVQAINYPTVPRGEELLRIAPTPHHTPQMMNFFVEKLLVTWKRVG
Sciurus_vulgari    KNTEICDELMSRHNIYVQAINYPTVPRGEELLRIAPTPHHTPQMMNFFVEKLLATWKRVG
Sciurus_carolin    KNTEICDELMSRHNIYVQAINYPTVPRGEELLRIAPTPHHTPQMMNFFVEKLLATWKRLG
Sciurus_niger@A    KNTEICDELMSRHNIYVQAINYPTVPRGEELLRIAPTPHHTPQMMNFFVEKLLATWKRVG
                   **********:******************************************.****:*

Alas1              LELKPHSSAECNFCRRPLHFEVMSEREKAYFSGMSKMVSAQAZ
Aplodontia_rufa    LELKPHSSAECNFCRRPLHFEVMSEREKSYFSGMSKLVSAQA-
Xerus_inauris@A    LELKPHSSAECNFCRRPLHFEVMSEREKAYFSGMSKLVSAQA-
Marmota_monax@A    LELKPHSSAECNFCRRPLHFEVMSEREKSYFSGLSKLVSAQA-
Spermophilus_da    LELKPHSSAECNFCRRPLHFEVMSEREKSYFSGMSKLVSAQA-
Ictidomys_tride    LELKPHSSAECNFCRRPLHFEVMSEREKAYFSGMSKLVSAQA-
Urocitellus_par    LELKPHSSAECNFCRRPLHFEVMSEREKSYFSGMSKLVSAQA-
Sciurus_vulgari    LELKPHSSAECNFCRRPLHFEVMSEREKAYFSGMSKLVSAQA-
Sciurus_carolin    LELKPHSSAECNFCRRPLHFEVMSEREKAYFSGMSKLVSAQA-
Sciurus_niger@A    LELKPHSSAECNFCRRPLHFEVMSEREKAYFSGMSKLVSAQA-
                   ****************************:****:**:*****
```

ALAS2 encodes 5'-aminolevulinate synthase 2. Diseases associated with *ALAS2* include anemia, sideroblastic, 1 and protoporphyria, erythropoietic, X-linked pyridoxine-responsive sideroblastic anemia. Among its related pathways are porphyrin and glycine, serine, and threonine metabolism. Gene ontology (GO) annotations related to this gene include pyridoxal phosphate binding and glycine binding. An important paralog of this gene is *ALAS1*. The product of this gene specifies an erythroid-specific mitochondrially located enzyme. The encoded protein catalyzes the first step in the heme biosynthetic pathway. Alternatively spliced transcript variants encoding different isoforms have been identified.

Among 586 amino acids, we observed three differences among *Sciurus* species at *ALAS2*. We noted an insertion of 15-16 amino acids at positions 61-76 in seven non-*Sciurus* sequences. *X. inauris* had an insertion of five amino acids at positions 554-559.

```
Alas2              MVAAAMLLRSCPVLSQGPTGLLGKVAKTYQFLFSIGRCPILATQGPTCSQIHLKATKAGG
Aplodontia_rufa    MVAAAVLLQCCPVLARGPTGLLGKVIKTHQFLFGTGRCPILAAQGPICSQIHLKATKAGG
Xerus_inauris@A    MVAAAMLLQCCPVLARGPTGLLGKVIKTHQFLFGTGRCPILATQGPICSQIHLKTTNAGG
Marmota_monax@A    MVAAAVLLQCCPVLARGPTGLLSKVVKTHQFLFGTGRCPILATQGPICSQIHLKATRAGG
```

```
Spermophilus_da MVAAAVLLQCCPVLARGPTGLLSKVVKTHQFLFGTGRCPILATQGPICSQIHLKATRAGG
Ictidomys_tride MVAAAVLLQCCPVLARGPTGLLSKVIKTHQFLFGTGRCPILATQGPICSQIHLKATRAGG
Urocitellus_par MVAAAVLLQCCPVLARGPTGLLSKVVKTHQFLFGTGRCPILATQGPICSQIHLKATRAGG
Sciurus_vulgari MVAAAVLLQCCPVLARGPTGLLGKVVKTHQFLFGTGRCPILAAQGPICSQIHLKATKAGG
Sciurus_carolin MVAAAVLLQCCPVLARGPTGLLGKVVKTHQFLFGTGRCPILATQGPICSQIHLKATKAGG
Sciurus_niger@A MVAAAVLLQCCPVLVRGPTVLLGKVVKTHQFLFGTGRCPILATQGPICSQIHLKATKAGG
                *****:**:.**** :*** **.** **:****. *******:*** *******:*.***

Alas2           ----------------DSPSWAKSHCPFMLSELQDRKSKIVQRAAPEVQEDVKTFKTDLL
Aplodontia_rufa ----------------DSPSWAKGHCPFMLSELQDGKSKIVQKAAPEVQEDVKTFKTDLL
Xerus_inauris@A ----------------DSPSWTKGHCPFMLSELQDGKSKIVQKAAPEVQEDVKTFKTDLL
Marmota_monax@A GKKRFLEKGGCLGHSDDSPSWAKSHCPFMLSELQDRKSKIVQKAAPEVQEDVKTFKTDLL
Spermophilus_da GKKRFLEKGGCLSHS-DSPSWAKSHCPFMLSELQDRKSKIVQKAAPEVQEDVKTFKTDLL
Ictidomys_tride GKKRFLEKGGCLGHS-DSPSWAKSHCPFMLSELQDRKSKIVQKAAPEVQEDVKTFKTDLL
Urocitellus_par GKKRFLEKGGCLGHS-DSPSWAKSHCPFMLSELQDRKSKIVQKAAPEVQEDVKTFKTDLL
Sciurus_vulgari ----------------DSPSWSKGHCPFMLSELQDGKSKIVQKAAPEVQEDVKTFKTDLL
Sciurus_carolin ----------------DSPSWSKGHCPFMLSELQDGKSKIVQKAAPEVQEDVKTFKTDLL
Sciurus_niger@A ----------------DSPSWSKGHCPFMLSELQDGKSKIVQKAAPEVQEDVKTFKTDLL
                                *****:*.*********** ******:*****************

Alas2           STMDSTTRSHSFPSFQEPEQTEGAVPHLIQNNMTGSQAFGYDQFFRDKIMEKKQDHTYRV
Aplodontia_rufa STMDSTTRSHSFPSFQEPEQTEGAVPHLIQNNMTGEHAFGYDQFFRDKIMEKKQDHTYRV
Xerus_inauris@A STMDSTTRSHSFPSFQEPEQTEGAVPHLIQNNMTGEHAFGYDQFFRDKIMEKKQDHTYRV
Marmota_monax@A STMDSTTRSHSFPSFQEPEQTEGAVPHLIQNNMTGEHAFGYDQFFRDKIMEKKQDHTYRV
Spermophilus_da STMDSTTRSHSFPSFQEPEQTEGAVPHLIQNNMTGEHAFGYDQFFRDKIMEKKQDHTYRV
Ictidomys_tride STMDSTTRSHSFPSFQEPEQTEGAVPHLIQNNMTGEHAFGYDQFFRDKIMEKKQDHTYRV
Urocitellus_par STMDSTTRSHSFPSFQEPEQTEGAVPHLIQNNMTGEHAFGYDQFFRDKIMEKKQDHTYRV
Sciurus_vulgari STMDSTTRSHSFPSFQEPEQTEGAVPHLIQSNPAGEHAFGYDQFFRDKIMEKKQDHTYRV
Sciurus_carolin STMDSTTRSHSFPSFQEPEQTEGAVPHLIQSNPAGEHAFGYDQFFRDKIMEKKQDHTYRV
Sciurus_niger@A STMDSTTRSHSFPSFQEPEQTEGAVPHLIQSNPAGEHAFGYDQFFRDKIMEKKQDHTYRV
                ******************************.* :*.:***********************

Alas2           FKTVNRWANAYPFAQHFSEASMASKDVSVWCSNDYLGISRHPRVLQAIEETLKNHGAGAG
Aplodontia_rufa FKTVNRWANAYPFAQHFSEASMASKDVSVWCSNDYLGMSRHPRVLQAIQETLKNHGAGAG
Xerus_inauris@A FKTVNRWANAYPFAQHFSEASMASKDVSVWCSNDYLGMSRHPRVLQAIQETLKNHGAGAG
Marmota_monax@A FKTVNRWANAYPFAQHFSEASMASKDVSVWCSNDYLGISRHPRVLQAIEETLKNHGAGAG
Spermophilus_da FKTVNRWANAYPFAQHFSEASMASKDVSVWCSNDYLGISRHPRVLQAIEETLKNHGAGAG
Ictidomys_tride FKTVNRWANAYPFAQHFSEASMASKDVSVWCSNDYLGMSRHPRVLQAIQETLKNHGAGAG
Urocitellus_par FKTVNRWANAYPFAQHFSEASMASKDVSVWCSNDYLGMSRHPRVLQAIQETLKNHGAGAG
Sciurus_vulgari FKTVNRWANAYPFAQHFSEASMASKDVSVWCSNDYLGISRHPRVLQAIEETLKNHGAGAG
Sciurus_carolin FKTVNRWANAYPFAQHFSEASMASKDVSVWCSNDYLGMSRHPRVLQAIQETLKNHGAGAG
Sciurus_niger@A FKTVNRWANAYPFAQHFSEASMASKDVSVWCSNDYLGMSRHPRVLQAIQETLKNHGAGAG
                *************************************:**********:***********

Alas2           GTRNISGTSKFHVELEQELAELHQKDSALLFSSCFVANDSTLFTLAKLLPGCEIYSDAGN
Aplodontia_rufa GTRNISGTSKFHVELEQELAELHQKDSALLFSSCFVANDSTLFTLAKLLPGCEIYSDAGN
Xerus_inauris@A GTRNISGTSKFHVELEQELAELHQKDSALLFSSCFVANDSTLFTLAKLLPGCEIYSDAGN
Marmota_monax@A GTRNISGTSKFHVELEQELAELHQKDSALLFSSCFVANDSTLFTLAKLLPGCEIYSDAGN
Spermophilus_da GTRNISGTSKFHVELEQELAELHQKDSALLFSSCFVANDSTLFTLAKLLPGCEIYSDAGN
Ictidomys_tride GTRNISGTSKFHVELEQELAELHQKDSALLFSSCFVANDSTLFTLAKLLPGCEIYSDAGN
Urocitellus_par GTRNISGTSKFHVELEQELAELHQKDSALLFSSCFVANDSTLFTLAKLLPGCEIYSDAGN
Sciurus_vulgari GTRNISGTSKFHVELEQELAELHQKDSALLFSSCFVANDSTLFTLAKLLPGCEIYSDAGN
Sciurus_carolin GTRNISGTSKFHVELEQELAELHQKDSALLFSSCFVANDSTLFTLAKLLPGCEIYSDAGN
Sciurus_niger@A GTRNISGTSKFHVELERELAELHQKDSALLFSSCFVANDSTLFTLAKLLPGCEIYSDAGN
                ****************:*******************************************
```

```
Alas2              HASMIQGIRNSGAAKFVFRHNDPGHLKKLLEKSDPKTPKIVAFETVHSMDGAICPLEELC
Aplodontia_rufa    HASMIQGIRNSGAAKFVFRHNDPGHLKKLLEKSDPKTPKIVAFETVHSMDGAICPLEELC
Xerus_inauris@A    HASMIQGIRNSGAAKFVFRHNDPGHLKKLLEKSDPKTPKIVAFETVHSMDGAICPLEELC
Marmota_monax@A    HASMIQGIRNSGAAKFVFRHNDPGHLKKLLEKSDPKTPKIVAFETVHSMDGAICPLEELC
Spermophilus_da    HASMIQGIRNSGAAKFVFRHNDPGHLKKLLEKSDPKTPKIVAFETVHSMDGAICPLEELC
Ictidomys_tride    HASMIQGIRNSGAAKFVFRHNDPGHLKKLLEKSDPKTPKIVAFETVHSMDGAICPLEELC
Urocitellus_par    HASMIQGIRNSGAAKFVFRHNDPGHLKKLLEKSDPKTPKIVAFETVHSMDGAICPLEELC
Sciurus_vulgari    HASMIQGIRNSGAAKFVFRHNDPGHLKKLLEKSDPKTPKIVAFETVHSMDGAICPLEELC
Sciurus_carolin    HASMIQGIRNSGAAKFVFRHNDPGHLKKLLEKSDPKTPKIVAFETVHSMDGAICPLEELC
Sciurus_niger@A    HASMIQGIRNSGAAKFVFRHNDPGHLKKLLEKSDPKTPKIVAFETVHSMDGAICPLEELC
                   ************************************************************

Alas2              DVAHQYGALTFVDEVHAVGLYGARGAGIGERDGIMHKLDIISGTLGKAFGCVGGYIASTR
Aplodontia_rufa    DVAHQYGALTFVDEVHAVGLYGARGAGIGERDGIMHKLDIISGTLGKAFGCVGGYIASTR
Xerus_inauris@A    DVAHQYGALTFVDEVHAVGLYGARGAGIGERDGIMHKLDIISGTLGKAFGCVGGYIASTR
Marmota_monax@A    DVAHQYGALTFVDEVHAVGLYGARGAGIGERDGIMHKLDIISGTLGKAFGCVGGYIASTR
Spermophilus_da    DVAHQYGALTFVDEVHAVGLYGARGAGIGERDGIMHKLDIISGTLGKAFGCVGGYIASTR
Ictidomys_tride    DVAHQYGALTFVDEVHAVGLYGARGAGIGERDGIMHKLDIISGTLGKAFGCVGGYIASTR
Urocitellus_par    DVAHQYGALTFVDEVHAVGLYGARGAGIGERDGIMHKLDIISGTLGKAFGCVGGYIASTR
Sciurus_vulgari    DVAHQYGALTFVDEVHAVGLYGARGAGIGERDGIMHKLDIISGTLGKAFGCVGGYIASTR
Sciurus_carolin    DVAHQYGALTFVDEVHAVGLYGARGAGIGERDGIMHKLDIISGTLGKAFGCVGGYIASTR
Sciurus_niger@A    DVAHQYGALTFVDEVHAVGLYGARGAGIGERDGIMHKLDIISGTLGKAFGCVGGYIASTR
                   ************************************************************

Alas2              DLVDMVRSYAAGFIFTTSLPPMVLSGALESVRLLKGEEGQALRRAHQRNVKHMRQLLMDR
Aplodontia_rufa    DLVDMVRSYAAGFIFTTSLPPMVLSGALESVRLLKGEEGQALRRAHQRNVKHMRQLLMDR
Xerus_inauris@A    DLVDMVRSYAAGFIFTTSLPPMVLSGALESVRLLKGEEGQALRRAHQRNVKHMRQLLMDR
Marmota_monax@A    DLVDMVRSYAAGFIFTTSLPPMVLSGALESVRLLKGEEGQALRRAHQRNVKHMRQLLMDR
Spermophilus_da    DLVDMVRSYAAGFIFTTSLPPMVLSGALESVRLLKGEEGQALRRAHQRNVKHMRQLLMDR
Ictidomys_tride    DLVDMVRSYAAGFIFTTSLPPMVLSGALESVRLLKGEEGQALRRAHQRNVKHMRQLLMDR
Urocitellus_par    DLVDMVRSYAAGFIFTTSLPPMVLSGALESVRLLKGEEGQALRRAHQRNVKHMRQLLMDR
Sciurus_vulgari    DLVDMVRSYAAGFIFTTSLPPMVLSGALESVRLLKGEEGQALRRAHQRNVKHMRQLLMDR
Sciurus_carolin    DLVDMVRSYAAGFIFTTSLPPMVLSGALESVRLLKGEEGQALRRAHQRNVKHMRQLLMDR
Sciurus_niger@A    DLVDMVRSYAAGFIFTTSLPPMVLSGALESVRLLKGEEGQALRRAHQRNVKHMRQLLMDR
                   ************************************************************

Alas2              GFPVIPCPSHIIPIRVGNAALNSKICDLLLSKHSIYVQAINYPTVPRGEELLRLAPSPHH
Aplodontia_rufa    GLPVIPCPSHIIPIRVGNAALNSKICDLLLSKHSIYVQAINYPTVPRGEELLRLAPSPHH
Xerus_inauris@A    GLPVIPCPSHIIPIRVGDAALNSKICDLLLSKHSIYVQAINYPTVPRGEELLRLAPSPHH
Marmota_monax@A    GFPVIPCPSHIIPIRVGDAALNSKICDLLLSKHSIYVQAINYPTVPRGEELLRLAPSPHH
Spermophilus_da    GLPVIPCPSHIIPIRVGDAALNSKICDLLLSKHSIYVQAINYPTVPRGEELLRLAPSPHH
Ictidomys_tride    GFPVIPCPSHIIPIRVGDAALNSKICDLLLSKHSIYVQAINYPTVPRGEELLRLAPSPHH
Urocitellus_par    GLPVIPCPSHIIPIRVGDAALNSKICDLLLSKHSIYVQAINYPTVPRGEELLRLAPSPHH
Sciurus_vulgari    GLPVIPCPSHIIPIRVGDAALNSKICDLLLSKHSIYVQAINYPTVPRGEELLRLAPSPHH
Sciurus_carolin    GFPVIPCPSHIIPIRVGDAALNSKICDLLLSKHSIYVQAINYPTVPRGEELLRLAPSPHH
Sciurus_niger@A    GLPVIPCPSHIIPIRVGDAALNSKICDLLLSKHSIYVQAINYPTVPRGEELLRLAPSPHH
                   *:***************:******************************************

Alas2              SPQMMENFVEKLL-----LAWTEVGLPLQDVSVAACNFCHRPVHFELMSEWERSYFGNMG
Aplodontia_rufa    SPQMMENFVEKLL-----LAWTEVGLPLQDVSVAACNFCHRPVHFELMSEWERSYFGNMG
Xerus_inauris@A    SPQMMENFVGKYLQSCGSFPGTCIKLSLQCLSL---------------------------
Marmota_monax@A    SPQMMENFVEKLL-----LAWTEVGLPLQDVSVAACNFCHRPVHFELMSEWERSYFGNMG
Spermophilus_da    SPQMMENFVEKLL-----LAWTEVGLPLQDVSVAACNFCHRPVHFELMSEWERSYFGNMG
Ictidomys_tride    SPQMMENFVEKLL-----LAWTEVGLPLQDVSVAACNFCHRPVHFELMSEWERSYFGNMG
Urocitellus_par    SPQMMENFVEKLL-----LAWTEVGLPLQDVSVAACNFCHRPVHFELMSEWERSYFGNMG
Sciurus_vulgari    SPQMMENFVEKLL-----VAWTEVGLPLQDVSVAACNFCHRPVHFELMSEWERSYFGNMG
```

```
Sciurus_carolin SPQMMENFVEKLL-----VAWTEVGLPLQDVSVAACNFCHRPVHFELMSEWERSYFGNMG
Sciurus_niger@A SPQMMENFVEKLL-----VAWTEVGLPLQDVSVAACNFCHRPVHFELMSEWERSYFGNMG
                ********* * *     .. * : *.** :*:

Alas2           PQYVTTYAZ
Aplodontia_rufa PQYVTTYA-
Xerus_inauris@A ---------
Marmota_monax@A PQYVTTYA-
Spermophilus_da PQYVTTYA-
Ictidomys_tride PQYVTTYA-
Urocitellus_par PQYVTTYA-
Sciurus_vulgari PQYVTTYA-
Sciurus_carolin PQYVTTYA-
Sciurus_niger@A PQYVTTYA-
```

CPOX encodes coproporphyrinogen oxidase. Diseases associated with *CPOX* include hereditary coproporphyria and harderoporphyria. Among its related pathways are porphyrin metabolism and biosynthesis of cofactors. Gene ontology (GO) annotations related to this gene include protein homodimerization activity and structural constituent of eye lens. An important paralog of this gene is *ENSG00000285635*. The protein encoded by this gene is the sixth enzyme of the heme biosynthetic pathway. The encoded enzyme is soluble and found in the intermembrane space of mitochondria. This enzyme catalyzes the stepwise oxidative decarboxylation of coproporphyrinogen III to protoporphyrinogen IX, a precursor of heme.

Within a 426-amino acid *CPOX* sequence, we noted eight point-differences among sciurids. All members of *Sciurus* showed addition of a leucine at position 17. The human sequence had an insertion of two amino acids at positions 115-116.

```
Cpox            MALRLGRLGSDPWWRA-VLGDYAQLRAASPRCASARVCQLPGTAGPQPRRGLGYG-----
Aplodontia_rufa MAWHLGRLSAGSCLRA-ARGGCGELRAWSQRCAGGRICRPPGTAGTESCRGLAHG-----
Xerus_inauris@C MAWHLGRLSVGSCWRA-ARGGCGELRAWSQRCAAGRMCRPPGTTGTEHSRGLAQG-----
Marmota_monax@C MAWYLGRLSAGPCWRA-ARGGCGELRAWSQRCATGRICQPPGTAGTMHSRGLAHG-----
Spermophilus_da MAWYLGRLSAGPCWRA-ARGGCGELRAWSQRCATGRICQPPGTAGTGHSRGLLEGXXXXX
Ictidomys_tride MAWYLGRLSAGPCWRA-ARGGCGELRAWSQRCATGHICQPPGTAGTVHSRGLAHG-----
Urocitellus_par MAWYLGRLSAGPCWRA-ARGGCGELRAWSQRCATGRICQPPGTAGTVHSRGLAHG-----
Sciurus_vulgari MAWHLGRLSAGPCWRALARGGCGELRAWSQRCAAGRICRPPGTAGTEHSRGLAHG-----
Sciurus_carolin MAGHLGRLSAGPCWRALARGGCGELRAWSQRCAAGRICRPPGTAGTEHSRGLAHG-----
Sciurus_niger@C MAWHLGRLSAGPCWRALARGGCGELRAWSQRCAAGRICRPPGTAGTEHSRGLAHG-----
                **  ****. ..  ** . *. .:*** * *** .::*: ***:*.   ***  *

Cpox            -PWARGGSGLGTRLAATLAGLAGLAAAAFGHVQRAEMVPKSSGARSPSPGRREEDGDELA
Aplodontia_rufa -PSARGSRWPGTGLTAALAGLAGLATAAVGHVHRAEMVPKSSGARSPSPERPLE--DELA
Xerus_inauris@C -PSAKGGPWPGTGLAAALAGLAGLATAAFGHVQRAEMVPKSSGARSSSQERPLD--DDLA
Marmota_monax@C -PSARGGPWPGTGLAAALAGLAGLATAAFGHVQRAEMVPKSSGARSPSPERPQE--DDLA
Spermophilus_da XXXXXXXXXXXXXXXXXXXXXXXXXXXXXXXXXXAEMVPKSSGARSPSPVRPQE--DDLA
Ictidomys_tride -PSARGGPWPGTGLAAALAGLAGLATAAFGHVHRAEMVPKSSGARSPSPERPQE--DDLA
Urocitellus_par -PSARGGPWPGTGLAAALAGLAGLATAAFGHVHRAEMVPKSSGARSPSPERPQE--DDLA
Sciurus_vulgari -PSSGGGSWPGTGLATALAGLAGLATAAFGHVQRAEMVPKSSGARSPSPERSQE--DDLT
Sciurus_carolin -PSSGGGSRPGAGLATALAGLAGLATAAFGHVQRAEMVPKSSGVRSPSPERPQE--DDLT
Sciurus_niger@C -PSSGGGSRPGAGLATALAGLAGLATAAFGHVQRAEMVPKSSGVRSPSPERPQE--DDLT
                                                  *********.**.*  *  :  *:*:

Cpox            RRCSTFMSSPVTELRELRRRPEDMKTKMELMIMETQAQVCRALAQVDGVADFTVDRWERK
```

```
Aplodontia_rufa RRCSCFMASPVTDLGELRRRPGDMKTKMELLILETQAQVCQALAQVDGGANFSVDRWERK
Xerus_inauris@C RRCSCFMASPVTDLSELRKRPGDMKTKMELLILETQAQVCQALAQVDGGASFSVDRWERK
Marmota_monax@C RRCSCFMASPVTDLDELRKRPGDMKTKMELLILETQAQVCQALAQVDGVASFSVDRWERK
Spermophilus_da RRCSCFMASPVTDLDELRKRPGDMKTKMELLILETQAQVCQALAQVDGVASFSVDRWERK
Ictidomys_tride RRCSCFMASPVTDLDELRKRPGDMKTKMELLILETQAQVCQALAQVDGVASFSVDRWERK
Urocitellus_par RRCSCFMASPVTDLDELRKRPGDMKTKMELLILETQAQVCQALAQVDGVASFSVDRWERK
Sciurus_vulgari RRCSCFMALPVTDLGELRKRPGDMKTKMELLILETQAQVCQALAQVDGGASFSVDRWERK
Sciurus_carolin RRCSCFMALPVTDLGELRKRPGDMKTKMELLILETQAQVCQALAQVDGGASFSVDRWERK
Sciurus_niger@C RRCSCFMALPVTDLGELRKRPGDMKTKMELLILETQAQVCQALAQVDGGASFSVDRWERK
                **** **: ***:* ***:** ********:*:*******:******* *.*:*******

Cpox            EGGGGITCVLQDGRVFEKAGVSISVVHGNLSEEAANQMRGRGKTLKTKDSKLPFTAMGVS
Aplodontia_rufa EGGGGISCVLQDGRVFEKAGVSISVVHGNLSEEAAKQMRSRGKILKTKDGKLPFTAMGVS
Xerus_inauris@C EGGGGISCVLQDGRVFEKAGVSISVVHGNLSEEAAKQMRSRGKVLKTNDGKLPFSAMGVS
Marmota_monax@C EGGGGISCVLQDGRVFEKAGVSISVVHGNLSEEAAKQMRSRGKVLKTKDGKLPFCAMGVS
Spermophilus_da EGGGGISCVLQDGRVFEKAGVSISVVHGNLSEEAAKQMRSRGKVLKTKDGKLPFSAMGVS
Ictidomys_tride EGGGGISCVLQDGRVFEKAGVSISVVHGNLSEEAAKQMRSRGKVLKTKDGKLPFSAMGVS
Urocitellus_par EGGGGISCVLQDGRVFEKAGVSISVVHGNLSEEAAKQMRSRGKVLKTKDGKLPFSAMGVS
Sciurus_vulgari EGGGGITCVLQDGRVFEKAGVSISVVHGNLSEEAAKQMRSRGKVLKTKDGKLPFSAMGVS
Sciurus_carolin EGGGGISCVLQDGRVFEKAGVSISVVHGNLSEEAAKQMRSRGKVLKTKDGKLPFSAMGVS
Sciurus_niger@C EGGGGISCVLQDGRVFEKAGVSISVVHGNLSEEAAKQMRSRGKVLKTKDGKLPFSAMGVS
                ******:****************************:***.*** ***:*.**** *****

Cpox            SVIHPKNPYAPTMHFNYRYFEVEEADGNTHWWFGGGCDLTPTYLNQEDAVHFHRTLKEAC
Aplodontia_rufa SVIHPKNPYAPTMHFNYRYFEVEEADGTKQWWFGGGCDLTPTYLNQEDAVHFHRTLKEAC
Xerus_inauris@C SVIHPKNPHAPTIHFNYRYFEVEEADGTKQWWFGGGCDLTPTYLNQEDAVHFHRTLKEAC
Marmota_monax@C SVIHPKNPHAPTIHFNYRYFEVEEADGTKQWWFGGGCDLTPTYLNQEDAVHFHRTLKEAC
Spermophilus_da SVIHPKNPHAPTIHFNYRYFEVEEADGTKQWWFGGGCDLTPTYLNQEDAVHFHRTLKEAC
Ictidomys_tride SVIHPKNPHAPTIHFNYRYFEVEEADGTKQWWFGGGCDLTPTYLNQEDAVHFHRTLKEAC
Urocitellus_par SVIHPKNPHAPTIHFNYRYFEVEEADGTKQWWFGGGCDLTPTYLNQEDAVHFHRTLKEAC
Sciurus_vulgari SVIHPKNPYAPTMHFNYRYFEVEEADGNTQWWFGGGCDLTPTYLNQEDAVHFHRTLKEAC
Sciurus_carolin SVIHPKNPYAPTIHFNYRYFEVEEADGNKQWWFGGGCDLTPTYLNQEDAVHFHRTLKEAC
Sciurus_niger@C SVIHPKNPYAPTIHFNYRYFEVEEADGNKQWWFGGGCDLTPTYLNQEDAVHFHRTLKEAC
                ********:***:**************..:******************************

Cpox            DQHGPDIYPKFKKWCDDYFFIVHRGERRGIGGIFFDDLDSPSKEEAFRFVKTCAEAVVPS
Aplodontia_rufa DQHGPDLYPKFKKWCDDYFFIVHRGERRGIGGIFFDDLDSPSKEEVFRFVKSCAQAVVPS
Xerus_inauris@C DQHGPDLYPKFKKWCDDYFFITHRGERRGIGGIFFDDLDSPSKEEVFHFVQSCARAVVPS
Marmota_monax@C DQHGPDLYPKFKKWCDDYFFIAHRGERRGIGGIFFDDLDSPSKEEVFRFVQSCAQAVVPS
Spermophilus_da DQHGPDLYPKFKKWCDDYFFIAHRGERRGIGGIFFDDLDSPSKEEVFRFVQSCAQAVVPS
Ictidomys_tride DQHGPDLYPKFKKWCDDYFFIAHRGERRGIGGIFFDDLDSPSKEEVFRFVQSCAQAVVPS
Urocitellus_par DQHGPDLYPKFKKWCDDYFFIAHRGERRGIGGIFFDDLDSPSKEEVFRFVQSCAQAVVPS
Sciurus_vulgari DQHGPDLYPKFKKWCDDYFFITHRGERRGIGGIFFDDLDSPSKEEAFRFVKTCAEAVVPS
Sciurus_carolin DQHGPDIYPKFKKWCDDYFFITHRGERRGIGGIFFDDLDSPSKEEAFRFVKTCAEAVVPS
Sciurus_niger@C DQHGPDLYPKFKKWCDDYFFITHRGERRGIGGIFFDDLDSPSKEEAFRFVKTCAEAVVPS
                ******:**************.***********************.*:**::**.*****

Cpox            YVPIVKKHCDDSYTPRDKLWQQLRRGRYVEFNLLYDRGTKFGLFTPGSRIESILMSLPLT
Aplodontia_rufa YIPLVKKHCDDSFTPQEKLWQQLRRGRYVEFNLLYDRGTKFGLFTPGSRIESILMSLPLT
Xerus_inauris@C YIPLVKKHCDDSFTPQEKLWQQLRRGRYVEFNLLYDRGTKFGLFTPGSRIESILMSLPLT
Marmota_monax@C YIPLVKKHCDDSFTPQEKLWQQLRRGRYVEFNLLYDRGTKFGLFTPGSRIESILMSLPLT
Spermophilus_da YIPLVKKHCDDSYTPREKLWQQLRRGRYVEFNLLYDRGTKFGLFTPGSRIESILMSLPLT
Ictidomys_tride YIPLVKKHCDDSFTPQEKLWQQLRRGRYVEFNLLYDRGTKFGLFTPGSRIESILMSLPLT
Urocitellus_par YIPLVKKHCDDSFTPQEKLWQQLRRGRYVEFNLLYDRGTKFGLFTPGSRIESILMSLPLT
Sciurus_vulgari YVPIVKKHCDDSYTPRDKLWQQLRRGRYVEFNLLYDRGTKFGLFTPGSRIESILMSLPLT
Sciurus_carolin YVPLVKKHCDDSYTPQEKLWQQLRRGRYVEFNLLYDRGTKFGLFTPGSRIESILMSLPLT
```

```
Sciurus_niger@C YVPLVKKHCDDSYTPQEKLWQQLRRGRYVEFNLLYDRGTKFGLFTPGSRIESILMSLPLT
                *:*:********:**::*******************************************

Cpox            ARWEYMHSPPENSKEAEILEVLRHPKDWVHZ
Aplodontia_rufa AR-----------------------------
Xerus_inauris@C AR-----------------------------
Marmota_monax@C AR-----------------------------
Spermophilus_da AR-----------------------------
Ictidomys_tride AR-----------------------------
Urocitellus_par AR-----------------------------
Sciurus_vulgari ARWEYMHSPPENSKAKT--QSLHPPRDWVH-
Sciurus_carolin ARWAYMHLPPENSKEAEILRVLHHPRDWVH-
Sciurus_niger@C ARWAYMHSPPENSK-----------------
                **
```

FECH encodes ferrochelatase. Diseases associated with *FECH* include erythropoietic protoporphyria 1 and autosomal erythropoietic protoporphyria. Among its related pathways are porphyrin metabolism and the HIF-1-alpha transcription factor network. Gene ontology (GO) annotations related to this gene include iron ion binding and 2 iron, 2 sulfur cluster binding. The protein encoded by this gene is localized to the mitochondrion, where it catalyzes the insertion of the ferrous form of iron into protoporphyrin IX in the heme synthesis pathway. Two transcript variants encoding different isoforms have been found for this gene.

Among 319 amino acids for FECH, sciurids differed at three. *X. inauris* had a 10-amino acid deletion at positions 256-265, and M. monax a 15-amino acid deletion at positions 250-264.

```
Fech            MLSASANMAAALRAAGALLREPLVHGSSRACQPWRCQ-SGAAVAATTEKVHHAKTTKPQA
Aplodontia_rufa ------------------------------------------------------------
Xerus_inauris@F ----------------------LVCGNLRASRSWKCKLNTIAVAVDKGKDQCVKNANPQV
Marmota_monax@F ----------------------LVLGNSKASQSWKCKLNTTAVAVHTGKVQFVKNKNPQV
Spermophilus_da ----------------------LVLGNSKASQSWKCKLNTTAVAVHTGKVQFVKNKNLQV
Ictidomys_tride ------------------------------------------------------------
Urocitellus_par ----------------------LVLGNSKASQSWKCKLNTTAVAVHTGKVQFVKNKNPQV
Sciurus_vulgari ------------------------------------------------------------
Sciurus_carolin ------------------------------------------------------------
Sciurus_niger@F ------------------------------------------------------------


Fech            QPERRKPKTGILMLNMGGPETLGEVQDFLQRLFLDRDLMTLPIQNKLAPFIAKRRTPKIQ
Aplodontia_rufa --------------------------------------------SKLAPFIAKRRTPKIQ
Xerus_inauris@F QLEKKKPKTGI-MLNIGGPETLGEVQDFLQRLFLDRDLIIFPI-NKLAPFIAKRRTPKIQ
Marmota_monax@F QLEKKNPKTGILMLNIGGPETLGEVQDFLQRLFLDRDLMIFPI-NKLAPFIAKRRTPKIQ
Spermophilus_da QLE-KNPKTGILMLNIGGPETLGEVQDFLQRLFLDRDLMIFPI-NKLAPFIAKRRTPKIQ
Ictidomys_tride --------------------------------------------SKLAPFIAKRRTPKIQ
Urocitellus_par QLEKKNPKTGILMLNIGGPETLGEVQDFLQRLFLDRDLMIFPI-NKLAPFIAKRRTPKIQ
Sciurus_vulgari --------------------------------------------SKLAPFIAKRRTPRIQ
Sciurus_carolin --------------------------------------------SKLAPFIAKRRTPRIQ
Sciurus_niger@F --------------------------------------------SKLAPFIAKRRTPRIQ
                                                            .************:**

Fech            EQYRRIGGGSPIKMWTSKQGEGMVKLLDELSPATAPHKYYIGFRYVHPLTEEAIEEMERD
Aplodontia_rufa EQYRRIGGGSPIKMWTSKQGEGMVKLLDDLSPDTAPHKYYIGFRYVHPLTEEAIDKMERD
Xerus_inauris@F EQYRRIGGGSPIKMWTSKQGEGMVKLLDELSPATAPHKYYIGFRYVHPLTEEAIEEMERD
Marmota_monax@F EQYRRIGGGSPIKMWTSKQGEGMVKLLDELSPATAPHKYYIGFRYVHPLTEEAIEEMERD
Spermophilus_da EQYRRIGGGSPIKMWTSKQGEGMVKLLDELSPATAPHKYYIGFRYVHPLTEEAIEEMERD
```

```
Ictidomys_tride EQYRRIGGGSPIKMWTSKQGEGMVKLLDELSPNTAPHKYYIGFRYVHPLTEEAIEEMERD
Urocitellus_par EQYRRIGGGSPIKMWTSKQGEGMVKLLDELSPATAPHKYYIGFRYVHPLTEEAIEEMERD
Sciurus_vulgari EQYRRIGGGSPIKMWTSKQGEGMVKLLDELSPTTAPHKYYIGFRYVHPLTEEAIDEMERD
Sciurus_carolin EQYRRIGGGSPIKMWTSKQGEGMVKLLDELSPTTAPHKYYIGFRYVHPLTEEAIDEMERD
Sciurus_niger@F EQYRRIGGGSPIKMWTSKQGEGMVKLLDELSPTTAPHKYYIGFRYVHPLTEEAIDEMERD
                ****************************:*** *********************::****

Fech            GLERAIAFTQYPQYSCSTTGSSLNAIYRYYNEVGQKPTMKWSTIDRWPTHPLLIQCFADH
Aplodontia_rufa GLERAIAFTQYPQYSCSTTGSSLNAIYRYYNEVGKKPTMKWSTIDRWPTHPLLIQVSQDH
Xerus_inauris@F GLERAIAFTQYPQYSCSTTGSSLNAIYRYYNEVGQKPTMKWSTIDRWPTHPLLIQCFADN
Marmota_monax@F GLERAIAFTQYPQYSCSTTGSSLNAIYRYYNEVGQKPTMKWSTIDRWPTHPLLIQCFADY
Spermophilus_da GLERAIAFTQYPQYSCSTTGSSLNAIYRYYNEVGQKPTMKWSTIDRWPTHPLLIQCFADH
Ictidomys_tride GLERAIAFTQYPQYSCSTTGSSLNAIYRYYNEVGQKPTMKWSTIDRWPTHPLLIQCFADH
Urocitellus_par GLERAIAFTQYPQYSCSTTGSSLNAIYRYYNEVGQKPTMKWSTIDRWPTHPLLIQCFADH
Sciurus_vulgari GLERAIAFTQYPQYSCSTTGSSLNAIYRYYNEVGKKPTMKWSTIDRWPTHPLLIQVSQDH
Sciurus_carolin GLERAIAFTQYPQYSCSTTGSSLNAIYRYYNEVGKKPTMKWSTIDRWPTHPLLIQVSQDH
Sciurus_niger@F GLERAIAFTQYPQYSCSTTGSSLNAIYRYYNEVGKKPTMKWSTIDRWPTHPLLIQVSQDH
                **********************************:********************   * 

Fech            ILKELNHFPEEKRSEVVILFSAHSLPMSVVNRGDPYPQEVGATVHKVMEKLGYPNPYRLV
Aplodontia_rufa ILKELNHFPEEKRSEVVILFSAHSLPLQVVNRGDPYPQEVGATVHKVMESLGYSNPYRLV
Xerus_inauris@F LQKELHHFPLEKGGH----------QMSVVNRGDPYPQEVGATVHKVMEKLGYPNPYRLV
Marmota_monax@F LQKELHHFP---------------LPMSVVNRGDPYPQEVGATVHKVMERLGYSNPYRLV
Spermophilus_da ILKELNHFPEEKRSEVVILFSAHSLPMSVVNRGDPYPQEVGATVHKVMERLGYSNPYRLV
Ictidomys_tride ILKELNHFPEEKRSEVVILFSAHSLPMSVVNRGDPYPQEVGATVHKVMERLGYSNPYRLV
Urocitellus_par ILKELNHFPEEKRSEVVILFSAHSLPMSVVNRGDPYPQEVGATVHKVMERLGYSNPYRLV
Sciurus_vulgari ILKELNHFPEEKRSEVVILFSAHSLPMQVVNRGDPYPQEVGATVHKVMERLGYSNPYRLV
Sciurus_carolin ILKELNHFPEEKRSEVVILFSAHSLPMQVVNRGDPYPQEVGATVHKVMERLGYSNPYRLV
Sciurus_niger@F ILKELNHFPEEKRSEVVILFSAHSLPMQVVNRGDPYPQEVGATVHKVMERLGYSNPYRLV
                : ***:***                 :.********************* ***.******

Fech            WQSKVGPVPWLGPQTDEAIKGLCERGRKNILLVPIAFTSDHIETLYELDIEYSQVLAQKC
Aplodontia_rufa WQSKVGPVSWLGPQTDEAIKGLCERGRKNILLVPIAFTSDHIETLYELDIEYSQILAKEC
Xerus_inauris@F WQSKVGPVPWLGPQTDEAIKGLCERGRKNILLVPIAFTSDHIETLYELDIEYSQVLAQKC
Marmota_monax@F WQSKVGPVPWLGPQTDEAIKGLCERGRKNILLVPIAFTSDHIETLYELDIEYSQVLAQKY
Spermophilus_da WQSKVGPVPWLGPQTDEAIKGLCERGRKNILLVPIAFTSDHIETLYELDIEYSQVLAKEV
Ictidomys_tride WQSKVGPVPWLGPQTDEAIKGLCERGRKNILLVPIAFTSDHIETLYELDIEYSQVLAKEV
Urocitellus_par WQSKVGPVPWLGPQTDEAIKGLCERGRKNILLVPIAFTSDHIETLYELDIEYSQVLAKEC
Sciurus_vulgari WQSKVGPVPWLGPQTNEAIKGLCERGRKNILLVPIAFTSDHIETLYELDIEYSQVLAKEV
Sciurus_carolin WQSKVGPVPWLGPQTNEAIKGLCERGRKNILLVPIAFTSDHIETLYELDIEYSQVLAKEC
Sciurus_niger@F WQSKVGPVPWLGPQTNEAIKGLCERGRKNILLVPIAFTSDHIETLYELDIEYSQVLAKEC
                ********.******:**************************************:**:: 

Fech            GAENIRRAESLNGNPLFSKALADLVHSHIQSNKLCSTQLSLNCPLCVNPVCRKTKSFFTS
Aplodontia_rufa GAENIRRAESLNGNPLFSQALADLVHSHIQSNRLCSKQLTLSCPLCVNPVCRETKSFFTN
Xerus_inauris@F GAENIMKRESFAGNPLFSQALADLVYSHIQSNKLCSKQLTLSCPLCVNPVCRETKSFFTN
Marmota_monax@F -VENNKRGESLNGNPLFSQALADLVLSHIQSNKLCSKQLTLSCPLCVNPVCRETKSFFTN
Spermophilus_da GAENIRRAESLNGNPLFSQALADLVLSHIQSNKLCSKQLTLSCPLCVNPVCRETKSFFTN
Ictidomys_tride GAENIRRAESLNGNPLFSQALADLVLSHIQSNKLCSKQLTLSCPLCVNPVCRETKSFFTN
Urocitellus_par GAENIRRAESLNGNPLFSQALADLVLSHIQSNKLCSKQLTLSCPLCVNPVCRETKSFFTN
Sciurus_vulgari STENIRRAESLNGNPLFSQALADLVHSHIQSNTQCSKQLTLSCPLCVNPVCRETKSFFTN
Sciurus_carolin GAENIRRAESLNGNPLFSQALADLVHSHIQSNTLCSKQLTLSCPLCVNPVCRETKSFFTN
Sciurus_niger@F GAENIRRAESLNGNPLFSQALADLVHSHIQSNTLCSKQLTLSCPLCVNPVCRETKSFFTN
                 .**  : **: ******:****** ******  **.**:*.**********:******.

Fech            QQLZ
```

```
Aplodontia_rufa QQL-
Xerus_inauris@F QQL-
Marmota_monax@F QQL-
Spermophilus_da QQL-
Ictidomys_tride QQL-
Urocitellus_par QQL-
Sciurus_vulgari QQM-
Sciurus_carolin QQM-
Sciurus_niger@F QQM-
                **:
```

HMBS encodes hydroxymethylbilane synthase. Diseases associated with *HMBS* include acute intermittent porphyria and mood disorder. Among its related pathways are porphyrin metabolism and biosynthesis of cofactors. Gene ontology (GO) annotations related to this gene include hydroxymethylbilane synthase activity. The encoded protein is the third enzyme of the heme biosynthetic pathway and catalyzes the head-to-tail condensation of four porphobilinogen molecules into the linear hydroxymethylbilane. Alternatively spliced transcript variants encoding different isoforms have been described.

Among 242 amino acids in *Sciurus niger* HMBS, there were two point-differences among *Sciurus* species and an insertion of 37 amino acids in *S. vulgaris*. There were major amino acid sequence insertions among all species, including 37 amino acids in *A. rufa*; five amino acids for *M. monax, I. dauricus,* and *U. parryi*; and eight amino acids for *S. dauricus*.

```
Hmbs            MSGNGGAATTAEENGSKMRVIRVGTRKSQLARIQTDTVVAMLKALYPGIQFEIIAMSTTG
Aplodontia_rufa ------------------------------------------------------------
Xerus_inauris@H ------------------------------------------------------------
Marmota_monax@H ------------------------------------------------------------
Spermophilus_da ------------------------------------------------------------
Ictidomys_tride ------------------------------------------------------------
Urocitellus_par ------------------------------------------------------------
Sciurus_vulgari ------------------------------------------------------------
Sciurus_carolin ------------------------------------------------------------
Sciurus_niger@H ------------------------------------------------------------


Hmbs            DKILDTALSKIGEKSLFTKELENALEKNEVDLVVHSLKDVPTILPPGFTIGAICKRENPC
Aplodontia_rufa ------------------------------------------ICPSFHDLPSFPRRENPY
Xerus_inauris@H ------------------------------------------------------RRENPC
Marmota_monax@H ------------------------------------------------------RRENPC
Spermophilus_da ------------------------------------------------------RRENPC
Ictidomys_tride ------------------------------------------------------RRENPC
Urocitellus_par ------------------------------------------------------RRENPC
Sciurus_vulgari ------------------------------------------------------------
Sciurus_carolin ------------------------------------------------------RRENPC
Sciurus_niger@H ------------------------------------------------------RRENPC


Hmbs            DAVVFHPKFIGKTLETLPEK-------------------------------------SAV
Aplodontia_rufa DAVVFHPKFVGKTLETLPEKRVGPEWAAWWDVHRRWKAGRSRKGTVTICISFTFSLSSTV
Xerus_inauris@H DAVVFHPKFVGKTLETLPEK-------------------------------------SAV
Marmota_monax@H DAVVFHPKFVGKTLETLPEK-------------------------------------SAV
Spermophilus_da DAVVFHPKFVGKTLETLPEK-------------------------------------SAV
```

```
Ictidomys_tride DAVVFHPKFVGKTLETLPEK-------------------------------------SAV
Urocitellus_par DAVVFHPKFVGKTLETLPEK-------------------------------------SAV
Sciurus_vulgari ------------------------------------------------------------
Sciurus_carolin DAVVFHPKFVGKTLETLPEK-------------------------------------SAV
Sciurus_niger@H DAVVFHPKFVGKTLETLPEK-------------------------------------SAV


Hmbs            GTSSLRRVAQLQRKFPHLEFKSI-----RGNLNTRLRKLDELQEFSAIVLAVAGLQRMGW
Aplodontia_rufa GTSSLRRAAQLQRKFPHLEFKNI-----RGNLNTRLRKLDEQHEFSAIILAVAGLQRMGW
Xerus_inauris@H GTSSLRRAAQLQRKFPHLEFKSI-----RGNLNTRLRKLDEQHEFSAIILAVAGLQRMGW
Marmota_monax@H GTSSLRRAAQLQRKFPHLEFKSIVSFRRRGNLNTRLRKLDEQHEFSAIILAVAGLQRMGW
Spermophilus_da GTSSLRRAAQLQRKFPHLEFKSIVSFRRRGNLNTRLRKLDEQHEFSAIILAVAGLQRMGW
Ictidomys_tride GTSSLRRAAQLQRKFPHLEFKSI-----RGNLNTRLRKLDEQHEFSAIILAVAGLQRMGW
Urocitellus_par GTSSLRRAAQLQRKFPHLEFKSIVSFRRRGNLNTRLRKLDEQHEFSAIILAVAGLQRMGW
Sciurus_vulgari ----------------------------RGNLNTRLRKLDEQHEFSAIILAVAGLQRMGW
Sciurus_carolin GTSSLRRAAQLQRKFPHLEFKSI-----RGNLNTRLRKLDEQHEFSAIILAVAGLQRMGW
Sciurus_niger@H GTSSLRRAAQLQRKFPHLEFKSI-----RGNLNTRLRKLDEQHEFSAITLAVAGLQRMGW
                                            ************* :***** ***********

Hmbs            QNRVGQI-----------LHPEECMYAVGQGALAVEVRAKDQDILDLVSVLHDPETLLRC
Aplodontia_rufa QNRVGQVGASPLPSPIYLLLPPPCFFSVGQGALGVEVRAKDQDILDLVGVLHDPETLLRC
Xerus_inauris@H QNRVGQV-----------GAPNECMYAVGQGALGVEVRAKDQDILDLVGVLHDPETLLRC
Marmota_monax@H QNRVGQV-----------LHPEECMYAVGQGALAVEVRAKDQDILDLVGVLHDPETLLRC
Spermophilus_da QNRVGQV-----------LHPEECMYAVGQGALAVEVRAKDQDILDLVGVLHDPETLLRC
Ictidomys_tride QNRVGQI-----------VERRSSLMCFHQGALAVEVRAKDQDILDLVGVLHDPETLLRC
Urocitellus_par QNRVGQI-----------LERRSSLMCFHQGALAVEVRAKDQDILDLVGVLHDPETLLRC
Sciurus_vulgari QNRVGQV-----------LHPEECMYAVGQGALGVEVRAKDQDILDLVGVLHDPETLLRC
Sciurus_carolin QNRVGQV-----------LHPEECMYAVGQGALGVEVRAKDQDILDLVGVLHDPETLLRC
Sciurus_niger@H QNRVGQV-----------LHPEECMYAVGQGALGVEVRAKDQDILDLVGVLHDPETLLRC
                ******:                .: .. ****.**************.***********

Hmbs            IAERAFLRHL--------EGGCSVPVAVHTVMKDG-------------------------
Aplodontia_rufa IAERAFLRHL--------EGGCSVPVAVHTVMKDG-------------------------
Xerus_inauris@H IAERAFLRHL--------EGGCSVPVAVHTVMKDG-------------------------
Marmota_monax@H IAERAFLRHL--------EGGCSVPVAVHTVIKDG-------------------------
Spermophilus_da IAERAFLRHL--------EGGCSVPVAVHTVIKDG-------------------------
Ictidomys_tride IAERAFLRHLVGPVLVLEEGGCSVPVAVHTVIKDG-------------------------
Urocitellus_par IAERAFLRHL--------EGGCSVPVAVHTVIKDG-------------------------
Sciurus_vulgari IAERAFLRHL--------EGGCSVPVAVHTVMKDGQVSSGKWAGRQRTGISPYASQVSKQ
Sciurus_carolin IAERAFLRHL--------VGGCSVPVAVHTVMKDG-------------------------
Sciurus_niger@H IAERAFLRHL--------VGGCSVPVAVHTVMKDG-------------------------
                **********         ************:***

Hmbs            ------------QLYLTGGVWSLDGSDSMQETMQATIQVPVQQEDGPEDDPQLVGITARN
Aplodontia_rufa ------------QLYLTGGVWSLDGSDSMQETMQATIHVPVQHEDGPEDDPQLVGITARN
Xerus_inauris@H ------------QLYLTGGVWSLDGSDSMQETMQATINVPVQYEDGPEDDPQLVGITARN
Marmota_monax@H ------------QLYLTGGVWSLDGSDSMQETMQATIHVPVQYDHGPEDDPQLVGITARN
Spermophilus_da ------------QLYLTGGVWSLDGSDSMQETMQATIHVPVQYDDGPEDDPQLVGITALN
Ictidomys_tride ------------QLYLTGGVWSLDGSDSMQETMQATIHVPTQQYDGPEDDPQLVGITALN
Urocitellus_par ------------QLYLTGGVWSLDGSESMQETMQATIHVPVQYDNGPEDDPQLVGITALN
Sciurus_vulgari SPLRICNNYFLSQLYLTGGVWSLDGSDSMQETMQATIHVPVQYEDGPEDDPQLVGITARN
Sciurus_carolin ------------QLYLTGGVWSLDGSDSMQETMQATIQVPIQYEDGPEDDPQLVGITARN
Sciurus_niger@H ------------QLYLTGGVWSLDGSDSMQETMQATIQVPIQYEDGPEDDPQLVGITARN
                            **************:**********:** *  .************* *

Hmbs            IPRGAQLAAENLGISLASLLLNKGAKNILDVARQLNDVRZ
```

```
Aplodontia_rufa IPRGAQLAAENLGISLANLLLNKGAKNILDVARQLNDVH-
Xerus_inauris@H IPRGAQLAAENLGISLANLLLNKGAKNILDVARQLNDV--
Marmota_monax@H IPRGAQLAAENLGISLASLLLNKGAKNILDVARQLNDVH-
Spermophilus_da IPRGAQLAAENLGISLASLLLNKGAKNILDVARQLNDVH-
Ictidomys_tride IPRGAQLAAENLGISLASLLLNKGAKNILDVARQLNDVH-
Urocitellus_par IPRGAQLAAENLGISLASLLLNKGAKNILDVARQVNDVH-
Sciurus_vulgari IPRGAQLAAENLGISLANLLLNKGAKNILDVARQLNDV--
Sciurus_carolin IPRGAQLAAENLGISLANLLLNKGAKSILDVARQLNDV--
Sciurus_niger@H IPRGAQLAAENLGISLANLLLNKGAKSILDVARQLNDV--
                *****************.********.*******:***
```

PPOX encodes protoporphyrinogen oxidase. Among its related pathways are porphyrin and biosynthesis of cofactors. Gene ontology (GO) annotations related to this gene include oxidoreductase activity and oxygen-dependent protoporphyrinogen oxidase activity. This gene encodes the penultimate enzyme of heme biosynthesis, which catalyzes the 6-electron oxidation of protoporphyrinogen IX to form protoporphyrin IX. Mutations in this gene cause variegate porphyria, an autosomal dominant disorder of heme metabolism resulting from a deficiency in protoporphyrinogen oxidase, an enzyme located on the inner mitochondrial membrane. Alternatively spliced transcript variants encoding the same protein have been identified.

Relative to a 557-amino acid sequence for *S. niger*, we observed four point-differences among sciurids. There were insertions of 31 amino acids for *M. monax* and *X. inauris*, 25 for *S. niger*, 24 for *M. monax* and *I. tridecemlineatus*, and 25 for *X. inauris*. There was a deletion of 32 amino acids for *A. rufa;* of 35 for *A. rufa*, *X. inauris*, and *I. tridecemlineatus*; of eight amino acids in *A. rufa*, *X. inauris*, and *I. tridecemlineatus*; and of 26 amino acids for *M. monax* and *S. dauricus*.

```
Ppox            MGRTVIVLGGGISGLAASYHLIRGPSPPKVILVEGSKRLGGWIRSIRGSDGAIFELGPRG
Aplodontia_rufa -----------------------SPPCRQVVLVEGSKRLGGWIRSVRGPDGAIFELGPRG
Xerus_inauris@P ------------------------PSCRQVVLVEGSKRLGGWIRSVRGPDGAIFELGPRG
Marmota_monax@P ----------------------------QVVLVEGSKRLGGWIRSIRGPGGAIFELGPRG
Spermophilus_da ----------------------------QVVLVEGSKRLGGWIRSIRGPGGAIFELGPRG
Ictidomys_tride ----------------------------QVVLVEGSKRLGGWIRSIRGPGGAIFELGPRG
Urocitellus_par ----------------------------QVVLVEGSKRLGGWIRSIRGPGGAIFELGPRG
Sciurus_vulgari ------------------------PSCRQVVLVEGSKRLGGWIRSVRGPDGAIFELGPRG
Sciurus_carolin ---------------AASDGTTMAPSCRQVVLVEGSKRLGGWIRSVRGPDGAIFELGPRG
Sciurus_niger@P ------------------------PSCRQVVLVEGSKRLGGWIRSVRGPDGAIFELGPRG
                                            :*:**************:**..**********

Ppox            IRPAGALGARTLLLVSELGLESEVLPVRGDHPAAQNRFLYVGGTLHPLPSGLRGLLRPSP
Aplodontia_rufa IRPAGALGARTLLLVSELGLESEVLPVRGDHPAAQNRFLYVGGTLHPLPSGLRGLLRPSP
Xerus_inauris@P IRPAGPLGARTLLLVSELGLESEVLPVRGDHPAAQNRFLYVGGTLHPLPSGLRGLLRPSP
Marmota_monax@P IRPAGVLGARTLLLVSELGLESEVLPVRGDHPAAQNRFLYVGGTLHPLPSGLRGLLRPSP
Spermophilus_da IRPAGVLGARTLLLVSELGLESEVLPVRGDHPAAQNRFLYVGGTLHPLPSGLRGLLRPSP
Ictidomys_tride IRPAGVLGARTLLLVSELGLESEVLPVRGDHPAAQNRFLYVGGTLHPLPSGLRGLLRPSP
Urocitellus_par IRPAGVLGARTLLLVSELGLESEVLPVRGDHPAAQNRFLYVGGTLHPLPSGLRGLLRPSP
Sciurus_vulgari IRPAGALGARTLLLVSELGLESEVLPVRGDHPAAQNRFLYVGGTLHPLPSGLRGLLRPSP
Sciurus_carolin IRPAGALGARTLLLVSELGLESEVLPVRGDHPAAQNRFLYVGGTLHPLPSGLRGLLRPSP
Sciurus_niger@P IRPAGALGARTLLLVSELGLESEVLPVRGDHPAAQNRFLYVGGTLHPLPSGLRGLLRPSP
                ***** ******************************************************

Ppox            PFSKPLFWAGLRELLKPRGKEPDETVHSFAQRRLGPEVASLAMDSLCRGVFAGNSRELSI
Aplodontia_rufa PFSKSLLWTGLRELTKPRGKEPDETVHSFAQRRLGPEVASLVMDSLCRGVFAGNSRELSV
```

```
Xerus_inauris@P PFSKPLLWAGLRELTKPRGKEPDETVHSFAQRRLGPEVASLAMDSLCRGVFAGNSRELSI
Marmota_monax@P PFSKPLLWAGLRELTKPRGKEPDETVHSFAQRRLGPEVASLAMDSICRGVFAGNSRELSI
Spermophilus_da PFSKPLLWAGLRELTKPRGKEPDETVHSFAQRRLGPEVASLAMDSLCRGVFAGNSRELSI
Ictidomys_tride PFSKPLLWAGLRELTKPRGKEPDETVHSFAHRRLGPEVASLVMDSLCRGVFAGNSRELSI
Urocitellus_par PFSKPLLWAGLRELTKPRGKEPDETVHSFAQRRLGPEVASLVMDSLCRGVFAGNSRELSI
Sciurus_vulgari PFSRPLLWAGLRELTKPRSKEPDETVHSFAQRRLGPEVASLAMDSLCRGVFAGNSRELSI
Sciurus_carolin PFSRPLLWAGLRELTKPRSKEPDETVHSFAQRRLGPEVASLAMDSLCRGVFAGNSRELSI
Sciurus_niger@P PFSRPLLWAGLRELTKPRSKEPDETVHSFAQRRLGPEVASLAMDSLCRGVFAGNSRELSI
                ***:.*:*:***** ***.***********:**********.***:*************:

Ppox            RSCFPSLFQAEQTHRSILLGLLLG-------------------------------AGQSP
Aplodontia_rufa RSCFPSLFQAEQTHRSILLGLLLG-------------------------------AGQSP
Xerus_inauris@P RSCFPSLFQAEQTYRSIILGLLLGAGEEGLVQRVKILRTARVRQGVLVRFFFAVSLGQSP
Marmota_monax@P RSCFPSLFQAEQTHRSVILGLLFS-------------------------------SGQSP
Spermophilus_da RSCFPSLFQAEQTHRSVILGLLFS-------------------------------SGKSP
Ictidomys_tride RSCFPSLFQAEQTHRSVILGLLFS-------------------------------SGKSP
Urocitellus_par RSCFPSLFQAEQTHRSVILGLLFS-------------------------------SGKSP
Sciurus_vulgari RSCFPSLFQAEQTHRSIILGLLLG-------------------------------AGQSP
Sciurus_carolin RSCFPSLFQAEQTHRSIILGLLLG-------------------------------AGQSP
Sciurus_niger@P RSCFPSLFQAEQTHRSIILGLLLGAGE------VAGRYGLPVRQGVLIFFFAAASAGQSP
                *************:**::****:.                                *:**

Ppox            QPDSSLIRQARAERWSQWSLRGGLEVLPQALHNHLASKGVTVLSGQPVCGLSLQPEGRWK
Aplodontia_rufa QPDSALVRQARAERWSQWSLRGGMEMLPQTLHTYLTSRGVSVLKGQPVCGLTLQAEGHWK
Xerus_inauris@P QSDSALIRQAQAERWSQWSLRGGMEMLPQTLDAYLTSRGVSVLRGQPVCGLTLQAEGRWK
Marmota_monax@P QPDSALIRQARAERWSQWSLRGGMETLPQTLDTYLTSRGVSVLRGQPVCGLSLQAEGHWK
Spermophilus_da QPDSALIRQARAERWSQWSLRGGMETLPQTLDTYLTSRGVSVLRGQPVCGLSLQAEGHWK
Ictidomys_tride QPDSALIRQARAERWSQWSLRGGMETLPQTLDTYLTSRGVSILRGQPVCGLSLQAEGHWK
Urocitellus_par QPDSALIRQARAERWSQWSLRGGMETLPQTLDTYLTSRGVSVLRGQPVCGLSLQAEGHWK
Sciurus_vulgari QPDSALIRQARAERWSQWSLRGGMEMLPQTLDTYLTSNGVSVLRGQPVCGLTLQAEGHWK
Sciurus_carolin QPDSALIRQARAERWSQWSLRGGMEMLPQTLDTYLTSNGVSVLRGQPVSGLTLQAEGHWK
Sciurus_niger@P QPDSALIRQARAERWSQWSLRGGMEMLPQTLDTYLTSNGVSVLRGQPVSGLTLQAEGHWK
                *.**:*:***:************:* ***:*. :*:*.**::* ****.**:**.**:**

Ppox            VSLGDSSLEADHIISAIPASELSKLLPAEAAPLARILSTIKAVSVAVVNLQYRGACLPV-
Aplodontia_rufa VSLGDSSLEADHIISAIPASELSKLLPAEAAPLAHILSTITAVSVAVVNLQYRGARLPV-
Xerus_inauris@P VSLGDSSLEADHIISAIPASELSKLLPMEAAPLAQVLSTITAVSVAVVNLQYQGARLPVQ
Marmota_monax@P VSLGDSSLEADHIISAIPASELSKLLPTEAAPLAQVLNTITAVSVAVVNLQYRGARLPVQ
Spermophilus_da VSLGDSSLEADHIISAIPASELSKLLPTEAAPLAQVLNTITAVSVAVVNLQYRGARLPV-
Ictidomys_tride VSLGDSSLEADHIISAIPASELSKLLPTEAAPLAQVLNNITAVSVAVVNLQYRGACLPVQ
Urocitellus_par VSLGDSSLEADHIISAIPASELSKLLPTEAAPLAQVLNTITAVSVAVVNLQYRGARLPV-
Sciurus_vulgari VSLGDSSLEADHIISAIPASELSKLLPAEAAPLARILSTIKAVSVAVVNLQYRGACLPL-
Sciurus_carolin VSLGDSSLEADHIISAIPASELSKLLPAEAAPLARILSTIKAVSVAVVNLQYRGACLPL-
Sciurus_niger@P VSLGDSSLEADHIISAIPASELSKLLPAEAAPLARILSTIKAVSVAVVNLQYRGACLPL-
                *************************** ******::*..*.***********:** **:

Ppox            ------------------------QGFGHLVPSSEDPTVLGIVYDSVAFPEQDGNPPSLR
Aplodontia_rufa ------------------------QGFGHLVPSSEDPGVLGIVYDSVAFPEQDGSSPGLR
Xerus_inauris@P VYWRGKEGIDSDGTFPSFFFFFPLQGFGHLVPSSEDPGVLGIVYDSVAFPEQDGSPPGLR
Marmota_monax@P VYRGEKKVPQMEFFLLI-FFFFPLQGFGHLVPSSEDPGVLGIVYDSVAFPEQDGSPPGLR
Spermophilus_da ------------------------QGFGHLVPSSEDPGVLGIVYDSVAFPEQDGSPPGLR
Ictidomys_tride VYGGGKKGVASDGVFPS-YFFFSLQGFGHLVPSSEDPGVLGIVYDSVAFPEQDGSSPGLR
Urocitellus_par ------------------------QGFGHLVPSSEDPGVLGIVYDSVAFPEQDGSPPGLR
Sciurus_vulgari ------------------------QGFGHLVPSSEDPGVLGIVYDSVAFPEQDGSPPGLR
Sciurus_carolin ------------------------QGFGHLVPSSEDPGVLGIVYDSVAFPEQDGNPPGLR
Sciurus_niger@P ------------------------QGFGHLVPSSEDPGVLGIVYDSVAFPEQDGNPPGLR
```

```
                                        ************* ****************..*.**

Ppox            VT--------------------------------VMLGGYWLQKLKAAGHQLSPELFQQQ
Aplodontia_rufa VTVRA--------------------------------GGYWLQKLKAAGHQLSPELFQQQ
Xerus_inauris@P VTVRAGHVVWAFQRALLCPVVGKAKLVSAIF-APVMLGGSWLQMLEASGCVLSQELFQQQ
Marmota_monax@P VTVRAGHVVWAFQRALLCSIVGKAKLVSAIFPSQVMLGGSWLQTLEASGCVLSQELFQKQ
Spermophilus_da VTVRAGHFVWAFQRALFCPIVGKAKLVSAIFPSQVMLGGSWLQTLEASGYVLSRELFQQQ
Ictidomys_tride VTVRAGHVVWAFQRTLFCPIVGKAKLVSAIFPSQVMLGGSWLQTLEASGCVLSQELFQQQ
Urocitellus_par VTVRAGHVVWAFQRALFCPIVGKAKLVSPIFPSQVMLGGSWLQTLEASGCVLSQELFQQQ
Sciurus_vulgari VTVRAGHVVRVFQRAPLCPIVGKTKLVSAIFPSQVMLGGSWLQMLEASGCVLSQKLFQQQ
Sciurus_carolin VTVRAGHVVRVFQRAPLCPIVGKAKLVSAIFPPQVMLGGSWLQMLEASDCVLSQKLFQQQ
Sciurus_niger@P VTVRAGHVVRVFQRAPLCPTVGKAKLVSAIFPPQVMLGGSWLQMLEASGCVLSQKLFQQQ
                **                                   ** *** *:*:.  ** :***:*

Ppox            AQEAAATQLGLKEPPSHCLVHLHK------------------------------------
Aplodontia_rufa AQEAAATQLGLKEPPSHCLVHLHQ------------------------------------
Xerus_inauris@P AQEAVATQLGLKEPPSHCLVHLHK------------------------------------
Marmota_monax@P AQEAVATQLGLKEPPSHCLVHLHKVSWGKFLSVPCCRPCIQGIFVTVCQPRPGKSIINFI
Spermophilus_da AQEAVATQLGLKEPPSHCLVHLHKVSWGKFLSVPYCRPCIQGIFVTVCQPRPGKSIINFI
Ictidomys_tride AQEAVATQLGLKEPPSHCLVHLHK------------------------------------
Urocitellus_par AQEAVATQLGLKEPPSHCLVHLHKVSWGKFLSVPYCRPCIQGIFVTVCQPRPGKSIINFI
Sciurus_vulgari AQEAVATQLGLKEPPSHCLVHLHKVSWGKLISVPFLRP-IQGIFVILC-PRPGKSVINFV
Sciurus_carolin AQEAVATQLGLKEPPSHCLVHLHKVSWGKLISVPFWRP-IQGIFVTVC-PRPGKSVINFV
Sciurus_niger@P AQEAVATQLGLKEPPSHCLVHLHKVSWGKLISVPFWRP-IQGIFVTLC-PRPGKSVINFV
                ****.******************:

Ppox            --------NCIPQYTIGHWQK--------------------------LDSAMQFLTAQRL
Aplodontia_rufa --------NCIPQYTLGHWQKLGKLGKLGGGQRKSDL-KLHSFHPFLPESAMKFLSAQKL
Xerus_inauris@P --------NCIPQYTLGHWQKLGKLGKQLGELRKSDLQPQNSFHPFLLESAMKFLAAQRL
Marmota_monax@P LYPLSSSQNCIPQYTLGHWQK--------------------------LESAMKFLAAQRL
Spermophilus_da LYPLSSSQNCIPQYTLGHWQK--------------------------LESAMKFLAAQRL
Ictidomys_tride --------NCIPQYTLGHWQKLGKVGKQLGGLRK-DL-PQNSFYSFLLESAMKFLAAQRL
Urocitellus_par LYPLSSSQNCIPQYTLGHWQKLGKVGKQLGGLRK-DL-PQNSFYSFLLESAMKFLGAQRL
Sciurus_vulgari LYSLPSSQNCIPQYTLGHWQKLGKFGKQLGGLKKSDL-PQNSFYPFLPESAMKFLAAQKL
Sciurus_carolin LYPLPSSQNCIPQYTLGHWQKLGKFGKQLGGLKKSDL-PQNSFHPFLPESAMKFLAAQKL
Sciurus_niger@P LYPLPSSQNCIPQYTLGHWQKLGKFGKQLGGLKKSDL-PQNSFHPFLPESAMKFLAAQKL
                        *******:*****                           :***:** **:*

Ppox            PLTLAGASYEGVAVNDCIESGRQAAVAVLGTESNSZ
Aplodontia_rufa PLTLAGASYEGVAVNDCIESGRQAAVSVLGTESNS-
Xerus_inauris@P PLTLAGASYEGVAVNDCIESGRQAAISVLGPESNS-
Marmota_monax@P PLTLAGASYEGVAVNDCIESGRQAAISVLGPESNS-
Spermophilus_da PLTLAGASYEGVAVNDCIESGRQAAISVLGPESNS-
Ictidomys_tride PLTLAGASYEGVAVNDCIESGRQAAISVLGPESNS-
Urocitellus_par PLTLAGASYEGVAVNDCIESGRQAAISVLGPESNS-
Sciurus_vulgari PLTLAGASYEGVAVNDCIESGRQAAISVLGPESN--
Sciurus_carolin PLTLAGASYEGVAVNDCIESGRQAAISVLGPES---
Sciurus_niger@P PLTLAGASYEGVAVNDCIESGRQAAISVLGPESN--
                *************************::***.**
```

UROD encodes uroporphyrinogen decarboxylase. Diseases associated with *UROD* include porphyria cutanea tarda and familial porphyria cutanea tarda. Among its related pathways are biosynthesis of cofactors and metabolism. Gene ontology (GO) annotations related to this gene include uroporphyrinogen decarboxylase activity. This gene encodes an enzyme responsible for catalyzing the conversion of uroporphyrinogen to coproporphyrinogen through the removal of

four carboxymethyl side chains. Mutations and deficiency in this enzyme are known to cause familial porphyria cutanea tarda and hepatoerythropoetic porphyria.

Within a 426-amino acid *UROD* sequence for *S. niger*, we observed nine point-differences among sciurids, including one contiguous tract of six where the *S. niger* sequence differed from those of *S. vulgaris* and *S. carolinensis*. *S. carolinensis* had a deletion of 38 amino acids relative to the sequences of *S. vulgaris* and *S. niger*. Comparing among all species, we observed insertions of 15 and 15 amino acids in *A. rufa* and 50 in *S. dauricus* and *U. parryii*.

```
Urod              MEANGFGLQNFPELKNDTFLRAAWGEETDYTPVWCMRQAGRYL------P----------
Aplodontia_rufa   --------QTFPELKNDTFLRAAWGEETDYTPVWCMRQAGRYLPGQGQGPEVINCVC---
Xerus_inauris@U   --------QSFPELKNDTFLRAAWGQETDYTPVWCMRQAGRYLPGQGQGP----------
Marmota_monax@U   --------QNFPELKNDTFLRAAWGEETDYTPVWCMRQAGRYL------P----------
Spermophilus_da   --------QNFPELKNDTFLRAAWGEETDYTPVWCMRQAGRYL------P----------
Ictidomys_tride   --------QNFPELKNDTFLRAAWGEETDYTPVWCMRQAGRYL------P----------
Urocitellus_par   --------QNFPELKNDTFLRAAWGEETDYTPVWCMRQAGRYL------P----------
Sciurus_vulgari   --------QSFPELKNDTFLRAAWGEETDYTPVWCMRQAGRYLPGEGQGPEFITLGSGGW
Sciurus_carolin   --------QSFPELKNDTFLRAAWGEETDYTPVWCMRQAGRYLPGQGQGPEFITLGSGGW
Sciurus_niger@U   --------QSFPELKNDTFLRAAWGEETDYTPVWCMRQAGRYLPGQGQGPEFITLGSGGW
                          *.***************:*****************      *

Urod              ----------------EFRETRAAQDFFSTCRSPEACCELT-------------------
Aplodontia_rufa   -MSRASRLRSVFLLFAEFRKTRATQDFFSTCRSPEACCELT-------------------
Xerus_inauris@U   ----------------EFRETRAAQDFFSTCRSPEACCELTLQVRGPEREVFMPSAYRLP
Marmota_monax@U   ----------------EFRETRATQDFFSTCRSPEACCELTLQVRGPQREVFMPTACHLA
Spermophilus_da   ----------------EFRETRATQDFFSTCRSPEACCELTLQVRSPQREVFMPSACHLA
Ictidomys_tride   ----------------EFRETRATQDFFSTCRSPEACCELTLQVRGPQREVFMPSACHLA
Urocitellus_par   ----------------EFRETRATQDFFSTCRSPEACCELTLQVRGPQREVFMPSACHLA
Sciurus_vulgari   GGALGARLRSAFLLSAEFRETRATQDFFSTCRSPEACCELTLQVRGPQREVFMSSACHLA
Sciurus_carolin   GGALGARLRSAFLLSAEFRETRATQDFFSTCRSPEACCELT-------------------
Sciurus_niger@U   GGALGARLRSAFLLSAEFRETRATQDFFSTCRSPEACCELT-------------------
                                  ***:***:*****************

Urod              -----LQPLRRFPLDAAIIFSDILVVPQALGMEVTMVPGKGPSFPEPLREERDLERLRDP
Aplodontia_rufa   -----LQPLRRFPLDAAIIFSDILVVPQALGLEVTMEPGKGPSFPEPLREERDLERLRDP
Xerus_inauris@U   TCFHSLQPLRRFPLDAAIIFSDILVVPQALGMEVIMVPGKGPSFPEPLREERDLERLRDP
Marmota_monax@U   TRFHSLQPLRRFPLDAAIIFSDILVVPQALGMEVTMVPGKGPSFPEPLREERDLERLRDP
Spermophilus_da   TRFHSLQPLRRFPLDAAIIFSDILVVPQALGMEVTMVPGKGPSFPEPLREERDLERLRDP
Ictidomys_tride   TRFHSLQPLRRFPLDAAIIFSDILVVPQALGMEVTMVPGKGPSFPEPLREERDLERLRDP
Urocitellus_par   TRFHSLQPLRRFPLDAAIIFSDILVVPQALGMEVTMVPGKGPSFPEPLREERDLERLRDP
Sciurus_vulgari   TCFHSLQPLRRFPLDAAIIFSDILVVPQALGMEVTMVPGKGPSFSEPLREEQDLERLRDP
Sciurus_carolin   -----LQPLRRFPLDAAIIFSDILVVPQALGMEVTMVPGKGPSFSEPLREERDLERLRDP
Sciurus_niger@U   -----LQPLRRFPLDAAIIFSDILVVPQALGMEVTMVPGKGPSFSEPLREERDLERLRDP
                       **************************:** * *******.******:********

Urod              AAAASELGYVFQAITLTRQRLAGRVPLIGFAGAP--------------------------
Aplodontia_rufa   AAVASELNYVFQAITLTRQQLAGRVPLIGFAGAPVIWEEQGLGHEITLADLVLTREGGVS
Xerus_inauris@U   SVVASELGYVFQAITLTRQQLAGRVPLIGFAGAP--------------------------
Marmota_monax@U   EVVTSELGYVFQAITLTRQQLAGRVPLIGFAGAP--------------------------
Spermophilus_da   EVVTSELGYVFQAITLTRQQLAGRVPLIGFAGAPVIWEKQGIGALWHI--WSRGRKCQCG
Ictidomys_tride   EVVTSELGYVFQAITLTRQQLAGRVPLIGFAGAPVIWEKQGTGALWHI--WCRGRKCECG
Urocitellus_par   EVVTSELGYVFQAITLTRQQLAGRVPLIGFAGAPVTWEKQGIGALWHI--WCRGRKCQCG
Sciurus_vulgari   AVVASELGYVFQAITLTRQQLAGRVPLIGFAGAPVIWDKQGTGTDHAG--ISSIDKGSVN
Sciurus_carolin   AVVASELGYVFQAITLTRQQLAGRVPLIGFAGAP--------------------------
Sciurus_niger@U   AVVASELGYVFQAITLTRQQLAGRVPLIGFAGAPVTWDKQGTGADHAG--VSSIDKGSVS
                   ..:***.***********:**************
```

```
Urod            ------------WTLMTYMVEGGSSSTMAQAKRWLYQRPQASHKLLGILTDVLVPYLIGQ
Aplodontia_rufa LTSALHLYILL-WTLMTYMVEGGSSSTMAQSKRWLYQRPQASHKLLGILTDALVPYLVGQ
Xerus_inauris@U ------------WTLMTYMVEGGGSSTMAQSKRWLYQRPQASHQLLRILTDALVPYLVGQ
Marmota_monax@U ------------WTLMTYMVEGGSSSTMAQSKRWLYQRPQASHQLLRILTDALVPYLVGQ
Spermophilus_da FCTVTSL-ILLQWTLMTYMVEGGSSSTMAQSKRWLYQRPQASHQLLRILTDALVPYLVGQ
Ictidomys_tride FCTVTSL-ILLQWTLMTYMVEGGSSSTMAQSKRWLYQRPQASHQLLRILTDALVPYLVGQ
Urocitellus_par FCTVTSL-ILLQWTLMTYMVEGGSSSTMAQSKRWLYQRPQASHQLLRILTDALVPYLVGQ
Sciurus_vulgari VASALRLCILLQWTLMTYMVEGGSSSTMAQSKRWLYQRPQASHQLLRILTDVLVPYLI--
Sciurus_carolin ------------WTLMTYMVEGGSSSTMAQSKRWLYQRPQASHQLLRILTDVLVPYLI--
Sciurus_niger@U VASALHLCILLQWTLMTYMVEGGSSSTMAQSKRWLYQRPQASHQLLRILTDALVPYLVGQ
                            ***********.******:************:** ****.*****:

Urod            VAAGA-QALQLFESHAGHLGTELFSKFALPYIRDVAKRVKAGLQKAGLAPVPM-------
Aplodontia_rufa VPRQVPRHCSSFESHAGHLGTELFSKFALPYIRDVAKRVKAGLQKAGLAPVPM-------
Xerus_inauris@U VAAGA-QALQLFESHAGHLGPQLFNKFSLPYIRDVAKRVKARLQEAGLAPVPM-------
Marmota_monax@U VAAGA-QALQLFESHAGHLGPQLFNKFALPYIRDVAKRVKARLQEAGLAPVPM-------
Spermophilus_da VAAGA-QALQLFESHAGHLGPQLFNKFALPYIRDVAKRVKARLQEAGLAPVPMVRIGIGW
Ictidomys_tride VAAGA-QALQLFESHAGHLGPQLFNKFALPYIRDVAKRVKARLQEAGLAPVPM-------
Urocitellus_par VAAGA-QALQLFESHAGHLGPQLFNKFALPYIRDVAKRVKARLQEAGLAPVPMVRIGIGW
Sciurus_vulgari FFTTL-LALQLFESHAGHLGPQLFSKFALPYIRDVAKRVKAKLQEAGLAPVPM-------
Sciurus_carolin FFTTL-LALQLFESHAGHLGPQLFSKFALPYIRDVAKRVKAKLQEAGLAPVPM-------
Sciurus_niger@U VAAGA-QALQLFESHAGHLGPQLFSKFALPYIRDVAKRVKAKLQEAGLAPVPM-------
                .        . *********.:**.**:************* **:********

Urod            --------------------------------------------IIFAKDGHFALEELAQ
Aplodontia_rufa --------------------------------------------IIFAKDGHFALEELAQ
Xerus_inauris@U --------------------------------------------IIFAKDGHFALEELAQ
Marmota_monax@U --------------------------------------------IIFAKDGHFALEELAQ
Spermophilus_da WVCRVFRVSLAWTEVTTGGQQKCIQERLVGTARPYVSQIRFFLQIIFAKDGHFALEELAQ
Ictidomys_tride --------------------------------------------IIFAKDGHFALEELAQ
Urocitellus_par WVCRVFRVSHAWTEVTTGGQQKCIQERLVGTARPYVSQILFFLQIIFAKDGHFALEELAQ
Sciurus_vulgari --------------------------------------------IIFAKDGHFALEELAQ
Sciurus_carolin --------------------------------------------IIFAKDGHFALEELAQ
Sciurus_niger@U --------------------------------------------IIFAKDGHFALEELAQ
                                                            ****************

Urod            AGYEVVGLDWTVAPKKARERVGKAVTLQGNLDPCALYASEEEIGRLVQQMLDDFGPQRYI
Aplodontia_rufa AGYEVVGLDWTVAPKKARERVGKMVTLQGNLDPCALYASEEEIGRLVKQMLDDFGPQHYI
Xerus_inauris@U AGYEVVGLDWTMAPKKARERVGKTVTLQGNLDPCALYASEEEIGRLVQQMLDDFGPQRYI
Marmota_monax@U AGYEVVGLDWTVAPKKARERVGKSVTLQGNLDPCALYASEEEIGRLVQQMLDDFGPKRYI
Spermophilus_da AGYEVVGLDWTVAPKKARERVGKSVTLQGNLDPCALYASEEEIGRLVQQMLDDFGPKRYI
Ictidomys_tride AGYEVVGLDWTVAPKKARERVGKSVTLQGNLDPCALYASEEEIGRLVQQMLDDFGPKRYI
Urocitellus_par AGYEVVGLDWTVAPKKARERVGKSVTLQGNLDPCALYASEEEIGQLVQQMLDDFGPKRYI
Sciurus_vulgari AGYEVVGLDWTVAPKKARERVGKTVTLQGNLDPCALYASEEEIGQMVQKMLDDFGPQRYI
Sciurus_carolin AGYEVVGLDWTVAPKKARERVGKTVTLQGNLDPCALYASEEEIGQMVQKMLDDFGPQRYI
Sciurus_niger@U AGYEVVGLDWTVAPKKARERVGKTVTLQGNLDPCALYASEEEIGQMVQKMLDDFGPQRYI
                ***********:*********** ********************::*::*******::**

Urod            ANLGHGLYPDMDPERVGAFVDAVHKHSRLLRQNZ
Aplodontia_rufa ANLGHGLYPDMDPEHVGAFVDAVHRHSRLLRQN-
Xerus_inauris@U ANLGHGLYPDMDPEHVGAFVDAVHRHSRLLRQN-
Marmota_monax@U ANLGHGLYPDMDPEHVGAFVDAVHRHSRLLRQN-
Spermophilus_da ANLGHGLYPDMDPEHVGAFVDAVHRHSRLLRQN-
Ictidomys_tride ANLGHGLYPDMDPEHVGAFVDAVHRHSRLLRQN-
Urocitellus_par ANLGHGLYPDMDPEHVGAFVDAVHRHSRLLRQN-
```

```
Sciurus_vulgari ANLGHGLYPDMDPEHVGAFVDAVHRHSRLLRQN-
Sciurus_carolin ANLGHGLYPDMDPEHVGAFVDAVHRHSRLLRQN-
Sciurus_niger@U ANLGHGLYPDMDPEHVGAFVDAVHRHSRLLRQN-
                **************:*********:********
```

UROS encodes uroporphyrinogen III synthase. Diseases associated with *UROS* include porphyria and congenital erythropoietic and cutaneous porphyria, or Gunther's disease. Among its related pathways are porphyrin metabolism and biosynthesis of cofactors. Gene ontology (GO) annotations related to this gene include cofactor binding and uroporphyrinogen-III synthase activity. The protein encoded by this gene catalyzes the fourth step of porphyrin biosynthesis in the heme biosynthetic pathway. The human UROS protein has 199 amino acids before any squirrel one. *M. monax*, *S. dauricus*, *I. tridecemlineatus*, and *U. parryii* had an 11-amino acid sequence not similar to the human sequence. From amino acid 419, the sequences of all species were similar. The *S. niger* amino acid sequence differed from those of *S. vulgaris* and *S. carolinensis* at amino acid 229, and the *S. carolinensis* sequence from those of *S. vulgaris* and *S. niger* at amino acid 256.

```
Uros            MKVLLLKDAKEDDSGLDPYIQELRLCGLEATLIPVLSFEFMSLPSLSEKLSHPEGFGGLI
Aplodontia_rufa ------------------------------------------------------------
Xerus_inauris@U ------------------------------------------------------------
Marmota_monax@U ------------------------------------------------------------
Spermophilus_da ------------------------------------------------------------
Ictidomys_tride ------------------------------------------------------------
Urocitellus_par ------------------------------------------------------------
Sciurus_vulgari ------------------------------------------------------------
Sciurus_carolin ------------------------------------------------------------
Sciurus_niger@U ------------------------------------------------------------


Uros            FTSPRAVEAVKLCLEKDNKTEAWEKSLKDRWNAKSVYVVGSATASLVNKIGLDAEGAGSG
Aplodontia_rufa ------------------------------------------------------------
Xerus_inauris@U ------------------------------------------------------------
Marmota_monax@U ------------------------------------------------------------
Spermophilus_da ------------------------------------------------------------
Ictidomys_tride ------------------------------------------------------------
Urocitellus_par ------------------------------------------------------------
Sciurus_vulgari ------------------------------------------------------------
Sciurus_carolin ------------------------------------------------------------
Sciurus_niger@U ------------------------------------------------------------


Uros            NAEKLAEYICSKPSSELPLLFPCGTIKGDTLPKMLKDKGIPMESMHVYQTVPHPGIQGSL
Aplodontia_rufa ------------------------------------------------------------
Xerus_inauris@U ------------------------------------------------------------
Marmota_monax@U ------------------------------------------------------------
Spermophilus_da ------------------------------------------------------------
Ictidomys_tride ------------------------------------------------------------
Urocitellus_par ------------------------------------------------------------
Sciurus_vulgari ------------------------------------------------------------
Sciurus_carolin ------------------------------------------------------------
Sciurus_niger@U ------------------------------------------------------------
```

```
Uros            KSYYEDQGIPASITFFSPSGLKYSLEYIQALSGSSFDQIKFIAIGPSTTRAMAAKGLPVS
Aplodontia_rufa ---------------------------------------QFAAIGPSTARALAAKGLPVS
Xerus_inauris@U ---------------------------------SSLPSLQFAAIGPTTARALTSKGLPVS
Marmota_monax@U -------------------GVMYRKALAEA---LSLPSLQFAAIGPTTAHALTAKGLPVS
Spermophilus_da -------------------GVMYRKALAEA---LSLPSLQFAAIGPTTAHALTAKGLPVS
Ictidomys_tride -------------------GVMYQKALAEA---LSLPSLQFAAIGPTTAHALTAKGLPVS
Urocitellus_par -------------------GVTYRKALAEA---LSLPSLQFAAIGPTTAHALTAKGLPVS
Sciurus_vulgari ---------------------------------SSLPSSQFAAIGPTTARALAAQGLPVS
Sciurus_carolin ----------------------------------------FAAIGPTTARALAAQGLPVS
Sciurus_niger@U ----------------------------------SLSSSQFAAIGPTTARTLAAQGLPVS
                                                        * ****:*:::::::*****

Uros            CTAESPTPQALAAGIRNVLKPNHCCZ
Aplodontia_rufa CTAESPTPQALAASIRKALQPHSCC-
Xerus_inauris@U CTAESPTPQALAAGIRKALQPQGCC-
Marmota_monax@U CTAESPTPQALAAGIRKALQPQGCC-
Spermophilus_da CTAESPTPQALAAGIRKALQPQGCC-
Ictidomys_tride CTAESPTPQALAAGIRKALQPQGCC-
Urocitellus_par CTAESPTPQALAAGIRKALQPQGCC-
Sciurus_vulgari CTAESPTPQALAAGIRKALQPQ----
Sciurus_carolin CTAESPTPQALAAGIWKALQPQGSC-
Sciurus_niger@U CTAESPTPQALAAGIRKALQPQGSC-
                ************.* :.*:*:
```

Literature Cited

O'Malley R, Rao G, Stein P, Bandmann O. 2018. Porphyria: often discussed but too often missed. Pract Neurol. 18: 352-358.

Phillips JD. 2019. Heme biosynthesis and the porphyrias. Molec Genet Metab. 128: 164-177.

Rimington C. 1989. Haem biosynthesis and porphyrias: 50 years in retrospect. J Clin Chem Clin Biochem. 27: 473-486.
