# Supplementary material for: A draft genome assembly for the eastern fox squirrel, Sciurus niger
Source: G3 (Bethesda). 2021 Sep 22;11(12):jkab315. doi: 10.1093/g3journal/jkab315 (PMC8664420; doi:10.1093/g3journal/jkab315)
Supplement: jkab315_Supplementary_File_S3 [file jkab315_supplementary_file_s3.pdf]

Supplemental File S3 – Color vision-related genes

Because most squirrel species, including *S. niger*, are diurnal mammals with cone-dominated retinas, similar to primate retinas (Van Hooser and Nelson 2006), we examined variation of five color vision-related genes (El Moussawi *et al*. 2021) among our focal species.

ATF6 encodes activating transcription factor 6. Diseases associated with *ATF6* include achromatopsia 7 and achromatopsia. Among its related pathways are metabolism of proteins and the TNFR1 pathway. Gene ontology (GO) annotations related to this gene include DNA-binding transcription factor activity and protein heterodimerization activity. This gene encodes a transcription factor that activates target genes for the unfolded protein response (UPR) during endoplasmic reticulum (ER) stress. Although it is a transcription factor, this protein is unusual in that it is synthesized as a transmembrane protein that is embedded in the ER. It functions as an ER stress sensor/transducer, and following ER stress-induced proteolysis, it functions as a nuclear transcription factor via a cis-acting ER stress response element that is present in the promoters of genes encoding ER chaperones.

Compared with the sequences of congeners, the 536-amino acid sequence of ATF6 for *S. niger* shows 52 point-differences. Further, stretches of 35 and 36 amino acids of *S. vulgaris* ATF6 do not match those of *S. carolinensis* and *S. niger*, the first following the insertion of a leucine and the second after insertion of six amino acids. *A. rufa* and *S. vulgaris* have a deletion of 28 amino acids.

```
Atf6              LYGDSCNSPSSVEPLKEEKPVTGPGNKTEHGLTPKKKIQMSSKPSVQPKPLLLPAAPKTQ
Aplodontia_rufa   ----------------------------ENGLIPKKKIQMSSKPSIQPKPLLLPAAPKTQ
Xerus_inauris@A   ----------------------------ENGLTPKKKIQINSKPSIQPKPLLLPAAPKTQ
Marmota_monax@A   ----------------------------ENGLTPKKKIQMGSKPSVQPKPLLLPAAPKTQ
Spermophilus_da   ----------------------------ENGLTPKKKIQMGSKPSIQPKPLLLPAAPKTQ
Ictidomys_tride   ------------------------------------------------------------
Urocitellus_par   ----------------------------ENGLTPKKKIQMGSKPSIQPKPLLLPAAPKTQ
Sciurus_vulgari   ----------------------------ENGLTPKKKIQMSSKPSIQPKPLLLPAAPKTQ
Sciurus_carolin   ---------------------------SENGLTPKKKIQMSSKPSIQPKPLLLPAAPKTQ
Sciurus_niger@A   ----------------------------ENGLTPKKKIQMSSKPSIQPKPLLLPAAPKTQ


Atf6              TNASVPAKAIIIQTLPALMPLAKQQSIISIQPAPTKGQTVLLSQPTVVQLQSPAVLSSAQ
Aplodontia_rufa   TNSSVPAKTIIIQTLPTLMPLTKQQPIISIQPTPTKGQTVLLSQPTVVQLQAPGVLPSAQ
Xerus_inauris@A   TNSTVPAKTIIIQTLPTLMPLTKQQPIISIQPASSKGQTVLLSQPAVVQLQAPGVLPSAQ
Marmota_monax@A   TNSSVPAKTIIIQTLPTLMPLAKQQPIISIQPAPTKGQTVLLSQPTVVQLQAPGVLPAAQ
Spermophilus_da   TNSSVPAKTIIIQTLPTLMPLAKQQPIISIQPAPTKGQTVLLSQPTVVQLQTPGVLPSAQ
Ictidomys_tride   ------------------------------------------------------------
Urocitellus_par   TNSSVPAKTIIIQTLPTLMPLAKQQPIISIQPAPTKGQTVLLSQPTVVQLQAPGVLPSAQ
Sciurus_vulgari   TNSSVPAKTIIIQTLPALMPLAKQQSIISIQPAPTKGQTVLLSQPTVVQLQSPAVLSSAQ
Sciurus_carolin   TNSSVPAKTIIIQTLPTLMPLTKQQSIISIQPAPTKGQTVLLSQPTVVQLQAPGVLPSAQ
Sciurus_niger@A   TNSSVPAKTIIIQTLPTLMPLTKQQPIISIQPAPTKGQTLLLSQPTVVQLQAPGVLPSAQ


Atf6              PVLAVTGGAAQLPNHVVNVLPAPVVSSPVNGKLSVTKPVLQSATRSMGSDIAVLRRQQRM
Aplodontia_rufa   PVLAVPGGASQLPNHVVNVLPAPVVNSPVNGKLSVTKPVLQSPMKSVGSDIAVLRRQQRM
Xerus_inauris@A   PVLAVARGTTQLPNHVMNVVPAPVVNSPVNGKLSVTKPVLQSPMKSVGSDIAVLRRQQRM
Marmota_monax@A   PVLAVGGGATQLPNQVVNVVPAPVVNSPVNGKLSVTKPVLQSATRSMGSDIAVLRRQQRM
Spermophilus_da   PVLAVGGGATQLPNQVVNVVPAPVVNSPVNGKLSVTKTVLQSATRSMGSDIAVLRRQQRM
Ictidomys_tride   ------------------------------------------ASSSLGA-IAVLRRQQRM
```

```
Urocitellus_par PVLAVGGGATQLPNQVVNVVPAPVVNSPVNGKLSVTKPVLQSATRSMGSDIAVLRRQQRM
Sciurus_vulgari PVLAVTGGAAQLPNHVVNVLPAPVVSSPVNGKLSVTKPVLQSATRSMGSDIAVLRRQQRM
Sciurus_carolin LVLAVTGGATQLPNHVVNVVPAPVVNSSVNGKLSVTKPVLQSPMKSVGSDIAVLRRQQRM
Sciurus_niger@A PVLAVTGGATQLPNHVVNVVPAPVVNSSVNGKLSVTKPVLQSPMKSVGSDIAVLRRQQRM
                                                          .  *:*: **********

Atf6            IKNRESACQSRKKKKEYMLGLEARLKAALSENEQLKKENGSLKRQLDEVVSE--------
Aplodontia_rufa IKNRESACQSRKKKKEYMLGLEARLKAALSENEQLKKENGSLKRQLDEVVSE--------
Xerus_inauris@A IKNRESACQSRKKKKEYMLGLEARLKAALSENEQLKKENGSLKRQLDEVVSEVRLDCPWE
Marmota_monax@A IKNRESACQSRKKKKEYMLGLEARLKAALSENEQLKKENGSLKRQLDEVVSEVRLDCPWE
Spermophilus_da IKNRESACQSRKKKKEYMLGLEARLKAALSENEQLKKENGSLKRQLDEVVSEVRLDCPWE
Ictidomys_tride IKNRESACQSRKKKKEYMLGLEARLKAALSENEQLKKENGSLKRQLDEVVSEVRLDCPWE
Urocitellus_par IKNRESACQSRKKKKEYMLGLEARLKAALSENEQLKKENGSLKRQLDEVVSEVRLDCPWE
Sciurus_vulgari IKNRESACQSRKKKKEYMLGLEARLKAALSENEQLKKENGSLKRQLDEVVSE--------
Sciurus_carolin IKNRESACQSRKKKKEYMLGLEARLKAALSENEQLKKENGSLKRQLDEVVSEVRLDCPWE
Sciurus_niger@A IKNRESACQSRKKKKEYMLGLEARLKAALSENEQLKKENGSLKRQLDEVVSEVRLDCPWE
                ****************************************************

Atf6            --------------------NQRLKVPSPKRRAVCVMIVLAFIMLNYGPMSMLEQESRRV
Aplodontia_rufa --------------------NQRLKVPSGKRRAVCVMIVLAFIMLNYGPMSMLEQESRRV
Xerus_inauris@A MGWNIASVGPPVSFAFLSAPNSELKLGSGNRKVVCVMIVLAFIMLNFGPMSMLEQDSRRM
Marmota_monax@A MGCNTGSTRHPVSFTSPSAPNSELKLGSGNRKVVCIMVFLLFIAFNFGPMSMLEQDSRRM
Spermophilus_da MGCNTGSTQHPVSFASPSAPNSELKLGSGNRKVVCIMVFLLFIAFNFGPMSMLEQDSRRM
Ictidomys_tride MGCNTGSTKHPVSFASPSAPNSELKLGSGNRKAVCVMIVLAFIALNFGPMSMLEQDSRRM
Urocitellus_par MGCNTGSTRHPVSFASPSAPNSELKLGSGNRKVVCIMVFLLFIAFNFGPMSMLEQDSRRM
Sciurus_vulgari --------------------NQRLKVGSGNRRAVCIMIVLAFIMLNYGPMSMLEQESRRV
Sciurus_carolin MVGIQAVWVDSDSFAS-SEPNSELKLGSGNRKAVCIMIVLAFIMLNYGPMSMLEQDSRRM
Sciurus_niger@A MVGIQAVWVDSDSFAS-SEPNSELKLGSGNRRAVCIMIVLAFIMLNYGPMSMLEQDSRRM
                                    *..**: * :*:.**:*:.* ** :*:********:***:

Atf6            KPSVSPANQRRHLLEFSAKEVKDTSDGDNQKDSYSYD-HSVSNDKAL------MVLSEEP
Aplodontia_rufa KPSVSPANQRRHLLEFSAKEVKDTSDGDNQKDSYSYD-SSVSNDKAL------MVLSEEP
Xerus_inauris@A NPSMSPANQRRHLLEFSAKEVQDTSDGIIQKNSYRLD-HSVSNDKAL------MVLSEEP
Marmota_monax@A NPNVSPANQRRHLLEFSAKEVQDTSDGIIQKNSYRLD-HSVSNDKAL------MVLSEEP
Spermophilus_da NPNVSPANQRRHLLEFSAKEVQDTSDGIIQKNSYRLD-HSVSNDKAL------MVLSEEP
Ictidomys_tride NPNVSPANQRRHLLEFSAKEVRDTSDGIIQKNSYRLD-HSVSNDKAL------MVLSEEP
Urocitellus_par NPNVSPANQRRHLLEFSTKEVQDTSDGIIQKNSYRLD-HSVSNDKAL------MVLSEEP
Sciurus_vulgari KPSMSPANQRRHLLEFSAKEVQDTSDGDNQKDSYSYDLPSFRNLTAFPGGAKELLLSDLD
Sciurus_carolin NPSMSPANQRRHLLEFSAKEVQDTSDGIIQKNSYRLD-HSVSNDKAL------MVLSEEP
Sciurus_niger@A NPNMSPANQRRHLLEFSAKEVQDTSDGIIQKNSYRLD-HSVSNDKAL------MVLSEEP
                :*.:*************:***:*****  **:**  *  *. * .*:      ::**:

Atf6            LLYMPPPPCQPLINTTESLRLNHELRGWVHRHEVERTKSRRMTNSQQKARILQGALEQGS
Aplodontia_rufa LLYMPPPPCQPLINTTESLRLNHELRGWVHRHEVERTKSRRMTNSQQKARILQGALEQGS
Xerus_inauris@A LLYMPPPPCQPLINTTESLRLNHELRGWVHRHEVERTKSRRMTNSQQKARILQGALEQGS
Marmota_monax@A LLYMPPPPCQPLINTTESLRLNHELRGWVHRHEVERTKSRRMTNNQQKTRVLQVCFYLGS
Spermophilus_da LLYMPPPPCQPLINTTESLRLNHELRGWVHRHEVERTKSRRMTNSQQKARILQGALEQGR
Ictidomys_tride LLYMPPPPCQPLINTTESLRLNHELRGWVHRHEVERTKSRRMTNSQQKARILQGALEQGS
Urocitellus_par LLYMPPPPCQPLINTTESLRLNHELRGWVHRHEVERTKSRRMTNSQQKARILQGALEQGR
Sciurus_vulgari QLFL-SSDCRHF-NRTESLRLADELSGWVQRHERGRRKVPQRTQ-ERKSQLWK------K
Sciurus_carolin LLYMPPPPCQPLINTTESLRLNHELRGWVHRHEVERTKSRRMTNSQQKARILQGALEQGS
Sciurus_niger@A LLYMPPPPCQPLINTTESLRLNHELRGWVHRHEVERTKSRRMTNSQQKARILQGALEQGS
                 *:: .. *: : * ****** .** ***:***  * *  : *: ::*::: :

Atf6            NSQLMAVQYTETTSISRNSGSELQVYYASPGSYQGFFDAIRRRGDTFYVVSFRRDHLLLP
Aplodontia_rufa NSQLMAVQYTETTSFSGNSGNELQVYYASPRSYQDFFEAIRRRGDTFYVVSFRRDHLLLP
```

```
Xerus_inauris@A NSQLMAVQYTETTSFSRNSGNELQVYYASPRSYQDFFEAIRRRGDTFYVVSFRRDHLLLP
Marmota_monax@A NSQLMAVQYTETTSISRNSGSELQVYYASPGSYQDFFEAIRRRGDTFYVVSFRRDHLLLP
Spermophilus_da IRHFLLVNTLPFSYFSRNSGNELQVYYASPRSYQDFFEAIRRRGDTFYVVSFRRDHLLLP
Ictidomys_tride NSQLMAVQYTETTSISRNSGSELQVYYASPGSYQGFFDAIRRRGDTFYVVSFRRDHLLLP
Urocitellus_par IRHFLLVNTLPFSYFSRNSGNELQVYYASPRSYQDFFEAIRRRGDTFYVVSFRRDHLLLP
Sciurus_vulgari SPPVKAVPTQPPTSISRNSGSELQVYYASPRSYQGFFDAIRRRGDTFYVVSFRRDHLLLP
Sciurus_carolin NSQLMAVQYTETTYFSRNSGNELQVYYASPRSYQDFFEAIHRRGDTFYVVSFRRDHLLLP
Sciurus_niger@A NSQLMAVQYTETTSISRNSGSELQVYYASPGSYQGFFDAIRRRGDTFYVVSFRRDHLLLP
                   .  *     : :* ***.********* ***.**:**:*******************

Atf6            ATTHNKTTRPKMSIVLPAININDNVINGQDYEVMMQIDCQVMDTRILHIKSSSVPPYLRD
Aplodontia_rufa ATTHNKTTRPKMSIVLPAINFTENVINGQDYEVMMQIDCQVMDTRILHIKSSSVPPYLRD
Xerus_inauris@A ATTHNKTTRPKMSIVLPAINITENVINGQDYEVMMQIDCQVMDTRILHIKSSSVPPYLRD
Marmota_monax@A ATTHNKTTRPKMSIVLPAINITENVINGQDYEVMMQIDCQVMDTRILHIKSSSVPPYLRD
Spermophilus_da ATTHNKTTRPKMSIVLPAINITENVINGQDYEVMMQIDCQVMDTRILHIKSSSVPPYLRD
Ictidomys_tride ATTHNKTTRPKMSIVLPAININENVINGQDYEVMMQIDCQVMDTRILHIKSSSVPPYLRD
Urocitellus_par ATTHNKTTRPKMSIVLPAINITENVINGQDYEVMMQIDCQVMDTRILHIKSSSVPPYLRD
Sciurus_vulgari AISHNKTSRPKMSLVMPAMAPNENVINGQDYEVMMQIDCQVMDTRILHIKSSSVPPYLRD
Sciurus_carolin ATTHNKTTRPKMSIVLPAININENVINGQDYEVMMQIDCQVMDTRILHIKSSSVPPYLRD
Sciurus_niger@A ATTHNKTTRPKMSIVLPAINITENVINGQDYEVMMQIDCQVMDTRILHIKSSSVPPYLRD
                * :****:*****:*:**:  .:*************************************

Atf6            HQRNQTSTFFGSPPTTTETTHVVSTIPESLQZ
Aplodontia_rufa QQRNQTNTFFGSPPAATKTTHVVSTIPESLQ-
Xerus_inauris@A QQRNQTSTFFGSPPAATKATHVVSTIPESLQ-
Marmota_monax@A QQRNQTNTYFGSPPAATKATHVVSTIPESLQ-
Spermophilus_da QQRNQTNTYFGSPPAATKATHVVSTIPESLQ-
Ictidomys_tride QQRNQTNTYFGSPPAATKATHVVSTIPESLQ-
Urocitellus_par QQRNQTNTYFGSPPAATKATHVVSTIPESLQ-
Sciurus_vulgari QQRNQTNTFFGSPPAATKATHVVSTIPESLQ-
Sciurus_carolin QQRNQTNTFFGSPPAATKATHVVSTIPESLQ-
Sciurus_niger@A QQRNQTNTFFGSPPAATKATHVVSTIPESLQ-
                :*****.*:*****::*::************
```

CNGA3 encodes cyclic nucleotide gated channel subunit alpha 3, a member of the cation channel protein family which is required for normal vision and olfactory signal transduction. Mutations in this gene are associated with achromatopsia (rod monochromacy) and color blindness. Two alternatively spliced transcripts encoding different isoforms have been described. Gene ontology (GO) annotations related to this gene include protein C-terminus binding and voltage-gated potassium channel activity.

The 630-amino acid sequences of *S. niger* exhibits eight point-differences from other members of genus *Sciurus*. The three tree squirrels show an insertion of 9-10 amino acids relative to all other species at positions 134-143 or 144. *A. rufa* has a two-amino acid insertion at positions 170-171, after which is a sequence of 35 amino acids that differs from those of the other species. There is a deletion of three amino acids at positions 213-215 in *M. monax* and *S. dauricus*. Starting at position 218, there is a stretch of 445 amino acids showing just three point-differences among all species studied.

```
Cnga3           MAKVNTQCSQPSPTQLSIKNADRDLDHVENGLGRSHSPCEETSSTLQQGIAMETRGLAGS
Aplodontia_rufa ------------------------------------------------------------
Xerus_inauris@C ------------------------------------------------------------
Marmota_monax@C ------------------------------------------------------------
Spermophilus_da ------------------------------------------------------------
```

```
Ictidomys_tride ------------------------------------------------------------
Urocitellus_par ------------------------------------------------------------
Sciurus_vulgari ------------------------------------------ASLLLQDLRRPGRGLCPS
Sciurus_carolin ------------------------------------------ASLLLQGLQRPGRGLCPS
Sciurus_niger@C -----------------------------------------------QGLRRPGRGLCPS


Cnga3           ARSVVTSQG---PARVSRLIISIRAWASRHLHDEDQTPDSFLDRFHGSELKEVSTRESNA
Aplodontia_rufa ------------PSRLSRLLISLRRWAASHSHREDQRPDSFLDRFRGAELKEVASQESNA
Xerus_inauris@C ------------PSRLARLIISLRTWAARHTHCEDQRPDSLLDRFRGAELREVSSQESNA
Marmota_monax@C ----LPSDG---PSRLARLIISLRTWAAGHSRCEDQRSDSLLDRFRGAELKEVSSQESNA
Spermophilus_da --------G---PSRFARLIISLRTWTTRHSCCEDQRSDSLLDRFHGAELKEVSSQESNA
Ictidomys_tride ------SDG---PSRLARLIISLRTWTARRSRCEDQRSDSPPDRFRGAELKEVSSQESNA
Urocitellus_par ------SDG---PSRLARLIISLRMWTARRSRREDQRSDSLLDRFRGAELKEVSSQESNA
Sciurus_vulgari S-----SDGCLSPSRLSRLIISLRTWATRHSRRKDPRPDSLLDPFRGAELKEVSSQESSA
Sciurus_carolin S-----SDGSLSPSRLSRLIISLRTWAARHSRRKDPRPDSLLDHFRGAELKEVSSQESST
Sciurus_niger@C S-----SDGCLSPSRLSRLIISLRTWAARHSRRKDPRPDSLLDRFRGAELKEVSSQESRD
                            *:*.:**:**:* *:: :   :*  .**  * *:*:**:**:::**

Cnga3           QPNPGEQKPPDGGE---------GRKEEPIVVDPSSNIYYRWLTAIALP--VFYNWCLLV
Aplodontia_rufa QLNTGSQEPPDRGE---------GRKEEPIVVDPSSNIYYRWLTAIALPCLLMYIWSMVI
Xerus_inauris@C LSHMGSQEPSDRGK----------REKEPLAVDPSSNIYYRWLTAIALP--VFYNWCLLV
Marmota_monax@C QSQAGSQEPPDRGE---------GRKEEPIVVDPSSNIYYRWLTAIALP--VFYNWCLLV
Spermophilus_da QSHAGSQEPPDRGE---------GRKEEPIVVDPSSNIYYRWLTAIALP--VFYNWVMLI
Ictidomys_tride QSHAGSQEPPDRGE---------GRKEEPIVVDPSSNIYYRWLTAIALP--SVYNWCLLV
Urocitellus_par WSHAGSQEPPDRGE---------GRKEEPIVVDPSSNIYYRWLTAIALP--VFYNWCLLV
Sciurus_vulgari QSHVGSQEPPDRGRRGKRPPTPAGRKEEPIVVDPSSNLYYRWLTAIAMP--VFYNWCLLV
Sciurus_carolin QSHVGSQEPPDRGRRGKRPPAPAGRKEEPIVVDPSSNLYYRWLTAIAMP--VFYNWCLLV
Sciurus_niger@C QSHVGSQEPPDRGRRGKRPPAPAGRKEEPIVVDPSSNLYYRWLTAIAMP--VFYNWCLLV
                  : *.*:*.* *.          *::**:.******:*********:*   .* * :::

Cnga3           CRACFDELQSEHLTLWLVLDYSADVLYVLDMLVRARTGFLEQGLMVRDTKRLWKHYTKTL
Aplodontia_rufa ARASFD-LESNDLEYWLIFNFSSDAGYLPGMFVRARTGFLEQGLMVRDTKRLWKHYTKTL
Xerus_inauris@C CRACFDELQSEHLMLWLVLDYSSDVIYGLDMLVRARTGFLEQGLMVRDTKRLWKHYTKTL
Marmota_monax@C CRACFDELQSEHLTIWLIFDFFSDVGCLPGMFV---TGFLEQGLMVRDTKRLWKHYTKTL
Spermophilus_da ARARFD-LQSNDLECWLIFDFFSDVGCLPGMFV---TGFLEQGLMVRDTKRLWKHYTKTL
Ictidomys_tride CRACFDELQSEHLTLWLVLDYSSDVLYVLDMLVRARTGFLEQGLMVRDTKRLWKHYTKTL
Urocitellus_par CRACFDELQSEHLTVFLVCLYELQHLYKQSLLPRTRTGFLEQGLMVRDTKRLWKHYTKTL
Sciurus_vulgari CRACFDELQSEHLTLWLVLDYSADVLYVLDMLVRARSGFLEQGLMVRDTKRLWKHYTKTL
Sciurus_carolin CRACFDELQSEHLTLWLVLDYSADVLYVLDMLVRARTGFLEQGLMVRDTKRLWKHYTKTL
Sciurus_niger@C CRACFDELQSEHLTLWLVLDYSADVLYVLDMLVRARTGFLEQGLMVRDTKRLWKHYTKTL
                .** ** *:*:.*  :*:  :  :     .::    :***********************

Cnga3           HFKLDILSLIPTDLAYLKLGVNYPELRFNRLLKFSRLFEFFDRTETRTNYPNVFRIGNLV
Aplodontia_rufa HFKLDILSLIPTDLAYLKLGVNYPELRFNRLLKFSRLFEFFDRTETRTNYPNVFRIGNLV
Xerus_inauris@C HFKLDILSLIPTDLAYLKLGVNYPELRFNRLLKFSRLFEFFDRTETRTNYPNVFRIGNLV
Marmota_monax@C HFKLDILSLIPTDLAYLKLGVNYPELRFNRLLKFSRLFEFFDRTETRTNYPNVFRIGNLV
Spermophilus_da HFKLDILSLIPTDLAYLKLGVNYPELRFNRLLKFSRLFEFFDRTETRTNYPNVFRIGNLV
Ictidomys_tride HFKLDILSLIPTDLAYLKLGVNYPELRFNRLLKFSRLFEFFDRTETRTNYPNVFRIGNLV
Urocitellus_par HFKLDILSLIPTDLAYLKLGVNYPELRFNRLLKFSRLFEFFDRTETRTNYPNVFRIGNLV
Sciurus_vulgari HFKLDILSLIPTDLAYLKLGVNYPELRFNRLLKFSRLFEFFDRTETRTNYPNVFRIGNLV
Sciurus_carolin HFKLDILSLIPTDLAYLKLGVNYPELRFNRLLKFSRLFEFFDRTETRTNYPNVFRIGNLV
Sciurus_niger@C HFKLDILSLIPTDLAYLKLGVNYPELRFNRLLKFSRLFEFFDRTETRTNYPNVFRIGNLV
                ************************************************************

Cnga3           LYTLIIIHWNACIYFAISKFIGFGTDSWVYPNTSKPEYARLSRKYIYSLYWSTLTLTTIG
```

```
Aplodontia_rufa LYTLIIIHWNACIYFAISKFIGFGTDSWVYPNTSKPEYARLSRKYIYSLYWSTLTLTTIG
Xerus_inauris@C LYTLIIIHWNACIYFAISKFIGFGTDSWVYPNTSKPEYARLSRKYIYSLYWSTLTLTTIG
Marmota_monax@C LYTLIIIHWNACIYFAISKFIGFGTDSWVYPNTSKPEYARLSRKYIYSLYWSTLTLTTIG
Spermophilus_da LYTLIIIHWNACIYFAISKFIGFGTDSWVYPNTSKPEYARLSRKYIYSLYWSTLTLTTIG
Ictidomys_tride LYTLIIIHWNACIYFAISKFIGFGTDSWVYPNTSKPEYARLSRKYIYSLYWSTLTLTTIG
Urocitellus_par LYTLIIIHWNACIYFAISKFIGFGTDSWVYPNTSKPEYARLSRKYIYSLYWSTLTLTTIG
Sciurus_vulgari LYILIIIHWNACIYFAISKFIGFGTDSWVYPNTSKPEYARLSRKYIYSLYWSTLTLTTIG
Sciurus_carolin LYTLIIIHWNACIYFAISKFIGFGTDSWVYPNTSKPEYARLSRKYIYSLYWSTLTLTTIG
Sciurus_niger@C LYTLIIIHWNACIYFAISKFIGFGTDSWVYPNTSKPEYARLSRKYIYSLYWSTLTLTTIG
                ** *********************************************************

Cnga3           ETPPPVKDEEYLFVVIDFLVGILIFATIVGNVGSMISNMNAPRVEFQAKIDSVKQYMQFR
Aplodontia_rufa ETPPPVKDEEYLFVVIDFLVGVLIFATIVGNVGSMISNMNAPRAEFQAKIDSVKQYMQFR
Xerus_inauris@C ETPPPVKDEEYLFVVIDFLVGILIFATIVGNVGSMISNMNAPRVEFQAKIDSVKQYMQFR
Marmota_monax@C ETPPPVKDEEYLFVVIDFLVGILIFATIVGNVGSMISNMNAPRVEFQAKIDSVKQYMQFR
Spermophilus_da ETPPPVKDEEYLFVVIDFLVGILIFATIVGNVGSMISNMNAPRVEFQAKIDSVKQYMQFR
Ictidomys_tride ETPPPVKDEEYLFVVIDFLVGILIFATIVGNVGSMISNMNAPRVEFQAKIDSVKQYMQFR
Urocitellus_par ETPPPVKDEEYLFVVIDFLVGVLIFATIVGNVGSMISNMNAPRVEFQAKIDSVKQYMQFR
Sciurus_vulgari ETPPPVKDEEYLFVVIDFLVGVLIFATIVGNVGSMISNMNAPRVEFQAKIDSVKQYMQFR
Sciurus_carolin ETPPPVKDEEYLFVVIDFLVGVLIFATIVGNVGSMISNMNAPRVEFQAKIDSVKQYMQFR
Sciurus_niger@C ETPPPVKDEEYLFVVIDFLVGVLIFATIVGNVGSMISNMNAPRVEFQAKIDSVKQYMQFR
                *********************:*********************.****************

Cnga3           KVTKDLETRVIRWFDYLWANRKTVDEKEVLKNLPDKLKAEIAINVHLDTLKKVRIFQDCE
Aplodontia_rufa KVTKDLETRVIRWFDYLWANRKTVDEKEVLKNLPDKLKAEIAINVHLDTLKKVRIFQDCE
Xerus_inauris@C KVTKDLETRVIRWFDYLWANRKTVDEKEVLKNLPDKLKAEIAINVHLDTLKKVRIFQDCE
Marmota_monax@C KVTKDLETRVIRWFDYLWANRKTVDEKEVLKNLPDKLKAEIAINVHLDTLKKVRIFQDCE
Spermophilus_da KVTKDLETRVIRWFDYLWANRKTVDEKEVLKNLPDKLKAEIAINVHLDTLKKVRIFQDCE
Ictidomys_tride KVTKDLETRVIRWFDYLWANRKTVDEKEVLKNLPDKLKAEIAINVHLDTLKKVRIFQDCE
Urocitellus_par KVTKDLETRVIRWFDYLWANRKTVDEKEVLKNLPDKLKAEIAINVHLDTLKKVRIFQDCE
Sciurus_vulgari KVTKDLETRVIRWFDYLWANRKTVDEKEVLKNLPDKLKAEIAINVHLDTLKKVRIFQDCE
Sciurus_carolin KVTKDLETRVIRWFDYLWANRKTVDEKEVLKNLPDKLKAEIAINVHLDTLKKVRIFQDCE
Sciurus_niger@C KVTKDLETRVIRWFDYLWANRKTVDEKEVLKNLPDKLKAEIAINVHLDTLKKVRIFQDCE
                ************************************************************

Cnga3           AGLLVELVLKLRPTVFSPGDYICKKGDIGREMYIIKEGKLAVVADDGVTQFVVLSDGSYF
Aplodontia_rufa AGLLVELVLKLRPTVFSPGDYICKKGDIGREMYIIKEGKLAVVADDGVTQFVVLSDGSYF
Xerus_inauris@C AGLLVELVLKLRPTVFSPGDYICKKGDIGREMYIIKEGKLAVVADDGVTQFVVLSDGSYF
Marmota_monax@C AGLLVELVLKLRPTVFSPGDYICKKGDIGREMYIIKEGKLAVVADDGVTQFVVLSDGSYF
Spermophilus_da AGLLVELVLKLRPTVFSPGDYICKKGDIGREMYIIKEGKLAVVADDGVTQFVVLSDGSYF
Ictidomys_tride AGLLVELVLKLRPTVFSPGDYICKKGDIGREMYIIKEGKLAVVADDGVTQFVVLSDGSYF
Urocitellus_par AGLLVELVLKLRPTVFSPGDYICKKGDIGREMYIIKEGKLAVVADDGVTQFVVLSDGSYF
Sciurus_vulgari AGLLVELVLKLRPTVFSPGDYICKKGDIGREMYIIKEGKLAVVADDGVTQFVVLSDGSYF
Sciurus_carolin AGLLVELVLKLRPTVFSPGDYICKKGDIGREMYIIKEGKLAVVADDGVTQFVVLSDGSYF
Sciurus_niger@C AGLLVELVLKLRPTVFSPGDYICKKGDIGREMYIIKEGKLAVVADDGVTQFVVLSDGSYF
                ************************************************************

Cnga3           GEISILNIKGSKSGNRRTANIRSIGYSDLFCLSKDDLMEALTEYPDAKRALEEKGRQILM
Aplodontia_rufa GEISILNIKGSKSGNRRTANIRSIGYSDLFCLSKDDLMEALTEYPDAKRALEEKGRQILM
Xerus_inauris@C GEISILNIKGSKSGNRRTANIRSIGYSDLFCLSKDDLMEALTEYPDAKRALEEKGRQILM
Marmota_monax@C GEISILNIKGSKSGNRRTANIRSIGYSDLFCLSKDDLMEALTEYPDAKRALEEKGRQILM
Spermophilus_da GEISILNIKGSKSGNRRTANIRSIGYSDLFCLSKDDLMEALTEYPDAKRALEEKGRQILM
Ictidomys_tride GEISILNIKGSKSGNRRTANIRSIGYSDLFCLSKDDLMEALTEYPDAKRALEEKGRQILM
Urocitellus_par GEISILNIKGSKSGNRRTANIRSIGYSDLFCLSKDDLMEALTEYPDAKRALEEKGRQILM
Sciurus_vulgari GEISILNIKGSKSGNRRTANIRSIGYSDLFCLSKDDLMEALTEYPDAKRALEEKGRQILM
Sciurus_carolin GEISILNIKGSKSGNRRTANIRSIGYSDLFCLSKDDLMEALTEYPDAKRALEEKGRQILM
```

```
Sciurus_niger@C GEISILNIKGSKSGNRRTANIRSIGYSDLFCLSKDDLMEALTEYPDAKRALEEKGRQILM
                ************************************************************

Cnga3           KDNLIDEDLVAARVDTRDVEEKVEYLESSLDILQTRFARLLAEYSASQMKLKQRLTRLES
Aplodontia_rufa KDNLIDEDLVAARVDTRDVEEKVEYLESSLDILQTRFARLLAEYSASQMKLKQRLTRLES
Xerus_inauris@C KDNLIDEDLVAARVDTRDVEEKVEYLESSLDILQTRFARLLAEYSASQMKLKQRLTRLES
Marmota_monax@C KDNLIDEDLVAARVDTRDVEEKVEYLESSLDILQTRFARLLAEYSASQMKLKQRLTRLES
Spermophilus_da KDNLIDEDLVAARVDTRDVEEKVEYLESSLDILQTRFARLLAEYSASQMKLKQRLTRLES
Ictidomys_tride KDNLIDEDLVAARVDTRDVEEKVEYLESSLDILQTRFARLLAEYSASQMKLKQRLTRLES
Urocitellus_par KDNLIDEDLVAARVDTRDVEEKVEYLESSLDILQTRFARLLAEYSASQMKLKQRLTRLES
Sciurus_vulgari KDNLIDEDLVAARVDTRDVEEKVEYLESSLDILQTRFARLLAEYSASQMKLKQRLTRLES
Sciurus_carolin KDNLIDEDLVAARVDTRDVEEKVEYLESSLDILQTRFARLLAEYSASQMKLKQRLTRLES
Sciurus_niger@C KDNLIDEDLVAARVDTRDVEEKVEYLESSLDILQTRFARLLAEYSASQMKLKQRLTRLES
                ************************************************************

Cnga3           QMNRRCCGFSPDRENSEDASKTDZ
Aplodontia_rufa QMKRRCCGFLSDGENSEDAAKTDQ
Xerus_inauris@C QMKRRCCGFSSDRENSEEAAKTEQ
Marmota_monax@C QMKRRCCGFSSDRENSEEAAKTEE
Spermophilus_da QMKRRCCGFSSDRENSEEAAKTEE
Ictidomys_tride QMKRRCCGFSSDRENSEEAAKTEE
Urocitellus_par QMKRRCCGFSSDRENSEEAAKTEE
Sciurus_vulgari QMKRRCCDFSSDGENSEEAAKTEQ
Sciurus_carolin QMKRRCCDFSSDGENSEEAAKTEQ
Sciurus_niger@C QMKRRCCGFSSDGENSEEAAKTEQ
                **:****.* .* ****:*:**:
```

GNAT2 encodes G protein subunit alpha transducin 2. Diseases associated with *GNAT2* include achromatopsia 4 and achromatopsia. Among its related pathways are ERK signaling and signaling by GPCR. Gene ontology (GO) annotations related to this gene include GTP binding and obsolete signal transducer activity. An important paralog of this gene is *GNAT1*. Transducin is a 3-subunit guanine nucleotide-binding protein (G protein) which stimulates the coupling of rhodopsin and cGMP-phoshodiesterase during visual impulses. The transducin alpha subunits in rods and cones are encoded by separate genes. This gene encodes the alpha subunit in cones.

Among 354 amino acids for which sequence data are available, 351 are identical among all species. Among *Sciurus* sp., *S. carolinensis* has a phenylalanine instead of a tyrosine at position 146.

```
Gnat2           MGSGISAEDKELARRSKELEKKLQEDADKEAKTVKLLLLGAGESGKSTIVKQMKIIHQDG
Aplodontia_rufa MGSGISAEDKELARRSKELEKKLQEDADKEARTVKLLLLGAGESGKSTIVKQMKIIHQDG
Xerus_inauris@G MGSGISAEDKELARRSKELEKKLQEDADKEAKTVKLLLLGAGESGKSTIVKQMKIIHQDG
Marmota_monax@G MGSGISAEDKELARRSKELEKKLQEDADKEAKTVKLLLLGAGESGKSTIVKQMKIIHQDG
Spermophilus_da MGSGISAEDKELARRSKELEKKLQEDADKEAKTVKLLLLGAGESGKSTIVKQMKIIHQDG
Ictidomys_tride MGSGISAEDKELARRSKELEKKLQEDADKEAKTVKLLLLGAGESGKSTIVKQMKIIHQDG
Urocitellus_par MGSGISAEDKELARRSKELEKKLQEDADKEAKTVKLLLLGAGESGKSTIVKQMKIIHQDG
Sciurus_vulgari MGSGISAEDKELARRSKELEKKLQEDADKEAKTVKLLLLGAGESGKSTIVKQMKIIHQDG
Sciurus_carolin MGSGISAEDKELARRSKELEKKLQEDADKEAKTVKLLLLGAGESGKSTIVKQMKIIHQDG
Sciurus_niger@G MGSGISAEDKELARRSKELEKKLQEDADKEAKTVKLLLLGAGESGKSTIVKQMKIIHQDG
                *******************************:****************************

Gnat2           YSPEECLEFKSVIYGNVLQSILAIIRAMSTLGIDYAEPSCADAGRQLNNLADSTEEGTMP
Aplodontia_rufa YSPEECLEFKSVIYGNVLQSILAIIRAMSTLGIDYAEPSCADDGRQLNNLADSTEEGTMP
Xerus_inauris@G YSPEECLEFKSVIYGNVLQSILAIIRAMSTLGIDYAEPSCADAGRQLNNLADSTEEGTMP
Marmota_monax@G YSPEECLEFKSVIYGNVLQSILAIIRAMSTLGIDYAEPSCADAGRQLNNLADSTEEGTMP
```

```
Spermophilus_da YSPEECLEFKSVIYGNVLQSILAIIRAMSTLGIDYAEPSCADAGRQLNNLADSTEEGTMP
Ictidomys_tride YSPEECLEFKSVIYGNVLQSILAIIRAMSTLGIDYAEPSCADAGRQLNNLADSTEEGTMP
Urocitellus_par YSPEECLEFKSVIYGNVLQSILAIIRAMSTLGIDYAEPSCADAGRQLNNLADSTEEGTMP
Sciurus_vulgari YSPEECLEFKSVIYGNVLQSILAIIRAMSTLGIDYAEPSCADAGRQLNNLADSTEEGTMP
Sciurus_carolin YSPEECLEFKSVIYGNVLQSILAIIRAMSTLGIDYAEPSCADAGRQLNNLADSTEEGTMP
Sciurus_niger@G YSPEECLEFKSVIYGNVLQSILAIIRAMSTLGIDYAEPSCADDGRQLNNLADSTEEGTMP
                ****************************************** *****************

Gnat2           PELVDVIRKLWKDGGVQACFDRAAEFQLNDSASYYLNQLDRITDPNYLPNEQDVLRSRVK
Aplodontia_rufa PELVDVIRKLWKDGGVQACFDRAAEYQLNDSASYYLNQLDRITDPNYLPNEQDVLRSRVK
Xerus_inauris@G PELVDVIRKLWKDGGVQACFDRAAEFQLNDSASYYLNQLDRITDPNYLPNEQDVLRSRVK
Marmota_monax@G PELVDVIRKLWKDGGVQACFDRAAEYQLNDSASYYLNQLDRITDPNYLPNEQDVLRSRVK
Spermophilus_da PELVDVIRKLWKDGGVQACFDRAAEYQLNDSASYYLNQLDRITDPNYLPNEQDVLRSRVK
Ictidomys_tride PELVDVIRKLWKDGGVQACFDRAAEFQLNDSASYYLNQLDRITDPNYLPNEQDVLRSRVK
Urocitellus_par PELVDVIRKLWKDGGVQACFDRAAEYQLNDSASYYLNQLDRITDPNYLPNEQDVLRSRVK
Sciurus_vulgari PELVDVIRKLWKDGGVQACFDRAAEYQLNDSASYYLNQLDRITDPNYLPNEQDVLRSRVK
Sciurus_carolin PELVDVIRKLWKDGGVQACFDRAAEFQLNDSASYYLNQLDRITDPNYLPNEQDVLRSRVK
Sciurus_niger@G PELVDVIRKLWKDGGVQACFDRAAEYQLNDSASYYLNQLDRITDPNYLPNEQDVLRSRVK
                *************************:**********************************

Gnat2           TTGIIETKFSVKDLNFRMFDVGGQRSERKKWIHCFEGVTCIIFCAALSAYDMVLVEDDEV
Aplodontia_rufa TTGIIETKFSVKDLNFRMFDVGGQRSERKKWIHCFEGVTCIIFCAALSAYDMVLVEDDEV
Xerus_inauris@G TTGIIETKFSVKDLNFRMFDVGGQRSERKKWIHCFEGVTCIIFCAALSAYDMVLVEDDEV
Marmota_monax@G TTGIIETKFSVKDLNFRMFDVGGQRSERKKWIHCFEGVTCIIFCAALSAYDMVLVEDDEV
Spermophilus_da TTGIIETKFSVKDLNFRMFDVGGQRSERKKWIHCFEGVTCIIFCAALSAYDMVLVEDDEV
Ictidomys_tride TTGIIETKFSVKDLNFRMFDVGGQRSERKKWIHCFEGVTCIIFCAALSAYDMVLVEDDEV
Urocitellus_par TTGIIETKFSVKDLNFRMFDVGGQRSERKKWIHCFEGVTCIIFCAALSAYDMVLVEDDEV
Sciurus_vulgari TTGIIETKFSVKDLNFRMFDVGGQRSERKKWIHCFEGVTCIIFCAALSAYDMVLVEDDEV
Sciurus_carolin TTGIIETKFSVKDLNFRMFDVGGQRSERKKWIHCFEGVTCIIFCAALSAYDMVLVEDDEV
Sciurus_niger@G TTGIIETKFSVKDLNFRMFDVGGQRSERKKWIHCFEGVTCIIFCAALSAYDMVLVEDDEV
                ************************************************************

Gnat2           NRMHESLHLFNSICNHKFFAATSIVLFLNKKDLFEEKIKKVHLSICFPEYDGNNSYEDAG
Aplodontia_rufa NRMHESLHLFNSICNHKFFAATSIVLFLNKKDLFEEKIKKVHLSICFPEYDGNNSYEDAG
Xerus_inauris@G NRMHESLHLFNSICNHKFFAATSIVLFLNKKDLFEEKIKKVHLSICFPEYDGNNSYEDAG
Marmota_monax@G NRMHESLHLFNSICNHKFFAATSIVLFLNKKDLFEEKIKKVHLSICFPEYDGNNSYEDAG
Spermophilus_da NRMHESLHLFNSICNHKFFAATSIVLFLNKKDLFEEKIKKVHLSICFPEYDGNNSYEDAG
Ictidomys_tride NRMHESLHLFNSICNHKFFAATSIVLFLNKKDLFEEKIKKVHLSICFPEYDGNNSYEDAG
Urocitellus_par NRMHESLHLFNSICNHKFFAATSIVLFLNKKDLFEEKIKKVHLSICFPEYDGNNSYEDAG
Sciurus_vulgari NRMHESLHLFNSICNHKFFAATSIVLFLNKKDLFEEKIKKVHLSICFPEYDGNNSYEDAG
Sciurus_carolin NRMHESLHLFNSICNHKFFAATSIVLFLNKKDLFEEKIKKVHLSICFPEYDGNNSYEDAG
Sciurus_niger@G NRMHESLHLFNSICNHKFFAATSIVLFLNKKDLFEEKIKKVHLSICFPEYDGNNSYEDAG
                ************************************************************

Gnat2           NYIKSQFLDLNMRKDVKEIYSHMTCATDTQNVKFVFDAVTDIIIKENLKDCGLFZ
Aplodontia_rufa NYIKSQFLDLNMRKDVKEIYSHMTCATDTQNVKFVFDAVTDIIIKENLKDCGLF-
Xerus_inauris@G NYIKSQFLDLNMRKDVKEIYSHMTCATDTQNVKFVFDAVTDIIIKENLKDCGLF-
Marmota_monax@G NYIKSQFLDLNMRKDVKEIYSHMTCATDTQNVKFVFDAVTDIIIKENLKDCGLF-
Spermophilus_da NYIKSQFLDLNMRKDVKEIYSHMTCATDTQNVKFVFDAVTDIIIKENLKDCGLF-
Ictidomys_tride NYIKSQFLDLNMRKDVKEIYSHMTCATDTQNVKFVFDAVTDIIIKENLKDCGLF-
Urocitellus_par NYIKSQFLDLNMRKDVKEIYSHMTCATDTQNVKFVFDAVTDIIIKENLKDCGLF-
Sciurus_vulgari NYIKSQFLDLNMRKDVKEIYSHMTCATDTQNVKFVFDAVTDIIIKENLKDCGLF-
Sciurus_carolin NYIKSQFLDLNMRKDVKEIYSHMTCATDTQNVKFVFDAVTDIIIKENLKDCGLF-
Sciurus_niger@G NYIKSQFLDLNMRKDVKEIYSHMTCATDTQNVKFVFDAVTDIIIKENLKDCGLF-
                ******************************************************
```

OPN1MW encodes opsin 1, medium wave sensitive. Diseases associated with *OPN1MW* include Deutan series partial colorblindness and blue cone monochromacy. Among its related pathways are signaling by GPCR and peptide ligand-binding receptors. Gene ontology (GO) annotations related to this gene include G protein-coupled receptor activity and photoreceptor activity. An important paralog of this gene is *OPN1MW2*. This gene encodes for a light-absorbing visual pigment of the opsin gene family. The encoded protein is called green cone photopigment or medium-wavelength sensitive opsin. Opsins are G-protein coupled receptors with seven transmembrane domains, an N-terminal extracellular domain, and a C-terminal cytoplasmic domain. The long-wavelength opsin gene and multiple copies of the medium-wavelength opsin gene are tandemly arrayed on the X chromosome, and frequent unequal recombination and gene conversion may occur between these sequences. X chromosomes may have fusions of the medium- and long-wavelength opsin genes or may have more than one copy of these genes.

The amino acid sequence of OPN1MW is highly conserved among all species. The four varying positions occur at the end of the sequence alignment, and differentiate *S. vulgaris* OPN1MW from all other species for which we have sequence information.

```
Opn1mw          MAQRLTGEQTLD-HYEDSTHASIFTYTNSNSTKGPFEGPNYHIAPRWVYHLTSTWMILVV
Aplodontia_rufa ------GEQTLD-HYEDSTHASIFTYTNSNSTKGPFEGPNYHIAPRWVYHLTSTWMILVV
Xerus_inauris@O ------GEEGLETTWPDSTHASIFTYTNSNSTKGPFEGPNYHIAPRWVYHLTSTWMILVV
Marmota_monax@O ------GEEGLETTWPDSTHASIFTYTNSNSTKGPFEGPNYHIAPRWVYHLTSTWMILVV
Spermophilus_da ----------------NSTEASIFTYTNSNSTKGPFEGPNYHIAPRWVYHLTSTWMILVV
Ictidomys_tride ------GEEGLETTWPDSTHASIFTYTNSNSTKGPFEGPNYHIAPRWVYHLTSTWMILVV
Urocitellus_par --RRRNGEQTLD-HYEDSTHASIFTYTNSNSTKGPFEGPNYHIAPRWVYHLTSTWMILVV
Sciurus_vulgari ----------------NSTEASIFTYTNSNSTKGPFEGPNYHIAPRWVYHLTSTWMILVV
Sciurus_carolin ----------------NSTEASIFTYTNSNSTKGPFEGPNYHIAPRWVYHLTSTWMILVV
Sciurus_niger@O ----------------NSTEASIFTYTNSNSTKGPFEGPNYHIAPRWVYHLTSTWMILVV
                                :**.****************************************

Opn1mw          VASVFTNGLVLAATMRFKKLRHPLNWILVNLAVADLAETIIASTISVVNQIYGYFVLGHP
Aplodontia_rufa VASVFTNGLVLAATMRFKKLRHPLNWILVNLAVADLAETIIASTISVVNQIYGYFVLGHP
Xerus_inauris@O VASVFTNGLVLAATMRFKKLRHPLNWILVNLAVADLAETIIASTISVVNQIYGYFVLGHP
Marmota_monax@O VASVFTNGLVLAATMRFKKLRHPLNWILVNLAVADLAETIIASTISVVNQIYGYFVLGHP
Spermophilus_da VASVFTNGLVLAATMRFKKLRHPLNWILVNLAVADLAETIIASTISVVNQIYGYFVLGHP
Ictidomys_tride VASVFTNGLVLAATMRFKKLRHPLNWILVNLAVADLAETIIASTISVVNQIYGYFVLGHP
Urocitellus_par VASVFTNGLVLAATMRFKKLRHPLNWILVNLAVADLAETIIASTISVVNQIYGYFVLGHP
Sciurus_vulgari VASVFTNGLVLAATMRFKKLRHPLNWILVNLAVADLAETIIASTISVVNQIYGYFVLGHP
Sciurus_carolin VASVFTNGLVLAATMRFKKLRHPLNWILVNLAVADLAETIIASTISVVNQIYGYFVLGHP
Sciurus_niger@O VASVFTNGLVLAATMRFKKLRHPLNWILVNLAVADLAETIIASTISVVNQIYGYFVLGHP
                ************************************************************

Opn1mw          LCVIEGYIVSLCGITGLWSLAIISWERWLVVCKPFGNVRFDAKLATVGIVFSWVWAAIWT
Aplodontia_rufa LCVIEGYIVSLCGITGLWSLAIISWERWLVVCKPFGNVRFDAKLATVGIVFSWVWAAIWT
Xerus_inauris@O LCVIEGYIVSLCGITGLWSLAIISWERWLVVCKPFGNVRFDAKLATVGIVFSWVWAAIWT
Marmota_monax@O LCVIEGYIVSLCGITGLWSLAIISWERWLVVCKPFGNVRFDAKLATVGIVFSWVWAAIWT
Spermophilus_da LCVIEGYIVSLCGITGLWSLAIISWERWLVVCKPFGNVRFDAKLATVGIVFSWVWAAIWT
Ictidomys_tride LCVIEGYIVSLCGITGLWSLAIISWERWLVVCKPFGNVRFDAKLATVGIVFSWVWAAIWT
Urocitellus_par LCVIEGYIVSLCGITGLWSLAIISWERWLVVCKPFGNVRFDAKLATVGIVFSWVWAAIWT
Sciurus_vulgari LCVIEGYIVSLCGITGLWSLAIISWERWLVVCKPFGNVRFDAKLATVGIVFSWVWAAIWT
Sciurus_carolin LCVIEGYIVSLCGITGLWSLAIISWERWLVVCKPFGNVRFDAKLATVGIVFSWVWAAIWT
Sciurus_niger@O LCVIEGYIVSLCGITGLWSLAIISWERWLVVCKPFGNVRFDAKLATVGIVFSWVWAAIWT
                ************************************************************
```

```
Opn1mw           APPIFGWSRYWPYGLKTSCGPDVFSGTSYPGVQSYMMVLMVTCCIFPLSIIVLCYLQVWL
Aplodontia_rufa  APPIFGWSRYWPYGLKTSCGPDVFSGTSYPGVQSYMMVLMVTCCIFPLSIIVLCYLQVWL
Xerus_inauris@O  APPIFGWSRYWPYGLKTSCGPDVFSGTSYPGVQSYMMVLMVTCCIFPLSIIVLCYLQVWL
Marmota_monax@O  APPIFGWSRYWPYGLKTSCGPDVFSGTSYPGVQSYMMVLMVTCCIFPLSIIVLCYLQVWL
Spermophilus_da  APPIFGWSRYWPYGLKTSCGPDVFSGTSYPGVQSYMMVLMVTCCIIPLSIIVLCYLQVWL
Ictidomys_tride  APPIFGWSRYWPYGLKTSCGPDVFSGTSYPGVQSYMMVLMVTCCIFPLSIIVLCYLQVWL
Urocitellus_par  APPIFGWSRYWPYGLKTSCGPDVFSGTSYPGVQSYMMVLMVTCCIFPLSIIVLCYLQVWL
Sciurus_vulgari  APPIFGWSRYWPYGLKTSCGPDVFSGTSYPGVQSYMMVLMVTCCIIPLSIIVLCYLQVWL
Sciurus_carolin  APPIFGWSRYWPYGLKTSCGPDVFSGTSYPGVQSYMMVLMVTCCIIPLSIIVLCYLQVWL
Sciurus_niger@O  APPIFGWSRYWPYGLKTSCGPDVFSGTSYPGVQSYMMVLMVTCCIFPLSIIVLCYLQVWL
                 *********************************************:**************

Opn1mw           AIRAVAKQQKESESTQKAEKEVTRMVVVMVFAYCLCWGPYTFFACFATAHPGYAFHPLVA
Aplodontia_rufa  AIRAVAKQQKESESTQKAEKEVTRMVVVMVFAYCLCWGPYTFFACFATAHPGYAFHPLVA
Xerus_inauris@O  AIRAVAKQQKESESTQKAEKEVTRMVVVMVFAYCLCWGPYTFFACFATAHPGYAFHPLVA
Marmota_monax@O  AIRAVAKQQKESESTQKAEKEVTRMVVVMVFAYCLCWGPYTFFACFATAHPGYAFHPLVA
Spermophilus_da  AIRAVAKQQKESESTQKAEKEVTRMVVVMVFAYCLCWGPYTFFACFATAHPGYAFHPLVA
Ictidomys_tride  AIRAVAKQQKESESTQKAEKEVTRMVVVMVFAYCLCWGPYTFFACFATAHPGYAFHPLVA
Urocitellus_par  AIRAVAKQQKESESTQKAEKEVTRMVVVMVFAYCLCWGPYTFFACFATAHPGYAFHPLVA
Sciurus_vulgari  AIRAVAKQQKESESTQKAEKEVTRMVVVMVFAYCLCWGPYTFFACFATAHPGYAFHPLVA
Sciurus_carolin  AIRAVAKQQKESESTQKAEKEVTRMVVVMVFAYCLCWGPYTFFACFATAHPGYAFHPLVA
Sciurus_niger@O  AIRAVAKQQKESESTQKAEKEVTRMVVVMVFAYCLCWGPYTFFACFATAHPGYAFHPLVA
                 ************************************************************

Opn1mw           SLPSYFAKSATIYNPIIYVFMNRQFRNCI-LHLFGKKVDDSSELSSTSKTEVSSVSSVSP
Aplodontia_rufa  SLPSYFAKSATIYNPIIYVFMNRQFRNAL-LHLLGK------------------------
Xerus_inauris@O  SLPSYFAKSATIYNPIIYVFMNRQFRNCI-LHLFGK------------------------
Marmota_monax@O  SLPSYFAKSATIYNPIIYVFMNRQFRNAI-------------------------------
Spermophilus_da  SLPSYFAKSATIYNPIIYVFMNRQFRNAI-------------------------------
Ictidomys_tride  SLPSYFAKSATIYNPIIYVFMNRQFRNAI-------------------------------
Urocitellus_par  SLPSYFAKSATIYNPIIYVFMNRQFRNAI-------------------------------
Sciurus_vulgari  SLPSYFAKSATIYNPIIYVFMNRQFRNAIKMVLLGK------------------------
Sciurus_carolin  SLPSYFAKSATIYNPIIYVFMNRQFRNCI-LHLLGK------------------------
Sciurus_niger@O  SLPSYFAKSATIYNPIIYVFMNRQFRNCI-LHLLGK------------------------
                 ***************************.:
```

PDE6C encodes phosphodiesterase 6C. Diseases associated with *PDE6C* include cone dystrophy 4 and achromatopsia. Among its related pathways are metabolism and metabolism of nucleotides. Gene ontology (GO) annotations related to this gene include 3',5'-cyclic-nucleotide phosphodiesterase activity and 3',5'-cyclic-GMP phosphodiesterase activity. An important paralog of this gene is *PDE6B*. This gene encodes the alpha-prime subunit of cone phosphodiesterase, which is composed of a homodimer of two alpha-prime subunits and 3 smaller proteins of 11, 13, and 15 kDa.

Along the 936-amino acid PDE6C sequence alignment, some upstream regions are well-conserved, while other, downstream regions are highly variable. There are 60 point-differences among sciurids, including tracts of 7, 7, 6, and 6 contiguous amino acids. *A. rufa* has a deletion of 27 amino acids at points 281-315, and an insertion of 29 amino acids at positions 363-391. *M. monax* and *S. vulgaris* have an insertion of 27 or 28 amino acids at positions 774-801. Six species have and three species lack an indel of 32 or 33 amino acids at positions 847-880.

```
Pde6c            MGEISQEAVERYLEKNPCFAKEYFDKKLRVEALGVIFKNSHAGVQTGLSLPEMTQVEESA
Aplodontia_rufa  MGEISQEAVERYLEKNPCFAKEYFDKKLRVEALGVIFKNSHAGVQTGLSLPELTQVEESA
```

```
Xerus_inauris@P MGEISQEAVERYLEKNPCFAKEYFDKKLRVEALGVIFKNSHAGVQTSLSLPELTQVEESA
Marmota_monax@P MGEISQEAVERYLEKNPCFAKEYFDKKLRVEALGVIFKNSHAGVQTGLSLPELTQVEESA
Spermophilus_da MGEISQEAVERYLEKNPCFAKEYFDKKLRVEALGVIFKNSHAGVQTGLSLPELTQVEESA
Ictidomys_tride MGEISQEAVERYLEKNPCFAKEYFDKKLRVEALGVIFKNSHAGVQTGLSLPELTQVEESA
Urocitellus_par MGEISQEAVERYLEKNPCFAKEYFDKKLRVEALGVIFKNSHAGVQTGLSLPELTQVEESA
Sciurus_vulgari MGEISQEAVERYLEKNPCFAKEYFDKKLRVEALGVIFKNSHAGVQTGLSLPELTQVEESA
Sciurus_carolin MGEISQEAVERYLEKNPCFAKEYFDKKLRVEALGVIFKNSHAGVQTGLSLPELTQVEESA
Sciurus_niger@P MGEISQEAVERYLEKNPCFAKEYFDKKLRVEALGVIFKNSHAGVQTGLSLPELTQVEESA
                **********************************************.*****:*******

Pde6c           VCLELLQCMQDEAGSAEQMAHRALQRLAQLLQADCCSMFSCRARNGIPEVASRLLNVTPT
Aplodontia_rufa VCLELLQCMQDEAGSAEQMAHRALQRLAQLLQADRCSMFSCRARNGIPEVASRLLNVTPT
Xerus_inauris@P VCLELLQCMQDEAGSAEQMAHRALQRLAQLLQADRCSMFSCRARNGIPEVASRLLNVTPT
Marmota_monax@P VCLELLQCMQDEAGSAEQMAHRALQRLAQLLQADRCSMFSCRARNGIPEVASRLLNVTPT
Spermophilus_da VCLELLQCMQDEAGSAEQMAHRALQRLAQLLQADRCSMFSCRARNGIPEVASRLLNVTPT
Ictidomys_tride VCLELLQCMQDEAGSAEQMAHRALQRLAQLLQADRCSMFSCRARNGIPEVASRLLNVTPT
Urocitellus_par VCLELLQCMQDEAGSAEQMAHRALQRLAQLLQADRCSMFSCRARNGIPEVASRLLNVTPT
Sciurus_vulgari VCLELLQCMQDEAGSAEQMAHRALQRLAQLLQADCCSMFSCRARNGIPEVASRLLNVTPT
Sciurus_carolin VCLELLQCMQDEAGSAEQMAHRALQRLAQLLQADRCSMFSCRARNGIPEVASRLLNVTPT
Sciurus_niger@P VCLELLQCMQDEAGSAEQMAHRALQRLAQLLQADRCSMFSCRARNGIPEVASRLLNVTPT
                ********************************** *************************

Pde6c           SKFEDNLVAPDREVVFPLDIGIVGWVAHVKKALNVSDVKKNSHFSDFMDKQTGYVTRNLL
Aplodontia_rufa SKFEDNLVAPDREVVFPLDIGIVGWVAHVKKALNVSDVKKNSHFSDFMDKQTGYVTKSLL
Xerus_inauris@P SKFEDNLVAPDREVVFPLDIGIVGWVAHVKKALNVPDVKKNSHFSDVMDKQTGYVTKSLM
Marmota_monax@P SKFEDNLVAPDREVVFPLDIGIVGWVAHVKKALNVPDVKKNSHFSDFMDKQTGYVTRNLL
Spermophilus_da SKFEDNLVAPDREVVFPLDIGIVGWVAHVKKALNVPDVKKNSHFSDFMDKQTGYVTRNLL
Ictidomys_tride SKFEDNLVAPDREVVFPLDIGIVGWVAHVKKALNVSDVKKNSHFSDFMDKQTGYVTRNLL
Urocitellus_par SKFEDNLVAPDREVVFPLDIGIVGWVAHVKKALNVSDVKKNSHFSDFMDKQTGYVTRNLL
Sciurus_vulgari SKFEDNLVAPDREVVFPLDIGIVGWVAHVKKALNVPDVKKNSHFSDFMDKQTGYVTKSLL
Sciurus_carolin SKFEDNLVAPDREVVFPLDIGIVGWVAHVKKALNVPDVKKNSHFSDFMDKQTGYVTRNLL
Sciurus_niger@P SKFEDNLVAPDREVVFPLDIGIVGWVAHVKKALNVPDVKKNSHFSDFMDKQTGYVTRNLL
                ***********************************.**********.*********:.*:

Pde6c           AVPIVAGKEVLAVVMAVNKISAPEFSKQDEEVFSKYLSFVAVALRLQHTSYLYSVESRRS
Aplodontia_rufa AAPIVMGKEVLAVVMALNKLNASEFSKQDEEVFSKYLIFVSVILKLHHTNYLYNVEFRRS
Xerus_inauris@P AAPVVMGKEVLAVLMAVNKVNASEFSKQDEEVFTKYLNFVSVILRLHHTNYLYNVESRKS
Marmota_monax@P AVPVVMGKEVLAVLVAVNKVNASEFSKQDEEVFTKYLNFVSVILKLHHTNYLYSVESRRS
Spermophilus_da AAPVVMGKEVLAVLAAVNKVNASEFSKQDEEVMSKYLSFVAVALRLQHTSYLYSVESRRS
Ictidomys_tride AAPVVMGKEVLAVLAAVNKVNASEFSKQDEEVFTKYLNFVSVILKLHHTNYLYNIESRRS
Urocitellus_par AVPVVMGKEVLAVLAAVNKVNASEFSKQDEEVMLT----LHIHLEVQEIDLLYNVESRRS
Sciurus_vulgari AAPVVMGKEVLAVLMAVNKVNASEFSKQDEEVFTKYLNFVSVILKLHHTNYLYSVESRRS
Sciurus_carolin AVPIVMGKEVLAVLMAVNKVNASEFSKQDEEVFTKYLNFVSVILKLHHTSYLYSVESRRS
Sciurus_niger@P AVPIVMGKEVLAVLMAVNKVNASEFSKQDEEVFTKYLNFVSVALRLQHTSYLYSVESRRS
                *.*:* *******: *:**:.*.*********: .    : : *.::. . **.:* *:*

Pde6c           QILMWSANKVFEELTDVERQFHKALYTIRTYLNCDRYSIGLLDMTKEK------------
Aplodontia_rufa QILMWSANKVFEELTDVERQFHKALYTVRTYLNCDRYSIGLLDMTKEK------------
Xerus_inauris@P QILMWSANKVFEELTDVERQFHKALYTIRTYLNCDRYSIGLLDMTKEKVRPGALRGPPAR
Marmota_monax@P QILMWSANKVFEELTDVERQFHKALYTIRTYLNCDRYSIGLLDMTKEKVRSGALGSPPAQ
Spermophilus_da QILMWSANKVFEELTDVERQFHKALYTIRTYLNCDRYSIGLLDMTKEKVRSGALGSPPGQ
Ictidomys_tride QILMWSANKVFEELTDVERQFHKALYTIRTYLNCDRYSIGLLDMTKEKVRSGALGSPPAQ
Urocitellus_par QILMWSANKVFEELTDVERQFHKALYTIRTYLNCDRYSIGLLDMTKEKVRSGALGSPPAQ
Sciurus_vulgari QILMWSANKVFEELTDVERQFHKALYTIRTYLNCDRYSIGLLDMTKEKVRPGAFRGPPAQ
Sciurus_carolin QILMWSANKVFEELTDVERQFHKALYTIRTYLNCDRYSIGLLDMTKEKVRPGVFRGPPGQ
Sciurus_niger@P QILMWSANKVFEELTDVERQFHKALYTIRTYLNCDRYSIGLLDMTKEKVRPGVFRGPPGQ
```

```
                  ***************************:********************

Pde6c             ---------------EFYDEWPIKLGEVEPYKGPKTPDGREIIFYKIIDYILHGKEEINV
Aplodontia_rufa   ---------------EFFDVWPVLMGEAQPYSGPRTPDGREIIFYKVIDYILHGKEEIKV
Xerus_inauris@P   PDPAPGLPPLFSSAQEFFDVWPVLMGEAQPYSGPRTPDGREIIFYKIIDYILHGKEEINV
Marmota_monax@P   PDPAAGLSPLFSSAQEFFDVWPVLMGEAQPYSGPCTPDGREVIFYKVIDYILHGKEEIKV
Spermophilus_da   PDPAPGLSPLFSSAQEFFDVWPVLMGEAQPYSGPRTPDGREVIFYKVIDYILHGKEEIKV
Ictidomys_tride   PDPAPGLSPLFSSAQEFFDVWPVLMGEAQPYSGPRTPDGREVIFYKVIDYILHGKEEIKV
Urocitellus_par   PDPAPGLSPLFSSAQEFFDVWPVLMGEAQPYSGPRTPDGREVIFYKVIDYILHGKEEIKV
Sciurus_vulgari   PDPAPGLSILLSSAQEFFDVWPVLMGEAQPYTGPRTPDGREVIFYKVIDYILHGKEEIKV
Sciurus_carolin   PDPAPGLSALLSSAQEFFDVWPVLMGEAPPYSGPRTPDGREIIFYKIIDYILHGKEEINV
Sciurus_niger@P   PDPAPGLSALLSSAQEFFDVWPVLMGEAQPYSGPRTPDGREIIFYKIIDYILHGKEEINV
                                 **:* **: :**. **.** ******:****:***********:*

Pde6c             IP-----------------------------SPPADHWTLVSGLPTYVAENGFICNMLNA
Aplodontia_rufa   IPVLSSSSGVAIFLCTVHFSQQQFSFFSPSRTPPADHWTFVSGLPTYVAENGFVCNMLNA
Xerus_inauris@P   IP-----------------------------SPPADHWTLVSGLPTYVAENGFICNMLNA
Marmota_monax@P   IP-----------------------------TPPADHWTFVSGLPTYVAENGFVSYWTNS
Spermophilus_da   IP-----------------------------TPPADHWTFVSGLPTYVAENGFVSYWTNS
Ictidomys_tride   IP-----------------------------TPPADHWTFVSGLPTYVAENGFVSYWTNS
Urocitellus_par   IP-----------------------------TPPADHWTFVSGLPTYVAENGFVSYWTNS
Sciurus_vulgari   IP-----------------------------TPPADHWTFVSGLPTYVAENGFVCNMLNA
Sciurus_carolin   IP-----------------------------SPPADHWTLVSGLPTYVAENGFICNMLNA
Sciurus_niger@P   IP-----------------------------SPPADHWTLVSGLPTYVAENGFICNMLNA
                  **                             :*******:*************:.   *:

Pde6c             PADEYFTFQKGPVDETGWVIKNVLSLPIVNKKEDIVGVATFYNRKDGKPFDEHDEHITET
Aplodontia_rufa   PADEYFTLQKGPVDETGWVIKNVLSLPIVNKKEDIVGVATFYNRKDGKPFDEHDEHITET
Xerus_inauris@P   PPSALHAPQKGPVDETGWVIKNVLSLPIVNKKEDIVGVATFYNRKDGKPFDEHDEHITET
Marmota_monax@P   PADEFMPLQKGPVDETGWVIKNVLSLPIVNKKEDIVGVATFYNRKDGKPFDEHDEHITET
Spermophilus_da   PADEFMHLQKGPVDETGWVIKNVLSLPIVNKKEDIVGVATFYNRKDGKPFDEHDEHITET
Ictidomys_tride   PADEFMPLQKGPVDETGWVIKNVLSLPIVNKKEDIVGVATFYNRKDGKPFDEHDEHITET
Urocitellus_par   PADEFMPLQKGPVDETGWVIKNVLSLPIVNKKEDIVGVATFYNRKDGKPFDEHDEHITET
Sciurus_vulgari   PP--YFTFQKGPVDETGWVIKNVLSLPIVNKKEDIVGVATFYNRKDGKPFDEHDEHITET
Sciurus_carolin   PADEFLPPQKGPVDETGWVIKNVLSLPIVNKKEDIVGVATFYNRKDGKPFDEHDEHITET
Sciurus_niger@P   PADEFFTFQKGPVDETGWVIKNVLSLPIVNKKEDIVGVATFYNRKDGKPFDEHDEHITET
                  *.      ****************************************************

Pde6c             LTQFLGWSLLNTDTYERVNKLESRKDIAQEMVMNLTKATPDEISSILKFKEKLNVEVIEE
Aplodontia_rufa   LTQFLGWSLLNTDTYEKMNKLENRKDIAQEMVMNLTKATPDEISSILKFKEKLNVEVIDE
Xerus_inauris@P   LTQFLGWSLLNTDTYEKMNKLENRKDIAQEMLMNHTKATPDEIKSILKFKEKLNVEVIEE
Marmota_monax@P   LTQFLGWSLLNTDTYEKMNKLENRKDIAQEMLMNHTKATPDEIKSILKFKEKLNVEVIEE
Spermophilus_da   LTQFLGWSLLNTDTYEKMNKLENRKDIAQEMLMNHTKATPDEIKSILKFKEKLNVEVIEE
Ictidomys_tride   LTQFLGWSLLNTDTYEKMNKLENRKDIAQEMLMNHTKATPDEIKSILKFKEKLNVEVIEE
Urocitellus_par   LTQFLGWSLLNTDTYEKMNKLENRKDIAQEMLMNHTKATPDEIKSILKFKEKLNVEVIEE
Sciurus_vulgari   LTQFLGWSLLNTDTYEKMNKLENRKDIAQEMVMNHTKATPDEISSILVPHLHSTVPLCGE
Sciurus_carolin   LTQFLGWSLLNTDTYEKMNKLENRKDIAQEMLMNHTKATPDEIKSILKFKEKLNVEVLQQ
Sciurus_niger@P   LTQFLGWSLLNTDTYEKMNKLENRKDIAQEMLMNHTKATPDEIKSILKFKEKLNVEVIEE
                  ****************::****.********:** ********.***  : : .* :  :

Pde6c             CEERQLLAILKEDLPDPRTADLYEFCFSDFPITEHELVKCGLRLFLEINVVEKFKVPVEV
Aplodontia_rufa   CNQKNLSFQQKEDLPDPKAVDLYEFRFSDFPITEHELIKCGLRMFFEINVVEKFKVPVEV
Xerus_inauris@P   CEERQLLAILKEDLPDPQAVELYEFRFSDFPITEHELIKCGLRLFLEINVVEKFKVPVEV
Marmota_monax@P   CEERQLLAILKEDLPDPQAVELYEFRFSDFPITEHELIKCGLRMFFEINVVEKFKVPVEV
Spermophilus_da   CEERQLLAILKEDLPDPQAVELYEFRFSDFPITEHELIKCGLRMFFEINVVEKFKVPVEV
Ictidomys_tride   CEERQLLAILKEDLPDPQAVELYEFRFSDFPITEHELIKCGLRMFFEINVVEKFKVPVEV
```

```
Urocitellus_par CEERQLLAILKEDLPDPQAVELYEFRFSDFPITEHELIKCGLRMFFEINVVEKFKVPVEV
Sciurus_vulgari AGSP------KEDLPDPRTVDLYEFRFSDFPITEHELIKCGLRLFLEINVVEKFKVPVEV
Sciurus_carolin ASHNIITPISKEDLPDPRAADLYEFCFSDFPITEHELVKCGLRLFLEINVVEKFKVPVEV
Sciurus_niger@P CEERQLLAILKEDLPDPRAADLYEFCFSDFPITEHELIKCGLRLFLEINVVEKFKVPVEV
                .         *******::.:**** ***********:*****:*:**************

Pde6c           LTRWMYTVRKGYRPVTYHNWRHGFNVGQTMFTLLMTGRLKKYYTDLEAFAMLAAAFCHDI
Aplodontia_rufa LTRWMYTVRKGYRSVTYHNWRHGFNVGQTMFALLMTGRLKKYYTDLEAFAMLAAAFCHDI
Xerus_inauris@P LTRWMYTVRKGYRAVTYHNWRHGFNVGQTMFTLLMTGRLKKYYTDLEAFAMLAAAFCHDI
Marmota_monax@P LTRWMYTVRKGYRAVTYHNWRHGFNVGQTMFTLLMTGRLKKYYTDLEAFAMLAAAFCHDI
Spermophilus_da LTRWMYTVRKGYRPVTYHNWRHGFNVGQTMFTLLMTGRLKKYYTDLEAFAMLAAAFCHDI
Ictidomys_tride LTRWMYTVRKGYRAVTYHNWRHGFNVGQTMFTLLMTGRLKKYYTDLEAFAMLAAAFCHDI
Urocitellus_par LTRWMYTVRKGYRAVTYHNWRHGFNVGQTMFTLLMTGRLKKYYTDLEAFAMLAAAFCHDI
Sciurus_vulgari LTRWMYTVRKGYRAVTYHNWRHGFNVGQTMFTLLMTGRLKKYYTDLEAFAMLAAAFCHDI
Sciurus_carolin LTRWMYTVRKGYRPVTYHNWRHGFNVGQTMFTLLMTGRLKKYYTDLEAFAMLAAAFCHDI
Sciurus_niger@P LTRWMYTVRKGYRPVTYHNWRHGFNVGQTMFTLLMTGRLKKYYTDLEAFAMLAAAFCHDI
                *************.***************:******************************

Pde6c           DHRGTNNLYQMKSTSPLARLHG-TSILERHHLEYSKTL-LQDESLNIFQNLNKRQFETVI
Aplodontia_rufa DHRGTNNLYQMKSTSPLARLHG-TSILERHHLEYSKTL-LQDESLNIFQNLNKRQFETVI
Xerus_inauris@P DHRGTNNLYQMKSTNPLARLHG-SSILERHHLEFGKFL-LAEESLNIFQNLNKRQFETVI
Marmota_monax@P DHRGTNNLYQMKSTSPLAKLHG-SSILERHHLEFGKFL-LQDESLNIYQNLNRRQHEHVI
Spermophilus_da DHRGTNNLYQMKSTSPLAKLHG-SSILERHHLEFGKFL-LQDESLNIYQNLNRRQHEHVI
Ictidomys_tride DHRGTNNLYQMKSTSPLAKLHG-SSILERHHLEFGKFL-LQDESLNIYQNLNRRQHEHVI
Urocitellus_par DHRGTNNLYQMKSTSPLAKLHG-SSILERHHLEFGKFL-LAEESLNIFQNLNKRQFETVI
Sciurus_vulgari DHRGTNNLYQMK-VSPYAVLHG-TSILERHHLEYSKTL-LQDQSLNIYQNLNRRQHEHVI
Sciurus_carolin DHRGTNNLYQMKSTSPLARLYNDTAVLESHHAELAFQLTVRDESLNIFQNLNKRQFETVI
Sciurus_niger@P DHRGTNNLYQMKSTSPLARLYNDTAVLESHHAALAFQLTVRDESLNIFQNLNKRQFETVI
                ************ ..* * *:. :::** **   .  * : ::****:****:**.* **

Pde6c           HLFEVAIIATDLALYFKKRTMFQKIVDTCEQMQSEEETIKYVTSDPTKKEVIM-------
Aplodontia_rufa HLFEVAIIATDLALYFKKRTMFQKIVDTCEQMQSEEETVKYVTSDPTKKEIIM-------
Xerus_inauris@P HLFEVAIIATDVKLFSRKRTMFQKIVDACEQMQTEEEAIKYVTIDPTKKEIIM-------
Marmota_monax@P HLFEVAIIATDLALYFKKRTMFQKIVDTCEQMQSEEETIKYVTSDPTKKEIIMVVRMVFS
Spermophilus_da HLFEVAIIATDLALYFKKRTMFQKIVDTCEQMQSEEETIKYVTSDPTKKEIIM-------
Ictidomys_tride HLFEVAIIATDLALYFKKRTMFQKIVDTCEQMQSEEETIKYVTSDPTKKEIIM-------
Urocitellus_par HLRHLLLCLYMVKLFSRKRTMFQKIVDACEQMQTEEEAIKYVTIDPTKKEIIM-------
Sciurus_vulgari HLFEVAIIATDLALYFKKRTMFQKIVDTCEQMQSEEETIKYVTSDPTKKEIIMVVGMVLP
Sciurus_carolin HLFEVAIIATDLALYFKKRTMFQKIVDTCEQMQSEEETIKYVTSDPTKKEVIM-------
Sciurus_niger@P HLFEVAIIATDLALYFKKRTMFQKIVDTCEQMQSEEETIKYVTSDPTKKEVIM-------
                ** .: :    : *: :**********:*****:***::**** ******:**

Pde6c           ---------------------AMMMTACDLSAITKPWEVQSQVALLVANEFWEQGDLERT
Aplodontia_rufa ---------------------ALALQVW-LSAITKPWEVPLQVALLVANEFWEQGDLERI
Xerus_inauris@P ---------------------AMMMTACDLSAITKPWEVQSQVALLVANEFWEQGDLERT
Marmota_monax@P FGGKKVVCVFFYLTIFLFFLRAMMMTACDLSAITKPWEVQSQVALLVANEFWEQGDLERT
Spermophilus_da ---------------------AMMMTACDLSAITKPWEVQSQVALLVANEFWEQGDLERT
Ictidomys_tride ---------------------AMMMTACDLSAITKPWEVQSQVALLVANEFWEQGDLERT
Urocitellus_par ---------------------AMMMTACDLSAITKPWEVQSQVALLVANEFWEQGDLERT
Sciurus_vulgari FGQRKAVCNSI-LTIFLFFLRAMMMTACDLSAITKPWEVQSQVALLVASEFWEQGDLERT
Sciurus_carolin ---------------------AMMMTACDLSAITKPWEVQSQVALLVASEFWEQGDLERT
Sciurus_niger@P ---------------------AMMMTACDLSAITKPWEVQSQVALLVANEFWEQGDLERT
                                     *: : .  **********  *******.**********

Pde6c           VLQQQPI-----------------------------------PMMDRSKKDELPKLQVGFID
Aplodontia_rufa VLQQQPIVKTLENALSRNSYNQSILFIKCKEIILKGCFFFPMMDRNRKDELPKLQVGFID
```

```
Xerus_inauris@P VLQQQPIVKALENALLSKNY-ESVYFVRIGHYLKRICFSFPMMDRNKKDELPKLQVGFID
Marmota_monax@P VLQQQPIVKTLENALLSKNY-ESVYFVHIGNYLKRIYFSFPMMDRNKKDELPKLQVGFID
Spermophilus_da VLQQQPIVKTLENALLSKNY-KSVYFVHIGNYLKRIYLSFPMMDRNKKDELPKLQVGFID
Ictidomys_tride VLQQQPIVKTLENALLSKNY-KSVYFVHIGNYLKRIYFSFPMMDRNKKDELPKLQVGFID
Urocitellus_par VLQQQPI---------------------------------PMMDRNKKDELPKLQVGFID
Sciurus_vulgari VLQQQPIVKTLENALLSKNY-ESVYFGHIGNYLKRICFFFPMMDRNKKDELPKLQVGFID
Sciurus_carolin VLQQQPI---------------------------------PMMDRNKKDELPKLQVGFID
Sciurus_niger@P VLQQQPIV------------------------------MPMMDRNKKDELPKLQVGFID
                *******                                 *****.:*************

Pde6c           FVCTFVYKEFSRFHGEITPMLNGLQNNRVEWKSLAEEYEAKVKVTEEEAGKQEEEASDGK
Aplodontia_rufa FVCTFVYK----------------------------------------------------
Xerus_inauris@P FVCTFVYK----------------------------------------------------
Marmota_monax@P FVCTFVYK----------------------------------------------------
Spermophilus_da FVCTFVYK----------------------------------------------------
Ictidomys_tride FVCTFVYK----------------------------------------------------
Urocitellus_par FVCTFVYK----------------------------------------------------
Sciurus_vulgari FVCTFVYK----------------------------------------------------
Sciurus_carolin FVCTFVYKEFSRFHKEITPMLNGLQNNRVEWKSLADEYDAKMKV----------------
Sciurus_niger@P FVCTFVYKAWDTFVD--LPDLMHLDNNFKCWKGLDE------------------------
                ********
```

Literature Cited

El Moussawi Z, Boueiri M, Al-Haddad C. 2021. Gene therapy in color vision deficiency: A review. Inter Ophth. 41: 1917–1927.

Van Hooser SD, Nelson SB. 2006. The squirrel as a rodent model of the human visual system. Vis Neurosci. 23: 765-778.
