# Supplementary material for: A draft genome assembly for the eastern fox squirrel, Sciurus niger
Source: G3 (Bethesda). 2021 Sep 22;11(12):jkab315. doi: 10.1093/g3journal/jkab315 (PMC8664420; doi:10.1093/g3journal/jkab315)
Supplement: jkab315_Supplementary_File_S4 [file jkab315_supplementary_file_s4.pdf]

Supplemental File S4 – Hibernation-related Genes

Onset of hibernations is a highly heritable trait (h2 = 0.61-1.0) in thirteen-lined ground squirrel. A genome scan (Grabek *et al*. 2019) revealed two loci significantly and 12 loci suggestively linked to variation in this trait, including the three treated below.

CHCHD3 encodes coiled-coil-helix-coiled-coil-helix domain containing 3. Diseases associated with *CHCHD3* include optic atrophy 1. Among its related pathways are metabolism of proteins and mitochondrial protein import. Gene ontology (GO) annotations related to this gene include phosphatase binding and RNA polymerase II-specific DNA-binding transcription repressor activity. An important paralog of this gene is *CHCHD6*. The protein encoded by this gene is an inner mitochondrial membrane scaffold protein. Absence of the encoded protein affects the structural integrity of mitochondrial cristae and leads to reductions in ATP production, cell growth, and oxygen consumption. This protein is part of the mitochondrial contact site and cristae organizing system (MICOS). Several transcript variants encoding different isoforms have been found for this gene.

Within a 152-amino acid alignment for CHCHD3, there were 18 point-differences among sciurids, including a five-amino acid deletion in *S. vulgaris* at positions 52-56, a seven-amino acid deletion in *S. carolinensis* and *S. niger* at positions 57-63, and a two amino acids insertion in *S. carolinensis* at positions 148-149.

```
Chchd3          VSDEDLKRRVAEELALEQAKKESEHQRRLKQARDLERERAAANEQLTRAVLRERISSEEE
Aplodontia_rufa ---------------------------RLKQSRDLERERAAASEQLTRAILRERISSEEE
Xerus_inauris@C ---------------------------RLKQSRDLERERAAANEQLTRAVLRERISSEEE
Marmota_monax@C ---------------------------RLKQSRDLERERAAANEQLTRAVLRERISSEEE
Spermophilus_da -----------------------------KQSRDLERERAAANEQLTRAVLRERISSEEE
Ictidomys_tride SPEPKIKDNNCKEFWLRCRHNTS---RRLKQARDLERERAAANEQLTRAVLRERISSEEE
Urocitellus_par ---------------------------RLKQSRDLERERADANEQLTRAVLRERISSEEE
Sciurus_vulgari ---------------------------RLKQSRDLERERAAANEQLTRAVLRERISSEEE
Sciurus_carolin ---------------------------RLKQSRDLERERAAANEQLTRAVLRERISSEEE
Sciurus_niger@C ---------------------------RLKQSRDLERERAAANEQLTRAVLRERISSEEE
                                             **:******** *.******:**********

Chchd3          RMKAKHLDIEDKARQLEEKDRVMRKQDAFYKEQLARLEERSSEFYKVTTEEYQKAAEEVE
Aplodontia_rufa RAKAKHLDIEDKARQLEEKDRVMRKQDAFYKEQLARLEERCS-FYKVTTNQYQKAAEELE
Xerus_inauris@C RMKAKHL-----AKQLEEKDRVMRKQDAFYKEQLARLEERSSEFCKVTTEEYQKAAEEVE
Marmota_monax@C RMKAKHL-----ARQLEEKDRVMKKQDAFYKEQLARLEERSSEFYKVTPEEYQKLLKEWK
Spermophilus_da RMKAKHL-----ARQLEEKDRVMKKQDAFYKEQLARLEERSSEFCKVTTEEYQKAAEEVE
Ictidomys_tride RMKAKHLDIEDKAKQLEEKDRVMRKQDAFYKEQLARLEERSSEFYKVTTEEYQMAAEEVE
Urocitellus_par RMKAKHLD----ARQLEEKDRVMKKQDAFYKEQLARLEERSSEFYKVTTEEYQKAAEEVE
Sciurus_vulgari RMKAKHL-----AKHTKE-----KNQVAFYKEHLTELEERNSEFGKVSTELNQMLLKKWK
Sciurus_carolin RMKAKHL-----AKQTKEKNQGMKNQVAFYKEHLTELEERNSEFGKVSTELNQMLL----
Sciurus_niger@C RMKAKHL-----AKQTKEKNQGMKNQVAFYKEYLTELEERNSEFGKVSTELNQMLL----
                * *****     *:: :*     ::* ***** *:.**** * * **:.:  *

Chchd3          AKFKRYEYHPVCADLQTKILQCYRQNTQQTLSCSALASQYMHCVNHAKQSMLEK--GGZ
Aplodontia_rufa PKFKRYEYHPVCADLQTKILQCYRQNTQQTLSCSALANQYMHCVNHAKQGMLK------
Xerus_inauris@C AKFKRYEYHPVCADLQTKILQCYRQNTQQTLSCSALASQYMHCVNHAKQSMLEK--AG-
Marmota_monax@C QKFKRYEYHPVCADLQTKILQCYRQNTQQTLSCSALASQYMHCVNHAKQSMLEK--GG-
Spermophilus_da AKFKRYEYHPVCADLQTKILQCYRQNTQQTLSCSALASQYMHCVNHAKQSMLEK--GG-
Ictidomys_tride AKFKRYEYHPVCADLQTKILQCYRQNTQQTLSCSALASQYMHCVNHAKQSMLEK--GG-
```

```
Urocitellus_par AKFKRYEYHPVCADLQTKILQCYRQNTQQTLSCSALASQYMHCVNHAKQSMLEK--GG-
Sciurus_vulgari CS-KRYEYHPVCADLQTKILQCYRQNTHQTLSCSALANQYMHCVNHAKQSMLEK--GG-
Sciurus_carolin ---KRYEYHPVCADLQTKILQCYRQNTHQTLSCSALANQYMHCVNHAKQVSVQARPGG-
Sciurus_niger@C ---KRYEYHPVCADLQTKILQCYRQNTHQTLSCSALANQYMHCVNHAKQSMLEK--GG-
                   ************************:*********.***********  ::
```

EXOC4 encodes exocyst complex component 4. Diseases associated with *EXOC4* include type I Meckel syndrome and congenital myasthenic syndrome 4C associated with acetylcholine receptor deficiency. Among its related pathways are peptide hormone metabolism and vesicle-mediated transport. Gene ontology (GO) annotations related to this gene include PDZ domain binding and Ral GTPase binding. The protein encoded by this gene is a component of the exocyst complex, a multiple protein complex essential for targeting exocytic vesicles to specific docking sites on the plasma membrane. Though best characterized in yeast, the component proteins and functions of exocyst complex have been demonstrated to be highly conserved in higher eukaryotes. At least eight components of the exocyst complex, including this protein, are found to interact with the actin cytoskeletal remodeling and vesicle transport machinery. The complex is also essential for the biogenesis of epithelial cell surface polarity. Alternate transcriptional splice variants, encoding different isoforms, have been characterized.

A 955-amino acid sequence of EXOC4 showed long stretches of perfect or near-perfect conservation among all species at issue. Among sciurids, we observed point-mutations at positions 511, 601, and 894.

```
Exoc4           MAAEAAGGKYRSTVSKSKDPSGLLISVI-RTLSTSDDVEDRENEKGRLEEAYEKCDRDLD
Aplodontia_rufa ---------------------------AFTRTLSTSDDVEDRENEKGRLEEAYEKCDRDLD
Xerus_inauris@E ---------------------SLIYSVLTRTLSTSDDVEDRENEKGRLEEAYEKCDRDLD
Marmota_monax@E ---------------------SLIYSVFTRTLSTSDDVEDRENEKGRLEEAYEKCDRDLD
Spermophilus_da ---------------------SLIYSVFTRTLSTSDDVEDRENEKGRLEEAYEKCDRDLD
Ictidomys_tride ---------------------SLIYSVFTRTLSTSDDVEDRENEKGRLEEAYEKCDRDLD
Urocitellus_par ------------TFLSAKIVFSLIYSVFTRTLSTSDDVEDRENEKGRLEEAYEKCDRDLD
Sciurus_vulgari ---------------------SLIYSVFTRTLSTSDDVEDRENEKGRLEEAYEKCDRDLD
Sciurus_carolin ---------------------SLIYSVFTRTLSTSDDVEDRENEKGRLEEAYEKCDRDLD
Sciurus_niger@E ---------------------SLIYSVFTRTLSTSDDVEDRENEKGRLEEAYEKCDRDLD
                                          .: *******************************

Exoc4           ELIVQHYTELTTAIRTYQSITERITNSRNKIKQVKENLLSCKMLLHCKRDELRKLWIEGI
Aplodontia_rufa ELIVQHYTELTTAIRTYQSITERITNSRNKIKQVKENLLSCKMLLHCKRDELRKLWIEGI
Xerus_inauris@E ELIVQHYTELTTAIRTYQSITERITNSRNKIKQVKENLLSCKMLLHCKRDELRKLWIEGI
Marmota_monax@E ELIVQHYTELTTAIRTYQSITERITNSRNKIKQVKENLLSCKMLLHCKRDELRKLWIEGI
Spermophilus_da ELIVQHYTELTTAIRTYQSITERITNSRNKIKQVKENLLSCKMLLHCKRDELRKLWIEGI
Ictidomys_tride ELIVQHYTELTTAIRTYQSITERITNSRNKIKQVKENLLSCKMLLHCKRDELRKLWIEGI
Urocitellus_par ELIVQHYTELTTAIRTYQSITERITNSRNKIKQVKENLLSCKMLLHCKRDELRKLWIEGI
Sciurus_vulgari ELIVQHYTELTTAIRTYQSITERITNSRNKIKQVKENLLSCKMLLHCKRDELRKLWIEGI
Sciurus_carolin ELIVQHYTELTTAIRTYQSITERITNSRNKIKQVKENLLSCKMLLHCKRDELRKLWIEGI
Sciurus_niger@E ELIVQHYTELTTAIRTYQSITERITNSRNKIKQVKENLLSCKMLLHCKRDELRKLWIEGI
                ************************************************************

Exoc4           EHKHVLNLLDEIENIKQVPQKLEQCMASKHYLSATDMLVSAVESLEGPLLQVEGLSDLRL
Aplodontia_rufa EHKHVLNLLDEIENIKQVPQKLEQCMASKHYLSATDMLVSAVESLEGPLLQVEGLSDLRL
Xerus_inauris@E EHKHVLNLLDEIENIKQVPQKLEQCMASKHYLSATDMLVSAVESLEGPLLQVEGLSDLRL
Marmota_monax@E EHKHVLNLLDEIENIKQVPQKLEQCMASKHYLSATDMLVSAVESLEGPLLQVEGLSDLRL
Spermophilus_da EHKHVLNLLDEIENIKQVPQKLEQCMASKHYLSATDMLVSAVESLEGPLLQVEGLSDLRL
Ictidomys_tride EHKHVLNLLDEIENIKQVPQKLEQCMVSKHYLSATDMLVSAVESLEGPLLQVEGLSDLRL
Urocitellus_par EHKHVLNLLDEIENIKQVPQKLEQCMASKHYLSATDMLVSAVESLEGPLLQVEGLSDLRL
```

```
Sciurus_vulgari EHKHVLNLLDEIENIKQVPQKLEQCMASKHYLSATDMLVSAVESLEGPLLQVEGLSDLRL
Sciurus_carolin EHKHVLNLLDEIENIKQVPQKLEQCMASKHYLSATDMLVSAVESLEGPLLQVEGLSDLRL
Sciurus_niger@E EHKHVLNLLDEIENIKQVPQKLEQCMASKHYLSATDMLVSAVESLEGPLLQVEGLSDLRL
                **************************.*********************************

Exoc4           ELHSKKMNLHLVLIEELHRHLYIKSTSRVVQRNKEKGKMSSHGKDPSPGPLIDVSNIPTP
Aplodontia_rufa ELHSKKMNLHLVLIDELHRHLYIKSTSRVVQRNKEKGKMSSHGKDPSPGPLIDVSNIPTP
Xerus_inauris@E ELHSKKMNLHLVLIDELHRHLYIKSTSRVVQRNKEKGKMSSHGKDPSPGPLIDVSNIPTP
Marmota_monax@E ELHSKKMNLHLVLIDELHRHLYIKSTSRVVQRNKEKGKMSSHGKDPSPGPLIDVSNIPTP
Spermophilus_da ELHSKKMNLHLVLIDELHRHLYIKSTSRVVQRNKEKGKMSSHGKDPSPGPLIDVSNIPTP
Ictidomys_tride ELHSKKMNLHLVLIDELHRHLYIKSTSRVVQRNKEKGKMSSHGKDPSPGPLIDVSNIPTP
Urocitellus_par ELHSKKMNLHLVLIDELHRHLYIKSTSRVVQRNKEKGKMSSHGKDPSPGPLIDVSNIPTP
Sciurus_vulgari ELHSKKMNLHLVLIDELHRHLYIKSTSRVVQRNKEKGKLSSHGKDPSPGPLIDVSNIPTP
Sciurus_carolin ELHSKKMNLHLVLIDELHRHLYIKSTSRVVQRNKEKGKLSSHGKDPSPGPLIDVSNIPTP
Sciurus_niger@E ELHSKKMNLHLVLIDELHRHLYIKSTSRVVQRNKEKGKLSSHGKDPSPGPLIDVSNIPTP
                **************:***********************:*********************

Exoc4           RKFLDASQYSAAGGSSVREMNLQDVKEDLECDPEENSTLFMGILIQGLARLKKIPETVKA
Aplodontia_rufa RKFLDASQYSAAGGSAVREINLQDLKEDLELDPEENSTLFMGILIKGLAKLKKIPETVKA
Xerus_inauris@E RKFLDASQYSAAGGSAVREINLQDLKEDLELDPEENSTVFMGILIKGLAKLKKIPETVKA
Marmota_monax@E RKFLDASQYSAAGGSAVKEINLQDLKEDLELDPEENSTLFMGILIKGLAKLKKIPETVKA
Spermophilus_da RKFLDASQYSAAGGSSVKEINLQDLKEDLELDPEENSTLFMGILIKGLAKLKKIPETVKA
Ictidomys_tride RKFLDASQYSAAGGSAVKEINLQDLKEDLELDPEENSTLFMGILIKGLAKLKKIPETVKA
Urocitellus_par RKFLDASQYSAAGGSAVKEINLQDLKEDLELDPEENSTLFMGILIKGLAKLKKIPETVKA
Sciurus_vulgari RKFLDASQYSAAGGSAVREINLQDLKEDLELDPEENSTLFMGILIKGLAKLKKVPETVKA
Sciurus_carolin RKFLDASQYSAAGGSAVREINLQDLKEDLELDPEENSTLFMGILIKGLAKLKKVPETVKA
Sciurus_niger@E RKFLDASQYSAAGGSAVREINLQDLKEDLELDPEENSTLFMGILIKGLAKLKKVPETVKA
                ***************:*:*:****:***** *******:******:***:***:******

Exoc4           IKERLEQELKQIVKRSTTQVADSAYQRGESLTVDNQPRLLLELLELLFDKFNAVATAHSV
Aplodontia_rufa IKERLEQELKQIVKRSTTQVADSGYQRGENLIVENQPRLLLELLELLFDKFNAVAAAHSV
Xerus_inauris@E IKERLEQELKQIVKRSTTQVADSGYQRGENLTVENQPRLLLELLELLFDKFNAVATAHSV
Marmota_monax@E IKERLEQELKQIVKRSTTQVADSGYQRGENLTVENQPRLLLELLELLFDKFNAVAAAHSV
Spermophilus_da IKERLEQELKQIVKRSTTQVADSGYQRGENLTVENQPRLLLELLELLFDKFNAVAAAHSV
Ictidomys_tride IKERLEQELKQIVKRSTTQVADSGYQRGENLTVENQPRLLLELLELLFDKFNAVATAHSV
Urocitellus_par IKERLEQELKQIVKRSTTQVADSGYQRGENLTVENQPRLLLELLELLFDKFNAVAAAHSV
Sciurus_vulgari IKERLEQELKQIVKRSTTQVADSGYQRAENLTVENQPRLLLELLELLFDKFNAVAAAHSV
Sciurus_carolin IKERLEQELKQIVKRSTTQVADSGYQRTENLTVENQPRLLLELLELLFDKFNAVAAAHSV
Sciurus_niger@E IKERLEQELKQIVKRSTTQVADSGYQRAENLTVENQPRLLLELLELLFDKFNAVAAAHSV
                ***********************.*** *.* *:*********************:****

Exoc4           VLGYLQDSVGTQLTQQEEIKLYDMADVWVKIQDVLQMLLTEYLDMKNTRTASEPSAQLSY
Aplodontia_rufa VLGYLQDSVGNLQTQQEEIKLYDMADVWVKIQDVLQMLLTEYLDMKNTRTASEPSAQLSY
Xerus_inauris@E VLGYLQDSVGNPLTQQEEIKLYDMADVWVKIQDVLQMLLTEYLDMKNTRTASEPSAQLSY
Marmota_monax@E VLGYLQDSVGNPVTQQEEIKLYDMADVWVKIQDVLQMLLTEYLDMKNTRTASEPSAQLSY
Spermophilus_da VLGYLQDSVGNPPTQQEEIKLYDMADVWVKIQDVLQMLLTEYLDMKNTRTASEPSAQLSY
Ictidomys_tride VLGYLQDSVGNPPTQQEEIKLYDMADVWVKIQDVLQMLLTEYLDMKNTRTASEPSAQLSY
Urocitellus_par VLGYLQDSVGNPPTQQEEIKLYDMADVWVKIQDVLQMLLTEYLDMKNTRTASEPSAQLSY
Sciurus_vulgari VLGYLQDSVGNPLTQQEEIKLYDMADVWVKIQDVLQMLLTEYLDMKNTRTASEPSAQLSY
Sciurus_carolin VLGYLQDSVGNPLTQQEEIKLYDMADVWVKIQDVLQMLLTEYLDMKNTRTASEPSAQLSY
Sciurus_niger@E VLGYLQDSVGNPLTQQEEIKLYDMADVWVKIQDVLQMLLTEYLDMKNTRTASEPSAQLSY
                **********.  ***********************************************

Exoc4           ASTGREFAAFFAKKKPQRPKNSLFKFESSSHAISMSAYLREQRRELYSRSGELQGGPDDN
Aplodontia_rufa ASSGREFAAFFAKKKPQRPKNSLFKFESSSHAISMSAYLREQRRELYSRSGELAGGPDDN
Xerus_inauris@E ASSGREFAAFFAKKKPQRPKNSLFKFESSSHAISMSAYLREQRRELYSRSGELKGGPDDN
```

```
Marmota_monax@E ASSGREFAAFFAKKKPQRPKNSLFKFESSSHAISMSAYLREQRRELYSRSGELKGGPDDN
Spermophilus_da ASSGREFAAFFAKKKPQRPKNSLFKFESSSHAISMSAYLREQRRELYSRSGELKGGPDDN
Ictidomys_tride ASSGREFAAFFAKKKPQRPKNSLFKFESSSHAISMSAYLREQRRELYSRSGELQGGPDDN
Urocitellus_par ASSGREFAAFFAKKKPQRPKNSLFKFESSSHAISMSAYLREQRRELYSRSGELKGGPDDN
Sciurus_vulgari ASSGREFAAFFAKKKPQRPKNSLFKFESSSHAISMSAYLREQRRELYSRSGELKGGPDDN
Sciurus_carolin ASSGREFAAFFAKKKPQRPKNSLFKFESSSHAISMSAYLREQRRELYSRSGELKGGPDDN
Sciurus_niger@E ASSGREFAAFFAKKKPQRPKNSLFKFESSSHAISMSAYLREQRRELYSRSGELKGGPDDN
                **:************************************************** ******

Exoc4           LIEGGGTKFVCKPGARNITVIFHPLLRFIQEIEHALGLGPAKQCPLREFLTVYIKSIFLN
Aplodontia_rufa LIEGGGTKFVCKPGARNITVIFHPLLRFIQEIEHALGLGPAKQCPLREFLTVYIKTIFLN
Xerus_inauris@E LIEGGGTKFVCKPGARNITVIFHPLLRFIQEIEHALGLGPAKQCPLREFLTMYIKNIFLN
Marmota_monax@E LIEGGGTKFVCKPGARNITVIFHPLLRFIQEIEHALGLGPAKQCPLREFLTMYIKNIFLS
Spermophilus_da LIEGGGTKFVCKPGARNITVIFHPLLRFIQEIEHALGLGPAKQCPLREFLTMYIKNIFLS
Ictidomys_tride LIEGGGTKFVCKPGARNITVIFHPLLRFIQEIEHALGLGPAKQCPLREFLTMYIKNIFLS
Urocitellus_par LIEGGGTKFVCKPGARNITVIFHPLLRFIQEIEHALGLGPAKQCPLREFLTMYIKNIFLS
Sciurus_vulgari LIEGGGTKFVCKPGARNITVIFHPLLRFIQEIEHALGLGPAKQCPLREFLTEYIKNFFLN
Sciurus_carolin LIEGGGTKFVCKPGARNITVIFHPLLRFIQEIEHALGLGPAKQCPLREFLTVYIKTIFLN
Sciurus_niger@E LIEGGGTKFVCKPGARNITVIFHPLLRFIQEIEHALGLGPAKQCPLREFLTVYIKTIFLN
                *************************************************** ***.:**.

Exoc4           QVLAEINKEIEGVTKTSDPLKILANADTMKVLGVQRPLLQSTIIVEKTVQDLMNLMHDLS
Aplodontia_rufa QVLAEINKEIEGVTKTSDPLKILANADTMKVLGVQRPLLQSTIIVEKTVQDLINLMRDLS
Xerus_inauris@E QVLAEINKEIEGVTKTSDPLKILANADTMKVLGVQRPLLQSTIIVEKTVQDLMNLMHDLS
Marmota_monax@E QVLAEINKEIEGVTKTSDPLKILANADTMKVLGVQRPLLQSTIIVEKTVQDLMNLMHDLS
Spermophilus_da QVLAEINKEIEGVTKTSDPLKILANADTMKVLGVQRPLLQSTIIVEKTVQDLMNLMHDLS
Ictidomys_tride QVLAEINKEIEGVTKTSDPLKILANADTMKVLGVQRPLLQSTIIVEKTVQDLMNLMHDLS
Urocitellus_par QVLAEINKEIEGVTKTSDPLKILANADTMKVLGVQRPLLQSTIIVEKTVQDLMNLMHDLS
Sciurus_vulgari QVLAEINKEIEGVTKTSDPLKILANADTMKVLGVQRPLLQSTIIVEKTVQDLMNLMHDLS
Sciurus_carolin QVLAEINKEIEGVTKTSDPLKILANADTMKVLGVQRPLLQSTIIVEKTVQDLMNLMHDLS
Sciurus_niger@E QVLAEINKEIEGVTKTSDPLKILANADTMKVLGVQRPLLQSTIIVEKTVQDLMNLMHDLS
                ****************************************************:***:***

Exoc4           AYSDQFLNMVCVKLQEYKDTCSTAYRGIVQSEEKLVISASWAKDDDISRLLKSLPNWTNM
Aplodontia_rufa AYSDQFLHMVCVKLQEYKDTCSAAYRGIVQSEEKLVISASWAKDDDISRLLKSLPNWINM
Xerus_inauris@E AYSDQFLNMVCVKLQEYKDTCSAAYRGIVQSEEKLVISASWAKDDDISRLLKSLPNWINM
Marmota_monax@E AYSDQFLNMVCVKLQEYKDTCSAAYRGIVQSEEKLVISASWAKDDDISRLLKSLPNWINM
Spermophilus_da AYSDQFLNMVCVKLQEYKDTCSAAYRGIVQSEEKLVISASWAKDDDISRLLKSLPNWINM
Ictidomys_tride AYSDQFLNMVCVKLQEYKDTCSAAYRGIVQSEEKLVISASWAKDDDISRLLKSLPNWINM
Urocitellus_par AYSDQFLNMVCVKLQEYKDTCSAAYRGIVQSEEKLVISASWAKDDDISRLLKSLPNWINM
Sciurus_vulgari AYSDQFLKMVCVKLQEYKDTCAAAYRGIVQSEEKLVISASWAKDDDISRLLKSLPNWINM
Sciurus_carolin AYSDQFLNMVCVKLQEYKDTCSAAYRGIVQSEEKLVISASWAKDDDISRLLKSLPNWINM
Sciurus_niger@E AYSDQFLNMVCVKLQEYKDTCSAAYRGIVQSEEKLVISASWAKDDDISRLLKSLPNWINM
                *******:*************::********************************** **

Exoc4           AQPKQLRPKREEEEDFIRAAFGKESEVLIGNLGDKLIPPQDILRDVSDLKALANMHESLE
Aplodontia_rufa AQPKQLRPKREEEEDFIRAAFGKESEVLIGNLGDKLIPPQDILRDVSDLKALANMHESLE
Xerus_inauris@E AQPKQLRPKREEEEDFIRAAFGKESEVLIGNLGDKLIPPQDILRDVSDLKALANMHESLE
Marmota_monax@E AQPKQLRPKREEEEDFIRAAFGKESEVLIGNLGDKLIPPQDILRDVSDLKALANMHESLE
Spermophilus_da AQPKQLRPKREEEEDFIRAAFGKESEVLIGNLGDKLIPPQDILRDVSDLKALANMHESLE
Ictidomys_tride AQPKQLRPKREEEEDFIRAAFGKESEVLIGNLGDKLIPPQDILRDVSDLKALANMHESLE
Urocitellus_par AQPKQLRPKREEEEDFIRAAFGKESEVLIGNLGDKLIPPQDILRDVSDLKALANMHESLE
Sciurus_vulgari AQPKQLRPKREEEEDFIRAAFGKESEVLIGNLGDKLIPPQDILRDVSDLKALANMHESLE
Sciurus_carolin AQPKQLRPKREEEEDFIRAAFGKESEVLIGNLGDKLIPPQDILRDVSDLKALANMHESLE
Sciurus_niger@E AQPKQLRPKREEEEDFIRAAFGKESEVLIGNLGDKLIPPQDILRDVSDLKALANMHESLE
                ************************************************************
```

```
Exoc4            WLAGRTKSAFSNLSTSQMLSPAQESHVNMDLPPVSEQIMQTLSELAKTFQDMADRCLLVL
Aplodontia_rufa  WLAGRAKSAFSNLSTSQVLSPAQESHMNMDLPPVSEQILQTLSELAKSFQDMADRCLLVL
Xerus_inauris@E  WLAGRTKSAFANLSTSQVLSPAQESHVNMDLPPVSEQIMQTLSELAKSFQDMADRCLLVL
Marmota_monax@E  WLAGRTKSAFSNLSTSQVLSPAQESHVNMDLPPVSEQIMQTLSELAKSFQDMADRCLLVL
Spermophilus_da  WLAGRTKSAFSNLSTSQVLSPAQESHVNMDLPPVSEQIMQTLSELAKSFQDMADRCLLVL
Ictidomys_tride  WLAGRTKSAFSNLSTSQVLSPAQESHVNMDLPPVSEQIMQTLSELAKSFQDMADRCLLVL
Urocitellus_par  WLAGRTKSAFSNLSTSQVLSPAQESHVNMDLPPVSEQIMQTLSELAKSFQDMADRCLLVL
Sciurus_vulgari  WLAGRTKSAFSNLSTSQVLSPAQESHVNMDLPPVSEQIMQTLSELAKSFQDMADRCLLVL
Sciurus_carolin  WLAGRTKSAFSNLSTSQVLSPAQESHVNMDLPPVSEQIMQTLSELAKSFQDMADRCLLVL
Sciurus_niger@E  WLAGRTKSAFSNLSTSQVLSPAQESHVNMDLPPVSEQIMQTLSELAKSFQDMADRCLLVL
                 *****:****:******:********:***********:********:************

Exoc4            HLEVRVHCFHYLIPLAKEGNYAIVANVESMDYDPLVVKLNKDISAMEEAMSASLQQHKFQ
Aplodontia_rufa  HLEVRVHCFHYLIPLAKEGNYAIVANVESMDYDPLVVKLNKDISAIEEAMSASLQQHKFQ
Xerus_inauris@E  HLEVRVHCFHYLIPLAKEGNYAIVANVESMDYDPLVVKLNKDISAIEEAMSASLQQHKFQ
Marmota_monax@E  HLEVRVHCFHYLIPLAKEGNYAIVANVESMDYDPLVVKLNKDISAIEEAMSASLQQHKFQ
Spermophilus_da  HLEVRVHCFHYLIPLAKEGNYAIVANVESMDYDPLVVKLNKDISAIEEAMSASLQQHKFQ
Ictidomys_tride  HLEVRVHCFHYLIPLAKEGNYAIVANVESMDYDPLVVKLNKDISAIEEAMSASLQQHKFQ
Urocitellus_par  HLEVRVHCFHYLIPLAKEGNYAIVANVESMDYDPLVVKLNKDISAIEEAMSASLQQHKFQ
Sciurus_vulgari  HLEVRVHCFHYLIPLAKEGNYAIVANVESMDYDPLVVKLNKDISAIEEAMSASLQQHKFQ
Sciurus_carolin  HLEVRVHCFHYLIPLAKEGNYAIVANVESMDYDPLVVKLNKDISAIEEAMSASLQQHKFQ
Sciurus_niger@E  HLEVRVHCFHYLIPLAKEGNYAIVANVESMDYDPLVVKLNKDISAIEEAMSASLQQHKFQ
                 *********************************************:**************

Exoc4            YIFEGLGHLISCILINGAQYFRRISESGIKKMCRNIFVLQQNLTNITMSREADLDFARQY
Aplodontia_rufa  YIFEGLGHLISCILINGAQYFRRISESGIKKMCRNIFVLQQNLTNITMSREADLDFARQY
Xerus_inauris@E  YIFEGLGHLISCILINGAQYFRRISESGIKKMCRNIFVLQQNLTNITMSREADLDFARQY
Marmota_monax@E  YIFEGLGHLISCILINGAQYFRRISESGIKKMCRNIFVLQQNLTNITMSREADLDFARQY
Spermophilus_da  YIFEGLGHLISCILINGAQYFRRISESGIKKMCRNIFVLQQNLTNITMSREADLDFARQY
Ictidomys_tride  YIFEGLGHLISCILINGAQYFRRISESGIKKMCRNIFVLQQNLTNITMSREADLDFARQY
Urocitellus_par  YIFEGLGHLISCILINGAQYFRRISESGIKKMCRNIFVLQQNLTNITMSREADLDFARQY
Sciurus_vulgari  YIFEGLGHLISCILINGAQYFRRISESGIKKMCRNIFVLQQNLTNITMSREADLDFARQY
Sciurus_carolin  YIFEGLGHLISCILINGAQYFRRISESGIKKMCRNIFVLQQNLTNITMSREADLDFARQY
Sciurus_niger@E  YIFEGLGHLISCILINGAQYFRRISESGIKKMCRNIFVLQQNLTNITMSREADLDFARQY
                 ************************************************************

Exoc4            YEMLYNTADELLNLVVDQGVKYTELEYIHALTLLHRSQTGVGDQTTQNTRLQRLKEIICE
Aplodontia_rufa  YEMLYNTADELLNLVVDQGVKYTELEYIHALTLLHRSQTGVGDQTTQNTRLQRLKEIICE
Xerus_inauris@E  YEMLYNTADELLNLVVDQGVKYTELEYIHALTLLHRSQTGVGDQSTQNMRLQRLKEIICE
Marmota_monax@E  YEMLYNTADELLNLVVDQGVKYTELEYIHALTLLHRSQTGVGDQSTQNTRLQRLKEIICE
Spermophilus_da  YEMLYNTADELLNLVVDQGVKYTELEYIHALTLLHRSQTGVGDQSTQNTRLQRLKEIICE
Ictidomys_tride  YEMLYNTADELLNLVVDQGVKYTELEYIHALTLLHRSQTGVGDQSTQNTRLQRLKEIICE
Urocitellus_par  YEMLYNTADELLNLVVDQGVKYTELEYIHALTLLHRSQTGVGDQSTQNTRLQRLKEIICE
Sciurus_vulgari  YEMLYNTADELLNLVVDQGVKYTELEYIHALTLLHRSQTGVGDQTTQNTRLQRLKEIICE
Sciurus_carolin  YEMLYNTADELLNLVVDQGVKYTELEYIHALTLLHRSQTGVGDQTTQNTRLQRLKEIICE
Sciurus_niger@E  YEMLYNTADELLNLLVDQGVKYTELEYIHALTLLHRSQTGVGDQTTQNTRLQRLKEIICE
                 **************:*****************************:*** ***********

Exoc4            QAAIKQATKDKKITTVZ
Aplodontia_rufa  QAAIKQATKDKKITTV-
Xerus_inauris@E  QAAIKQATKDKKITTV-
Marmota_monax@E  QAAIKQATKDKKITTV-
Spermophilus_da  QAAIKQATKDKKITTV-
Ictidomys_tride  QAAIKQATKDKKITTV-
Urocitellus_par  QAAIKQATKDKKITTV-
```

```
Sciurus_vulgari QAAIKQATKDKKITTV-
Sciurus_carolin QAAIKQATKDKKITTV-
Sciurus_niger@E QAAIKQATKDKKITTV-
                ****************
```

FAM204A encodes family with sequence similarity 204 member A, a protein. Grabek *et al*. (2019) associated SNPs at the promoter for this gene with the onset of hibernation in 13-lined ground squirrel *Ictidomys tridecemlineatus*.

Within a 239-amino acid sequence alignment for FAM204A, we observed six point-differences among sciurids, including a two-amino acid deletion at positions 28-29. Among all species, there was an indel at positions 28-30. *Ictidomys tridecemlineatus* and *U. parryii* had a 28-amino acid indel tract at positions 89-115 that did not resemble the aligned sequences of other species, and a two-amino acid insertion at positions 137-138.

```
Fam204a         MWSGLLPPGLNESDVESDSEDEIKLENPEPSEHNLQEDGKTGSSTKPAVSDFPTGQPETE
Aplodontia_rufa MWSGLLPPGLNESDVESNSEDEITLEK---SELNLQEDGKTGSSTKPAVSDFPTGQPETE
Xerus_inauris@F -WSGLLPPGLRERGVDSDSGEEATLGSVEPSEHNLQEDGKTGSSTKPAVSDFPTGQPETE
Marmota_monax@F MWSGLLPPGLNESDVESNSEDEATLEN--PSELNLQEDKEDGTSRKTEISDFPADGPKTE
Spermophilus_da MWSGLLPPGLNESDVESNSEDEATLEK---SELNLQEDGKTGSSTKPEISDFPADGPKIE
Ictidomys_tride MWSGLLPPGLNESDVESNSEDEATLEK---SELNLQEDKEDGTSTKTEISDFPADGPKIE
Urocitellus_par MWSGLLPPGLNESDVESNSEDEATLEK---SELNLQEDKEDGTSRKTEISSFPADGPKTE
Sciurus_vulgari MWSGLLPPGLNESDVESNSEDEITLEKPE-SELNLQEDKEDGSSTKPEVSDFPTGGPETE
Sciurus_carolin MWSGLLPPGLNESDVESDSEDEITLEK---SELNLQEDKEDGSSRKPEVSDFPTGGPKTE
Sciurus_niger@F MWSGLLPPGLNESDVESNSEDEITLEKPE-SELNLQEDKEDGSSRKPEVSDFPTGGPKTE
                 *********.* .*:*:* :* .* .   ** ***** : *:* *. :*.**:. *: *

Fam204a         TEADADAYEKCPSGIPLNIWNKFQELHKKNSEQKNSTPRFRQKKRKRSKKGKLKNEKESH
Aplodontia_rufa TEADADAYEKCPSGIPLNIWNKFQELHKKNSEQKNSTPRFRQKKRKRSKKGKLKNEKESH
Xerus_inauris@F TEADADAYEKCPSGIPL-IWNKFQELHKKNSEQKNSTPRFRQKKRKRSKKGKLKNEKESH
Marmota_monax@F TEANADAYEECPSGIPLNLWNKFQELHKNHSEQKISTSRFRGKKRKRSRKDKLKNEKESH
Spermophilus_da TEANADAYEECPSGIPLNLWNKFQELHKKHSEQKNSTSRFRGKKRKRSRKDKLKNEKESH
Ictidomys_tride TEANADAYEECPSGIPLNLWNKFQELHK---DILNRKPQTSEGKR----------ENVPD
Urocitellus_par TEANADAYEECPSGIPLNLWNKFQELHK---DILNRKPQTSEGKR----------ENVPD
Sciurus_vulgari TEANADAYEKCPSGIPLNIWNKFQELHKKNSEQKNSTPRFRQKKRKRSKKGKLKNEKESH
Sciurus_carolin TEANADAYEECPSGIPLNIWNKFQELHKKNSEQKNSTPRFRQKKRKRSKKGKLKNEKESH
Sciurus_niger@F TEANADAYEECPSGIPLNIWNKFQELHKKNSEQKNSTPRFRQKKRKRSKKGKLKNEKESH
                ***:*****:******* :*********   :    ..:    **          *: ..

Fam204a         SEQSSNETQWEELTQY--FGANDRFEPPVKQKKVEKSGLEKRIDQAVEEWDVEKAEELSN
Aplodontia_rufa SEQSSNETQWEELTQY--FGANDRFEPPVKQKKVEKSGLEKRIDQAVEEWDVEKAEELSN
Xerus_inauris@F SEQSSNETQWKELTQY--FGANDRFEPPVKKKKVDKSGLEKRIDQAVEEWDVEKAEELSN
Marmota_monax@F SEQPLNETQWEELTQY--FGANDRFEPPVKKKKVEKSGLEKRIDQAVEEWDVEKAEELSN
Spermophilus_da SEQSSNETQWKELTQY--FGANDRFEPPVKQKKVEKSGLEKRIDQAVEEWDVEKAEELSN
Ictidomys_tride NEQSLNEIQ-KELTYYLVFGCSDRFEPPVKKKKIDKSGLEKKIVQAVEE-NVEKPEEFSN
Urocitellus_par NEQSLNEIQ-KELTYYLVFGCSDRFEPPVKKKKIDKSGLEKKIVQAVEE-NVEKPEEFSN
Sciurus_vulgari SEQSSNETQWEELTQY--FGANDRFEPPVKQKKVEKSGLEKRIDQAVEEWDVEKAEELSN
Sciurus_carolin SEQSSNETQWEELTQY--FGANDRFEPPVKQKKVEKSGLEKRIDQAVEEWDVEKAEELSN
Sciurus_niger@F SEQSSNETQWEELTQY--FGANDRFEPPVKQKKVEKSGLEKRIDQAVEEWDVEKAEELSN
                .**. ** * :*** *  **..********:**::******:* ***** :***.**:**

Fam204a         QLATRELGVKIAKAIACHKFVKAKKEAENSQAARKKKKLAWGFEAKKRWETKSNMGYMZ
Aplodontia_rufa QLATRELGVKIAKAIACHKFVKAKKEAENSQAARKKKKLAWGFEAKKRWETQSNMGYM-
Xerus_inauris@F QLATRELGVKIAKAIACHKFVKAKKEAENSQAARKKKKLAWGFEAKKRWETKSNMGYMQ
Marmota_monax@F QLATRELGVKIAKAIACHKFVKAKKEAENSQAARKKKKLAWGFEAKKRWETKSNMGYM-
```

```
Spermophilus_da QLATRELGVKIAKAIACHKFVKAKKEAENSQAARKKKKLAWGFEAKRRWETKSNMGYM-
Ictidomys_tride RLATWELGVKIAKAIACHKFVKAKKEAENSQAARKKKKLAWG-----------------
Urocitellus_par RLATWELGVKIAKAIACHKFVKAKKEAENSQAARKKKKLAWG-----------------
Sciurus_vulgari QLATRELGVKIAKAIACHKFVKAKKEAENSQAARKKKKLAWGFEAKKRWETKSNMGYM-
Sciurus_carolin QLATRELGVKIAKAIACHKFVKAKKEAENSQAARKKKKLAWGFEAKKRWETKSNMGYM-
Sciurus_niger@F QLATRELGVKIAKAIACHKFVKAKKEAENSQAARKKKKLAWGFEAKKRWETKSNMGYM-
                :*** ************************************
```

Literature Cited

Grabek KR, Cooke TF, Epperson LE, Spees KK, Cabral GF, *et al*. 2019. Genetic variation drives seasonal onset of hibernation in the 13-lined ground squirrel. Comm Biol. 2: 1-13.
